# Co-targeting BCL-X_L_ and MCL-1 with DT2216 and AZD8055 synergistically inhibit small-cell lung cancer growth without causing on-target toxicities in mice

**DOI:** 10.1038/s41420-022-01296-8

**Published:** 2023-01-02

**Authors:** Sajid Khan, Patrick Kellish, Nick Connis, Dinesh Thummuri, Janet Wiegand, Peiyi Zhang, Xuan Zhang, Vivekananda Budamagunta, Nan Hua, Yang Yang, Umasankar De, Lingtao Jin, Weizhou Zhang, Guangrong Zheng, Robert Hromas, Christine Hann, Maria Zajac-Kaye, Frederic J. Kaye, Daohong Zhou

**Affiliations:** 1 Department of Biochemistry & Structural Biology, Long School of Medicine, University of Texas Health Science Center at San Antonio, San Antonio, TX USA; 2 Mays Cancer Center, University of Texas Health Science Center at San Antonio, San Antonio, TX USA; 3 Department of Pharmacodynamics, College of Pharmacy, University of Florida, Gainesville, FL USA; 4 Department of Anatomy & Cell Biology, College of Medicine, University of Florida, Gainesville, FL USA; 5 Department of Pediatrics, College of Medicine, University of Florida, Gainesville, FL, USA; 6 Department of Oncology, School of Medicine, Johns Hopkins University, Baltimore, MD USA; 7 Department of Medicinal Chemistry, College of Pharmacy, University of Florida, Gainesville, FL USA; 8 Genetics and Genomics Graduate Program, Genetics Institute, College of Medicine, University of Florida, Gainesville, FL USA; 9 Department of Neuroscience, College of Medicine, University of Florida, Gainesville, FL USA; 10 Department of Pathology, Immunology and Laboratory Medicine, College of Medicine, University of Florida, Gainesville, FL USA; 11 Department of Molecular Medicine, Long School of Medicine, University of Texas Health Science Center at San Antonio, San Antonio, TX USA; 12 Department of Medicine, Long School of Medicine, University of Texas Health Science Center at San Antonio, San Antonio, TX USA; 13 Division of Hematology and Oncology, Department of Medicine, College of Medicine, University of Florida, Gainesville, FL USA

**Keywords:** Small-cell lung cancer, Drug development

## Abstract

Small-cell lung cancer (SCLC) is an aggressive malignancy with limited therapeutic options. The dismal prognosis in SCLC is in part associated with an upregulation of BCL-2 family anti-apoptotic proteins, including BCL-X_L_ and MCL-1. Unfortunately, the currently available inhibitors of BCL-2 family anti-apoptotic proteins, except BCL-2 inhibitors, are not clinically relevant because of various on-target toxicities. We, therefore, aimed to develop an effective and safe strategy targeting these anti-apoptotic proteins with DT2216 (our platelet-sparing BCL-X_L_ degrader) and AZD8055 (an mTOR inhibitor) to avoid associated on-target toxicities while synergistically optimizing tumor response. Through BH3 mimetic screening, we identified a subset of SCLC cell lines that is co-dependent on BCL-X_L_ and MCL-1. After screening inhibitors of selected tumorigenic pathways, we found that AZD8055 selectively downregulates MCL-1 in SCLC cells and its combination with DT2216 synergistically killed BCL-X_L_/MCL-1 co-dependent SCLC cells, but not normal cells. Mechanistically, the combination caused BCL-X_L_ degradation and suppression of MCL-1 expression, and thus disrupted MCL-1 interaction with BIM leading to an enhanced apoptotic induction. In vivo, the DT2216 + AZD8055 combination significantly inhibited the growth of cell line-derived and patient-derived xenografts and reduced tumor burden accompanied by increased survival in a genetically engineered mouse model of SCLC without causing appreciable thrombocytopenia or other normal tissue injuries. Thus, these preclinical findings lay a strong foundation for future clinical studies to test DT2216 + mTOR inhibitor combinations in a subset of SCLC patients whose tumors are co-driven by BCL-X_L_ and MCL-1.

## Introduction

Small-cell lung cancer (SCLC) is a difficult-to-treat subtype of pulmonary carcinomas with a 5-year survival rate of ~5% and median survival of less than a year [1, 2]. First-line treatment for SCLC with combinations of a platinum-based agent (cisplatin or carboplatin) and etoposide (a topoisomerase-II inhibitor) has remained largely unchanged for almost 35 years. Recently, a triplet of atezolizumab (a PD-L1 antibody), carboplatin, and etoposide was approved as first-line therapy for extensive-stage SCLC but provides only a moderate (~2 months) increase in overall survival compared to chemotherapy alone [3, 4]. More importantly, the SCLC tumors are initially responsive to chemotherapy, however, relapse occurs in a majority (>80%) of cases, and there is no effective second-line treatment for relapsed SCLC [4, 5]. There is thus an urgent and unmet need to find newer treatment strategies to effectively treat SCLC.

SCLC has historically been treated as a homogeneous disease. However, it has been recently demonstrated that inter- and intra-tumoral heterogeneity occurs in SCLC, is primarily responsible for reduced therapeutic efficacy and resistance [6–9]. Since unique resistance mechanisms occur in response to specific therapies, specific oncoproteins and/or pathways need to be defined and targeted for effectively treating SCLC. Aberrant expression of anti-apoptotic BCL-2 family proteins has also been shown to account for intratumoral heterogeneity and therapeutic resistance in SCLC [6, 10]. This has been therapeutically exploited with a dual BCL-X_L_/BCL-2 inhibitor ABT263 (navitoclax) that shows high efficacy in preclinical models of SCLC [11, 12]. Unfortunately, the single-agent efficacy of navitoclax against SCLC in phase-II clinical trials was limited [13] because of the intratumoral heterogeneity and dependence of some of the cells within a tumor on other BCL-2 family proteins such as MCL-1. More importantly, the clinical use of ABT263 is hampered by dose-limiting severe thrombocytopenia caused by BCL-X_L_ inhibition [14, 15]. We are now able to safely target BCL-X_L_ using proteolysis targeting chimeras (PROTACs, such as DT2216) without causing appreciable platelet toxicity (thrombocytopenia) as recently reported by our group [16–20].

In this study, we aimed to develop a synergistic and safe therapeutic strategy for SCLC. This was achieved first by profiling the heterogeneity of survival dependence of a panel of SCLC cell lines on the BCL-2 family anti-apoptotic proteins by BH3 mimetic screening, where we used BH3 mimetics to target specific BCL-2 anti-apoptotic proteins followed by measurement of cell viability [21]. We defined a subset of SCLC cell lines that are co-dependent on BCL-X_L_ and MCL-1 for survival and focused on testing the ability to target these cell lines effectively and safely. Currently, such tumors cannot be safely targeted with available inhibitors. For example, MCL-1 inhibition causes severe cardiotoxicity, and co-targeting BCL-X_L_ and MCL-1 with commercially available inhibitors further exacerbates normal tissue injury and causes lethality [22–24]. Therefore, we need to develop an alternate strategy to selectively suppress BCL-X_L_ and MCL-1 in co-dependent SCLC tumors to avoid their on-target and dose-limiting toxicities. We have devised a safer strategy to target these tumors as we found that BCL-X_L_/MCL-1 co-dependent SCLC cells can be synergistically killed with a combination of DT2216 (a selective BCL-X_L_ PROTAC degrader) and AZD8055 (an mTOR inhibitor). This was achieved by selective degradation of BCL-X_L_ and inhibition of MCL-1 expression in tumor cells with DT2216 and AZD8055, respectively, therefore reducing on-target toxicity to platelets and other normal cells. In vivo, the combination of DT2216 and AZD8055 strongly inhibited the growth of cell line-derived and patient-derived xenograft (PDX) tumors and reduced tumor burden as well as increased survival of a murine *Rb1/p53/p130* SCLC genetically engineered mouse (GEM) model without causing appreciable thrombocytopenia or other normal tissue injuries.

## Results

### BH3 mimetic screening identifies DT2216 sensitive and resistant SCLC cells in vitro

**Fig. 1:**
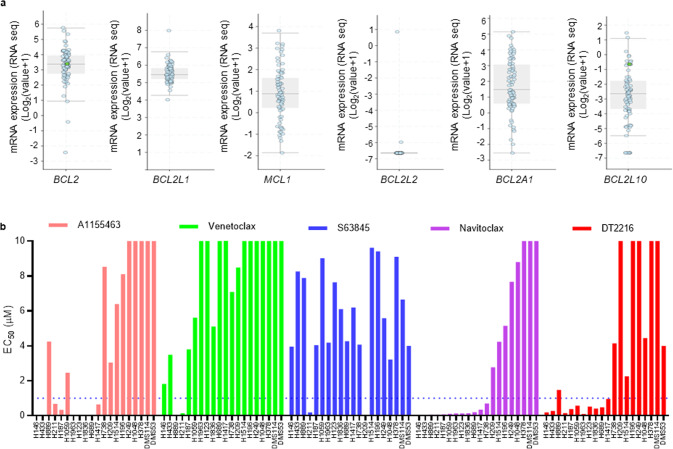
The SCLC cell lines show differential survival dependence on BCL-2 family proteins. **a** The mRNA expression of BCL2 family anti-apoptotic genes as obtained from U Cologne study via cBioPortal. The mRNA expressions are depicted as Log2 (value + 1) from the RNA sequencing (RNA seq) dataset (*n* = 81 patient samples). **b** The EC_50_ values of A1155463 (a selective BCL-X_L_ inhibitor), venetoclax (a selective BCL-2 inhibitor), S63845 (a selective MCL-1 inhibitor), navitoclax (a BCL-X_L_/BCL-2 dual inhibitor) and DT2216 (a selective BCL-X_L_ PROTAC degrader) were derived from % viability of indicated human SCLC cell lines after they were incubated with increasing concentrations of these inhibitors for 72 h. The data are presented from a single experiment performed in three replicate cell cultures. Similar results were obtained in two additional experiments performed with H146 and H378, and one additional independent experiment performed with H187, H211, and H209 cell lines. When an inhibitor showed an EC_50_ of ≤1 μM in any given cell line, the cell line was considered as sensitive to that inhibitor and dependent on its target BCL-2 family protein.

We first explored the mRNA expression of anti-apoptotic BCL-2 family genes in patient tumors *via* cBioPortal [25]. These data showed that *BCL2*, *BCL2L1* (BCL-X_L_ coding gene), *MCL1*, and *BCL2A1* (BFL-1 coding gene) are abundantly expressed in most SCLC patients’ tumors (Fig. 1a). Unfortunately, no healthy lung tissue transcriptomics data were available for a side-by-side comparison to show whether or not these genes were overexpressed in SCLC. Therefore, to establish the importance of these BCL-2 family of anti-apoptotic proteins in SCLC, we systematically evaluated the effects of inhibitors of BCL-2 family anti-apoptotic proteins, i.e., A1155463 (a selective BCL-X_L_ inhibitor), venetoclax (a selective BCL-2 inhibitor), S63845 (a selective MCL-1 inhibitor) and navitoclax (a BCL-X_L_/BCL-2 inhibitor) on the viability of a panel of 20 SCLC cell lines, which was named as the BH3 mimetic screening [21]. The cell lines with EC_50_ < 1 µM for a particular inhibitor were considered sensitive. SCLC cell lines showed differential dependence on different BCL-2 family anti-apoptotic proteins, with a majority of them depending on BCL-X_L_ (9/20, 45%), while some depended on a combination of BCL-X_L_ and BCL-2 (3/20, 15%), and only a few depended on BCL-2 (2/20, 10%) or MCL-1 (2/20, 10%), as indicated by their sensitivity to these specific inhibitors (Fig. 1b). One cell line (H211) was found to be sensitive to all of these inhibitors indicating that all three, i.e., BCL-X_L_, BCL-2, and MCL-1 are important for its survival. These findings are corroborated by the transcriptomic profiling suggesting that BCL-X_L_ is one of the highly expressed BCL-2 family anti-apoptotic proteins, and therefore, a promising therapeutic target in SCLC tumors [10–12]. Further, we evaluated the activity of our BCL-X_L_ PROTAC degrader DT2216 on the viability of these SCLC cell lines where 50% (10/20) cell lines were found to be sensitive to DT2216 with an EC_50_ of <1 µM. All of the cell lines that were found to be sensitive to the BCL-X_L_ inhibitor were sensitive to DT2216 as well. Interestingly, the H1059 cell line was not sensitive to the BCL-X_L_ inhibitor but was found to be sensitive to DT2216. Six of the cell lines did not respond to any of these inhibitors or DT2216 suggesting that these may co-depend on a combination of either BCL-X_L_/MCL-1 or BCL-2/MCL-1 or BCL-X_L_/BCL-2/MCL-1 (Fig. 1b). These results suggest that BCL-X_L_ is a key pro-survival protein in SCLC and DT2216 might have a broad-spectrum antitumor activity than a BCL-X_L_ inhibitor in killing BCL-X_L_-dependent SCLC cells.

### A combination of MCL-1 inhibitor with BCL-X_L_ inhibitor or PROTAC is not selective to tumor cells

**Fig. 2:**
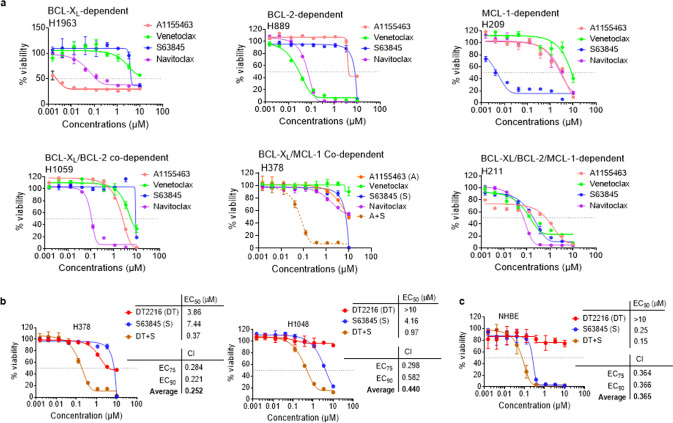
A subset of SCLC cell lines is co-dependent on BCL-X_L_ and MCL-1. **a** Viability graphs of representative SCLC cell lines showing dependence on individual or a combination of BCL-2 family anti-apoptotic proteins. The data are presented as mean ± SD from a single experiment (*n* = 3 replicate cell cultures). Similar results were obtained in one additional independent experiment performed with H209 and H211, and two additional independent experiments performed with H378 cells. **b**, **c** Viability of H378 and H1048 SCLC (**b**) and NHBE (**c**) cells after they were treated with increasing concentrations of DT2216 (DT) or S63845 (S) or their combination (1:1 ratio) for 72 h. EC_50_ and CI values are shown. The data are presented as mean ± SD from a representative experiment (*n* = 3 replicate cell cultures). Similar results were obtained in one additional independent experiment.

In the BH3 mimetic screening, SCLC cell lines showed varying responses to the inhibitors of different BCL-2 family anti-apoptotic proteins. Based on these dependencies, we divided SCLC cell lines into different categories; 1) primarily dependent on BCL-X_L_ (such as H1963), 2) mainly dependent on BCL-2 (such as H889), 3) mostly dependent on MCL-1 (such as H209), 4) BCL-X_L_ and BCL-2 co-dependent (such as H1059), 5) BCL-X_L_ and MCL-1 co-dependent (such as H378), and 6) BCL-X_L_, BCL-2, and MCL-1 dependent (such as H211) (Fig. 2a). When two or more proteins were needed to be simultaneously inhibited in order to sensitize a particular cell line, we called it co-dependent on those proteins. For example, H1059 cells were only sensitive upon simultaneous inhibition of BCL-X_L_ and BCL-2 using navitoclax, therefore, we categorized them as BCL-X_L_ and BCL-2 co-dependent. Similarly, H378 cells were sensitive to simultaneous inhibition of BCL-X_L_ and MCL-1, and therefore, categorized as BCL-X_L_/MCL-1 co-dependent. These results indicate that SCLC cells are heterogeneous in their dependencies on BCL-2 anti-apoptotic proteins.

Next, we focused on targeting BCL-X_L_ and MCL-1 co-dependent cells, as these types of SCLC tumors are highly resistant to chemotherapy and cannot be safely eradicated with the currently available inhibitors of BCL-2 family proteins because the combination of BCL-X_L_ and MCL-1 inhibitors causes severe normal tissue toxicities and lethality in mice [23, 24, 26]. This was confirmed by our study showing that a combination of DT2216 or A1155463 with MCL-1 inhibitor (S63845) synergistically kills not only tumor cells (Fig. 2b), but also normal cells from different tissue origins such as human bronchial epithelial cells (NHBE), colon fibroblasts (CCD-18Co) and lung fibroblasts (WI38) (Fig. 2c; supplementary Fig. 1a). Of note, S63845 treatment alone was also highly toxic to NHBE cells, which indicates that these cells primarily depend on MCL-1 for survival. These findings do not support using the combination of a BCL-X_L_ inhibitor/PROTAC and an MCL-1 inhibitor to treat BCL-X_L_ and MCL-1 co-dependent SCLC in the clinic.

### DT2216+AZD8055 combination synergistically kills BCL-X_L_/MCL-1 co-dependent SCLC cells through degradation of BCL-X_L_ and suppression of MCL-1 expression, respectively, in a tumor cell-selective manner

**Fig. 3:**
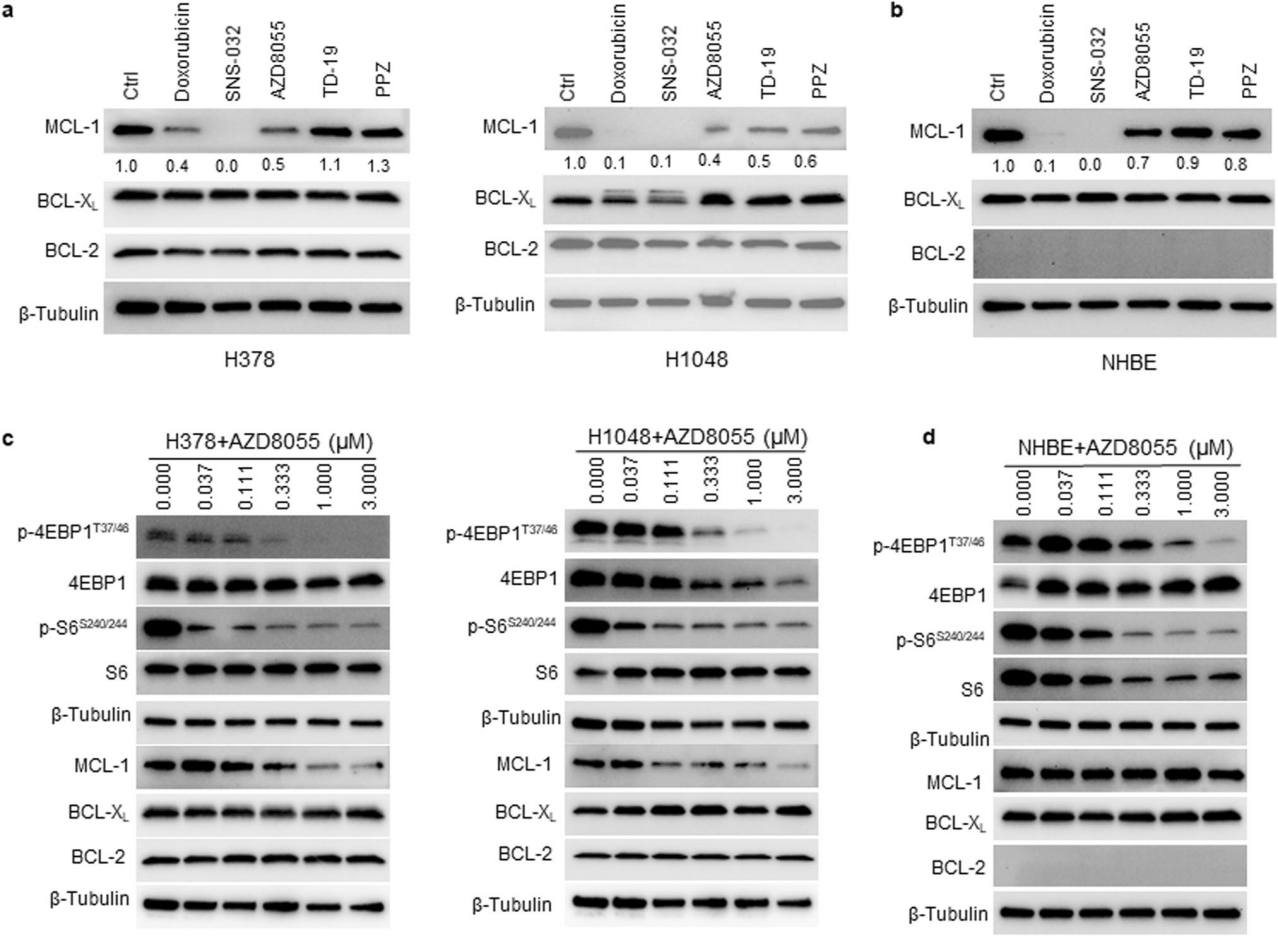
mTOR inhibitor AZD8055 selectively suppresses MCL-1 expression in tumor cells. **a**, **b** Immunoblot analyses of MCL-1, BCL-X_L,_ and BCL-2 in SCLC H378 and H1048 cell lines (**a**) and NHBE cells (**b**) after they were treated with indicated agents (1 µM each) for 24 h. Normalized densitometric values for MCL-1 blots are shown underneath. **c**, **d** Immunoblot analysis of mTOR substrates (p-4EBP1 and p-S6), MCL-1, BCL-X_L_, and BCL-2 after 24 h treatment with indicated concentrations of AZD8055 in H378 and H1048 SCLC cell lines (**c**) and NHBE cells (**d**). The uncropped immunoblot images related to this figure are provided in the “Supplemental Material” file.

Next, we sought to identify a strategy to selectively suppress MCL-1 expression in SCLC cells by rationally targeting certain tumorigenic proteins/pathways. We screened several clinical-stage compounds and FDA-approved drugs including doxorubicin (a topoisomerase II inhibitor and a commonly used chemotherapeutic), SNS-032 (a promiscuous cyclin-dependent kinase [CDK] inhibitor that primarily targets CDK9), AZD8055 (an ATP-competitive catalytic mTORC1/2 inhibitor), TD-19 (a cancerous inhibitor of protein phosphatase 2A inhibitor) and piperazine (a protein phosphatase 2A [PP2A] activator). All of these therapeutic agents have previously been shown to suppress MCL-1 expression through different mechanisms [10, 26–29]. Here, we aimed to determine whether any of these agents could selectively suppress MCL-1 expression in tumor cells while having minimal/no significant effect on the expression of MCL-1 in normal cells. In agreement with previous reports, doxorubicin, SNS-032 and AZD8055 caused significant suppression of MCL-1 expression in all tested SCLC cell lines (Fig. 3a; Supplementary Fig. 1b). Among these inhibitors tested, only AZD8055 showed minimal effect on the expression of MCL-1 in normal cells (Fig. 3b; Supplementary Fig. 1c). None of these agents, except SNS-032, significantly altered BCL-X_L_ or BCL-2 levels in both tumor cells as well as normal cells. Further, we analyzed the dose-dependent effect of AZD8055 on the expression of MCL-1, BCL-X_L,_ and BCL-2 in H378 and H1048 SCLC and NHBE normal cells. AZD8055 caused dose-dependent suppression of MCL-1 expression in tumor cells without any observable effect in normal cells. As expected, AZD8055 had no observable effects on the expression of BCL-X_L_ and BCL-2 in all the tested cell lines. Of note, BCL-2 expression was not detected in NHBE cells *via* immunoblotting. Also, AZD8055 dose-dependently inhibited activation of the mTOR downstream targets, i.e., p-4EBP1 and p-S6, in both SCLC and NHBE cells indicating a desired on-target effect (Fig. 3c, d).

**Fig. 4:**
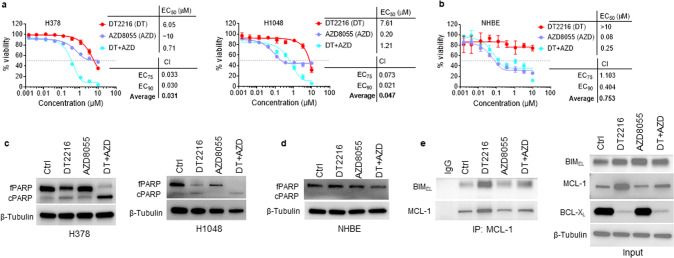
DT2216+AZD8055 combination synergistically kills BCL-X_L_/MCL-1 co-dependent SCLC cells through disruption of MCL-1/BIM interaction. **a**, **b** Viability of H378 and H1048 SCLC (**a**) and NHBE (**b**) cells after they were treated with increasing concentrations of DT2216 (DT) or AZD8055 (AZD) or their combination (1:1 ratio) for 6 days. EC_50_ and CI values are shown. The data presented in **a** and **b** are mean ± SD from a representative experiment (*n* = 3 replicate cell cultures). Similar results were obtained in two additional independent experiments. **c**, **d** Immunoblot analyses of full-length PARP (fPARP) and cleaved PARP (cPARP) in SCLC H378, H1048 (**c**), and NHBE (**d**) cell lines after they were treated with 1 µM each of DT2216 or AZD8055 or their combination for 24 h. **e** Immunoprecipitation analysis of MCL-1 followed by immunoblotting of BIM and MCL-1 in H1048 cells after they were treated with 1 µM each of DT2216 and/or AZD8055 for 24 h. Immunoblot analyses of BIM, MCL-1, and BCL-X_L_ in input samples are shown on the right. The uncropped immunoblot images related to this figure are provided in the “Supplemental Material” file.

Since AZD8055 was found to selectively suppress MCL-1 expression in SCLC cells, we next evaluated it in combination with DT2216 on the viability of BCL-X_L_/MCL-1 co-dependent SCLC cells as well as normal cells. Out of 11 SCLC cell lines that were resistant to BCL-X_L_ inhibitor (A1155463) alone in Fig. 1b, we selected the five most resistant cell lines to test against the combination of BCL-X_L_ inhibitor and MCL-1 inhibitor. Out of these five, three cell lines (H378, H1048, and DMS53) were found to be sensitive to dual BCL-X_L_ and MCL-1 inhibition. Further, we used the two most sensitive (H378 and H1048) cell lines to test the combination of DT2216 and AZD8055. The DT2216 + AZD8055 combination was found to synergistically inhibit the viability of H378 and H1048 SCLC cells, but not normal NHBE and CCD-18Co cells, as assessed by CI values at EC_75_ (concentration with 75% viability loss) and EC_90_ (concentration with 90% viability loss) using Chou-Talalay method [30] (Fig. 4a, b; Supplementary Fig. 1d). Notably, AZD8055 treatment alone led to partial inhibition of H1048, NHBE, and CCD-18Co cell viability which mainly attributed to its antiproliferative effects. Importantly, the combination of DT2216 and AZD8055 caused a complete loss of viability of tumor cells, but not normal cells. Therefore, the combination effects were synergetic in tumor cells, but not in normal cells, as indicated by the CI values. DT2216 treatment induced a dose-dependent BCL-X_L_ degradation in H1048 cells as expected (Supplementary Fig. 2). Since 1 µM of DT2216 was found to completely deplete BCL-X_L_, we selected this concentration for further in vitro experiments. In addition, we found that the combination of DT2216 and AZD8055 was more effective compared to individual agents in inhibiting H1048 cell survival and inducing apoptosis by performing the long-term clonogenic assay and flow cytometric analysis, respectively (Supplementary Fig. 3a, b). Furthermore, the DT2216 + AZD8055 combination profoundly induced PARP cleavage compared to individual agents in tumor cells, but not in NHBE cells, which again confirmed the tumor cell-selectivity of the combination (Fig. 4c, d).

Unlike AZD8055 which inhibits both mTORC1 and mTORC2, everolimus is a rapamycin derivative and a selective mTORC1 inhibitor. Everolimus is FDA-approved for use as an immunosuppressant and for treating certain tumors. We wondered whether everolimus can also synergistically kill BCL-X_L_/MCL-1 co-dependent SCLC cells when combined with DT2216. Indeed, the combination of everolimus and DT2216 synergistically reduced the viability of H378 and H1048 cells, as well as more effectively induced apoptosis compared to individual agents in H1048 cells (Supplementary Fig. 4a, b). However, unlike AZD8055, everolimus alone was less effective in inhibiting the viability of H1048 cells, which might be because it caused even lesser apoptosis compared to AZD8055 (Supplementary Fig. 3b, 4b). This was accompanied by a significant reduction in MCL-1 expression in H1048 cells after everolimus treatment (Supplementary Fig. 4c). These results suggest that the tumor-specific inhibition of only mTORC1 is sufficient for the suppression of MCL-1 expression. Also, mTOR seems to differentially regulate MCL-1 in tumor and normal cells, therefore we could not see MCL-1 downregulation even after efficient mTOR inhibition in NHBE cells.

To gain further insights into the mechanism of synergistic activity of DT2216 + AZD8055, we performed a co-immunoprecipitation (co-IP) analysis of MCL-1 and BIM because BIM acts as a BH3-only pro-apoptotic protein that can trigger apoptosis by activating the apoptotic effectors BAX and BAK and the sequestration of BIM by MCL-1 or BCL-X_L_ inhibits apoptosis [31]. Since BIM is known to bind both BCL-X_L_ and MCL-1, we hypothesized that the degradation of BCL-X_L_ with DT2216 may lead to an increased association of BIM with MCL-1, and co-treatment with DT2216 + AZD8055 may disrupt this interaction. In line with our hypothesis, we found that DT2216 treatment increases the BIM/MCL-1 association which was disrupted when the cells were treated with the DT2216 + AZD8055 combination (Fig. 4e). Interestingly, increased BIM binding seems to stabilize MCL-1 upon DT2216 treatment, and the MCL-1 levels are suppressed in the combination-treated cells. Since BCL-X_L_ inhibition has been shown to increase MCL-1 levels in some cancer cell lines as a compensatory mechanism [32]. So, the increased MCL-1 levels upon DT2216 treatment may be due to either BIM-induced stabilization and/or a compensatory mechanism that needs further exploration.

### DT2216+AZD8055 combination synergistically inhibits tumor growth in SCLC xenograft and PDX models

**Fig. 5:**
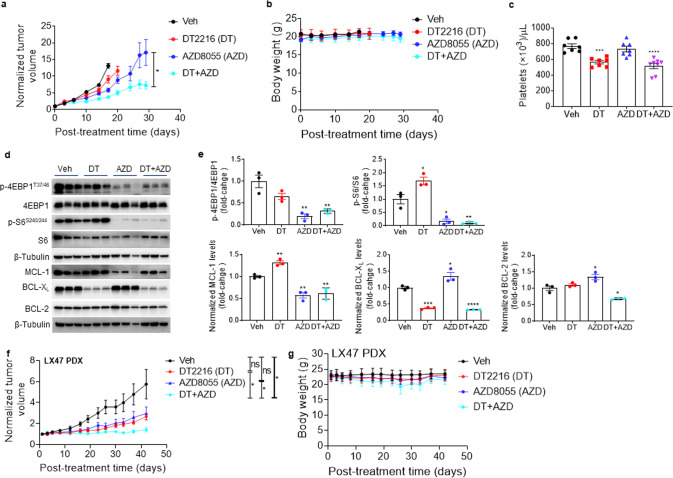
The combination of DT2216 and AZD8055 has stronger antitumor responses in H1048 xenograft and SCLC PDX models. **a**, **b** Tumor volume (**a**) and mouse body weight (**b**) change in H1048 xenografts after treatment with vehicle (Veh), DT2216 (DT, 15 mg/kg/q4d, i.p.), AZD8055 (AZD, 16 mg/kg/5x week, p.o.) or a combination of DT and AZD. Data are presented as mean ± SEM (*n* = 5 mice in Veh and single-treatment groups, and six mice in combination group at the start of treatment). **c** Platelet counts 24 h after the first dose of DT2216 (DT, 15 mg/kg/q4d, i.p.) and/or AZD8055 (AZD, 16 mg/kg/5x week, p.o.) in H1048 xenograft mice from a separate experiment (*n* = 7 mice in Veh and single-treatment groups, and eight mice in combination group). **d** Immunoblot analysis of indicated proteins in H1048 xenograft tumors at the end of treatments as in **a** (*n* = 3 mice per group). **e** Densitometry of immunoblots shown in panel **d** (mean ± SEM). **f**, **g** Tumor volume (**f**) and mouse body weight (**g**) changes in LX47 PDX tumors after treatment with veh, DT2216 (DT, 15 mg/kg/q4d, i.p.) or AZD8055 (AZD, 16 mg/kg/5x week, p.o.) or a combination of DT and AZD. Data are presented as mean ± SEM (*n* = 8 mice in single-treatment groups, and nine mice in Veh and combination groups at the start of treatment). Statistical significance in **a**, **c**, **e**, and **f** was determined by a two-sided unpaired Student’s *t* test. *p < 0.05, **p < 0.01, ***p < 0.001, ****p < 0.0001; ns, not significant. The uncropped immunoblot images related to this figure are provided in the “Supplemental Material” file.

To further assess the combined mTOR inhibition and BCL-X_L_ degradation as a potential therapeutic strategy for BCL-X_L_ and MCL-1 co-dependent SCLC, we first investigated the efficacy of DT2216 + AZD8055 combination using the H1048 xenograft tumor model. In agreement with the in vitro results, DT2216 alone had no effect on H1048 tumor growth. Although AZD8055 alone showed some efficacy, the effect was not significant. On the other hand, the combination of DT2216 and AZD8055 led to significant inhibition of tumor growth (Fig. 5a). More importantly, the combination treatment appeared to be safe as indicated by no significant change in mouse body weights after the treatment (Fig. 5b). In addition, we did not observe any gross organ toxicities upon necropsy in mice treated with DT2216 + AZD8055. Furthermore, the combination caused a similar reduction (25–30%) in platelets as DT2216 alone, where platelet counts remained well above 2 × 10^5^ per µL of blood (Fig. 5c). Notably, platelet counts above 2 × 10^5^ per µL of blood in mice (equivalent to 5 × 10^4^ per µL in humans) are not associated with a significant risk of hemorrhage and considered to be clinically safe [16]. Therefore, a 25–30% reduction in total platelet count may likely not be of any clinical concern. Next, we confirmed that DT2216 and AZD8055 led to a significant reduction in BCL-X_L_ and MCL-1 levels, respectively, which was associated with significant inhibition of mTOR substrates (p-4EBP1 and p-S6) upon AZD8055 treatment in H1048 tumor lysates. Interestingly, the combination also caused a significant decrease in BCL-2 protein levels in these tumor samples (Fig. 5d, e).

Since the PDX models better recapitulate human cancers in vivo, we next evaluated the effect of the combination of DT2216 + AZD8055 in the LX47-SCLC PDX model. We found that the combination significantly inhibited tumor growth in this PDX model of SCLC, whereas single agents failed to have significant effects(Fig. 5f). Again, we did not observe any significant body weight loss with the combination treatment in these mice (Fig. 5g).

### DT2216+AZD8055 combination inhibits lung tumor growth and increases mice survival in the *Rb1/p53/p130* GEM model

**Fig. 6:**
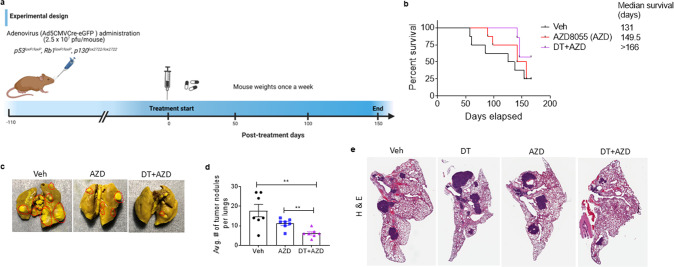
The combination of DT2216 and AZD8055 has stronger antitumor responses as compared to individual agents in a GEM model of SCLC. **a** Experimental design of *Rb1/p53/p130* GEM model. Mice were administered with 2.5 × 10^7^ pfu/mouse of Ad5CMVCre-eGFP through nasal inhalation. Treatment was started 110 days post-infection and continued until day 150. Specifically, the mice were treated with vehicle (veh), AZD8055 (AZD, 16 mg/kg/5x week, p.o.) and AZD (16 mg/kg/5x week, p.o.) + DT2216 (DT, 15 mg/kg/q4d, i.p.) (*n* = 8, 8 and 7 mice in veh, AZD and combo groups, respectively). The figure was adapted from BioRender. **b** Kaplan–Meier survival analysis of mice as treated in **a**. The median survival time is shown on the right. At the end of the experiment (day 166), 2, 2, and 4 mice were alive in veh, AZD and DT + AZD groups, respectively. **c** Bouin’s-stained represented images of excised lungs from veh, AZD, and DT + AZD groups. The tumor nodules in the lungs are circled. As shown, the tumor nodules were considerably smaller in the lungs of combination-treated mice as compared to veh or AZD-treated mice. **d** The Number of tumor nodules was counted on both dorsal and ventral sides of the lungs and the average number of tumor nodules per mouse lungs in each group is shown (mean ± SEM, *n* = 7). Statistical significance was determined by a two-sided unpaired Student’s *t* test. **p < 0.01. **e** Hematoxylin and eosin (H&E) staining of representative lung sections in veh, DT, AZD, and DT + AZD groups from a separate experiment in which the mice were treated with veh, DT (15 mg/kg/q4d, i.p.), AZD (16 mg/kg/5x week, p.o.), and a combination of DT and AZD. The mice were euthanized 24 h after the last treatment.

Next, we used the conditional-mutant *Rb1/p53/p130* mouse model of SCLC to test the efficacy of the DT2216 + AZD8055 combination. In these mice, *Rb1, Tp53*, and *p130* tumor suppressor genes have flanking lox sequences that are under the control of Cre recombinase. These genes are deleted upon administration of Adenovirus (Ad5CMVCre-eGFP), specifically in the lung epithelial cells, and lead to tumor formation in the lungs [33, 34]. Since the *Rb1/p53/p130* mouse tumors closely resemble human SCLC, it is a suitable preclinical mouse model to evaluate newer therapeutics against SCLC. We determined a mean tumor onset time of 110 days after administration of adenovirus titer of 2.5 × 10^7^ pfu/mouse to induce the formation of small visible tumor nodules in the mouse lungs. Therefore, we started treating the mice after 110 days with DT2216 and/or AZD8055 for up to 150 days (Fig. 6a). We did not include the DT2216 alone group because DT2216 was not found to have any significant effect on tumor growth when used alone in this model in a previous study as shown in Fig. 6e. Mice were euthanized, and the survival was recorded at the humane endpoint when the body condition score of animals declined [35]. At the end of the experiment i.e, 150 days after treatment, 2/8 (25%), 2/8 (25%) and 4/7 (~57%) mice were alive in the vehicle, AZD8055, and combination-treatment groups, respectively. The median survival time was 131, 149.5, and >166 days in the vehicle, AZD8055, and the combination-treatment group, respectively (Fig. 6b). We excised the lungs from these mice and observed that the combination-treated lungs had much smaller tumor nodules as compared to vehicle or AZD8055 treatments (Fig. 6c). Moreover, the number of total tumor nodules per lungs was also significantly reduced in the combination-treated mice (Fig. 6d). The histopathological staining in the lungs from a separate experiment further evidenced the reduction in tumor burden in the combination-treated mice (Fig. 6e). We did not see any significant changes in the lungs, spleen, liver, or kidney weights after combination treatment (Supplementary Fig. 5a). The reductions in spleen weight in AZD-treated mice might be attributable to the immunosuppressive effect of mTOR inhibitors. DT2216 might be able to reduce the immunosuppressive effect of mTOR inhibition in part *via* depleting tumor-infiltrating regulatory T cells (TI-Tregs) as shown in our recent study [36]. Depletion of TI-Tregs by DT2216 may stimulate antitumor immunity and attenuate mTOR inhibition-induced immunosuppression and spleen size reduction. Furthermore, we did not see any clinically significant reduction in platelets and other blood cell counts in the treatment cohorts (Supplementary Fig. 5b, c). Overall, these results suggest that the DT2216 + AZ8055 combination may be a promising therapeutic strategy, particularly against BCL-X_L_/MCL-1 co-dependent SCLC, and therefore this combination could potentially be tested in clinical trials in the future.

## Discussion

SCLC shows high inter- and intra-tumoral heterogeneity which is primarily responsible for the therapeutic resistance [6–9]. This heterogeneity is in part attributed to an aberrant expression of anti-apoptotic BCL-2 family proteins which are crucial for the survival of SCLC cells [10–12]. In the present study, we first established the survival dependence of SCLC cell lines on different BCL-2 family anti-apoptotic proteins using BH3 mimetic screening. We found that most SCLC cell lines are dependent on BCL-X_L_ for their survival and are highly sensitive to a BCL-X_L_ inhibitor or our recently developed BCL-X_L_ PROTAC DT2216 (now a clinical candidate in the phase-I trial) [16, 17]. On the other hand, some SCLC cell lines were found to be dependent on multiple co-expressed BCL-2 family proteins such as concurrent BCL-X_L_ and BCL-2, or concurrent BCL-X_L_ and MCL-1. BCL-X_L_/BCL-2 co-dependent cell lines could be targeted with navitoclax, however, the clinical translation of navitoclax is hampered by dose-limiting on-target toxicity of thrombocytopenia [14, 15]. More importantly, BCL-X_L_/MCL-1 co-dependent tumor cells cannot be safely targeted with the currently available inhibitors because the combination of BCL-X_L_ and MCL-1 inhibitors causes severe tissue damage and lethality in mice [22–24]. Therefore, finding a strategy to selectively target BCL-X_L_ and MCL-1 in tumors without causing significant on-target toxicity was the primary goal of our current study for treating an important difficult-to-treat subset of SCLC.

Since systemic MCL-1 inhibition with currently available inhibitors leads to normal tissue toxicity and lethality when combined with a BCL-X_L_ inhibitor/PROTAC, we sought to identify a strategy to selectively suppress MCL-1 expression in SCLC cells. After screening several compounds that target oncogenic pathways known to upregulate MCL-1 [10, 26–29], we identified that AZD8055, a mTORC1/2 ATP-competitive inhibitor, and everolimus, a selective mTOR1 inhibitor, can selectively suppress MCL-1 expression in SCLC cells but not in normal cells. In contrast, other compounds such as doxorubicin and SNS-032 non-specifically downregulated MCL-1 in tumor cells as well as in normal cells. This was correlated with high basal mTOR activation in MCL-1-dependent SCLC cell lines (data not shown). The mTOR activation has been shown to enhance cap-dependent MCL-1 translation, and therefore, inhibition of mTOR suppresses MCL-1 at post-translational level [26, 28, 29, 37]. Of note, the combination of DT2216 with AZD8055 or everolimus synergistically inhibited the viability and induced apoptosis in BCL-X_L_ and MCL-1 co-dependent SCLC cell lines, but not the normal cells from different tissue origins including lungs and colon.

Our further investigation using different in vivo SCLC models including conventional xenografts, PDX, and *Rb1/p53/p130* GEM model also suggests that the combination of DT2216 and AZD8055 can effectively inhibit the SCLC growth in mice. More importantly, the combination appeared to be safe as evidenced by no significant decrease in mouse body weights as well as no clinically significant reduction in different blood cells including platelets, and the absence of any observable tissue pathology. Interestingly, results using the SCLC GEM model, which closely recapitulates genetic heterogeneity found in human SCLC, indicate that the combination of DT2216 + AZD8055 strongly inhibits tumor growth in the lungs and increased the survival of mice. The effectiveness of the combination in the GEM model was encouraging because the tumors in this model have been shown to be highly resistant to cisplatin plus etoposide doublet chemotherapy [26].

These findings have important clinical implications because tumor-specific targeting of BCL-X_L_ and MCL-1 has high therapeutic value. Two prior studies have found that a combination of navitoclax and AZD8055 synergistically kills different tumor cells from SCLC and *BRAF*/*KRAS*-mutated colorectal cancer [26, 28]. Moreover, a study from Dr. Christine Hann’s group has demonstrated synergy between rapamycin and ABT737 (a predecessor compound of navitoclax) against different PDX models of SCLC [38]. In another study, the combined inhibition of BCL-X_L_ and mTOR was found to synergistically induce apoptosis in *PIK3CA*-mutated breast cancer [37]. However, these studies did not aim to comprehensively evaluate tumor-selectivity and on-target toxicities of this combination (especially in mouse models). Given that BCL-X_L_ inhibitors including navitoclax cause thrombocytopenia, a combination of navitoclax with AZD8055 (or another mTOR inhibitor) may not be clinically feasible [14, 15]. In contrast, by combining AZD8055 with platelet-sparing BCL-X_L_ degrader DT2216, we achieved tumor-selective targeting of both MCL-1 and BCL-X_L_, respectively, without significant on-target toxicity both in cell culture and in mice. This approach can also be applied to other tumor types which are co-dependent on MCL-1 and BCL-X_L_ [39, 40]. One limitation of our in vitro BH3 mimetic screening is that it requires live cells, so it cannot be used to stratify patients who will benefit from the combination. In that case, a dynamic BH3 profiling assay would be more useful as demonstrated previously [41, 42].

More recently, SCLC tumors have been classified into four molecular subtypes based on the relative expression of four transcriptional regulators, i.e., ASCL1, NEUROD1, POU2F3, and YAP1 [5, 7]. Each of these molecular subtypes shows distinct therapeutic vulnerabilities [8, 9]. For example, ASCL1-high subtype (classic or neuroendocrine (NE) SCLC) [7] has been shown to be sensitive to BCL-2 inhibition with ABT263 [43]. Interestingly, we observed that most variant-SCLC cells (non-NE consisting of POU2F3 and/or YAP1 subtype) [7] were dependent on the co-expression of BCL-X_L_ and MCL-1. Although these subtypes are not being used to direct treatment decisions for SCLC at present, we expect that POU2F3 and/or YAP1 SCLC subtypes could be better targeted with a combination of DT2216 and an mTOR inhibitor. In this case, immunohistochemical or proteomics profiling of four of these transcriptional regulators could be a better alternative to BH3 profiling for stratifying patients who can specifically benefit from the DT2216 + mTOR inhibitor combination. In the current study, we used AZD8055 as a proof of concept, but it would be suitable to use an FDA-approved mTOR inhibitor (such as everolimus) in combination with DT2216 in clinical trials.

In conclusion, the findings from our in vitro and in vivo studies suggest that the combination of DT2216 + AZD8055 is synergistic and somewhat tumor-selective against the BCL-X_L_/MCL-1 co-dependent subset of SCLC and is tolerable in mice without appreciable on-target toxicity and normal tissue injury. These findings may have a high potential for near-term clinical translation as the combination of DT2216 and an FDA-approved mTOR inhibitor (such as everolimus) can be rapidly evaluated in SCLC patients, given that DT2216 is already in the phase-I clinical trial (Identifier: NCT04886622). This kind of approach can help to effectively treat an important and difficult-to-treat subset of SCLC patients in the near future.

## Materials and methods

### Cell lines and culture

All the SCLC cell lines, except DMS53 and DMS114, were obtained from the original NCI-Navy Medical Oncology source supply [44]. DMS53 and DMS114 SCLC cell lines, CCD-18Co normal colon fibroblasts, and WI-38 lung fibroblasts were purchased from the American Type Culture Collection (ATCC, Manassas, VA). SCLC cell lines were cultured in RPMI-1640 medium (Cat. No. 22400–089, Thermo Fisher, Waltham, MA). CCD-18Co and WI-38 cells were cultured in Dulbecco’s modified Eagle’s medium (DMEM) (Cat. No. 12430-062, Thermo Fisher). The culture media were supplemented with 10% heat-inactivated fetal bovine serum (FBS, Cat. No. S11150H, Atlanta Biologicals, GA), 100 U/mL penicillin, and 100 µg/mL streptomycin (Pen-Strep, Cat. No. 15140122, Thermo Fisher). NHBE cells were purchased from Lonza (Cat. No. CC-2541, Basel, Switzerland), and were cultured in Lonza’s bronchial epithelial cell growth medium (Cat. No. CC-3170) with supplements and growth factors (CC-4175). The stocks of NCI SCLC cell lines that we used were STR profiled by NIH, so the authenticity of the cell lines remained preserved. All cultures were confirmed for Mycoplasma negativity using the MycoAlert Mycoplasma Detection Kit (Cat. No. LT07–318). All the cell lines were maintained in a humidified incubator at 37 °C and 5% CO_2_.

### Chemical compounds

DT2216 was synthesized in Dr. Guangrong Zheng’s laboratory (University of Florida, Gainesville, FL) according to the previously described protocol [16]. AZD8055 (Cat. No. HY-10422) and everolimus (Cat. No. HY-10218) were purchased from MedChemExpress (Monmouth Junction, NJ). A1155463 (Cat. No. S7800), ABT199 (Cat. No. S8048), S63845 (Cat. No. S8383), and ABT263 (Cat. No. S1001) were purchased from SelleckChem (Houston, TX).

### Cell viability assays

The cell viability was measured by MTS assay according to the manufacturer’s protocol (Cat. No. G-111, Promega, Madison, WI) and as described previously [16, 39]. EC_50_ values were determined using GraphPad Prism software (GraphPad Software, La Jolla, CA).

### BH3 mimetic screening

The screening was performed to determine the survival dependence of SCLC cells on different BCL-2 family anti-apoptotic proteins [21]. The cells were treated in 96-well plates with either a specific inhibitor of BCL-2 family anti-apoptotic proteins such as A1155463 (selective BCL-X_L_ inhibitor), venetoclax (selective BCL-2 inhibitor), and S63845 (selective MCL-1 inhibitor), or a non-specific inhibitor such as navitoclax (BCL-X_L_/BCL-2 dual inhibitor). Thereafter, the viability was assessed using the MTS assay as described above.

### Co-immunoprecipitation

Cell pellets were lysed in the Pierce IP lysis buffer (Cat. No. 87787; Thermo Fisher) supplemented with protease and phosphatase inhibitors as described previously [16, 39]. The supernatants were collected and precleared by incubating with 1 µg of mouse anti-IgG (Cat. No. sc-2025; Santa Cruz Biotechnology [SCB], Dallas, TX) and 20 µL of protein A/G-PLUS agarose beads (Cat. No. sc-2003; SCB) for 30 min at 4 °C. The supernatants containing 1 mg of protein were incubated with 2 µg of anti-MCL-1 (Cat. No. sc-12756; SCB) or anti-IgG antibody overnight followed by incubation with 25 µL protein A/G agarose beads for 1–2 h at 4 °C. Thereafter, the immunoprecipitates were collected by centrifugation, washed three times with IP lysis buffer, mixed with 50 µL of Laemmli’s SDS-buffer, denatured and then subjected to immunoblot analysis for BIM and MCL-1. Anti-rabbit HRP-conjugated Fc fragment-specific secondary antibody (Cat. No. 111-035-046, dilution 1:10000, Jackson ImmunoResearch, West Grove, PA) was used to detect immune complexes in immunoblotting.

### Cell line-derived xenograft and PDX studies

All the animal procedures were performed in accordance with the rules of IACUC. CB-17 SCID-beige mice (5–6 weeks old) were purchased from the Charles River Laboratories (Wilmington, MA). NCI-H1048 (H1048) tumor cells at a density of 2.5 × 10^6^ per mouse in 50% Matrigel (Cat. No. 356237, Corning, Corning, NY), and PBS mixture were injected subcutaneously (s.c.) into the right flank region of the mice as described previously [16, 39]. Tumor size was measured twice a week with digital calipers and tumor volume was calculated using the formula (Length × Width^2^ × 0.5). The mice were randomized into different treatment groups when the tumors reached ~150 mm^3^ (*n* = 5–6 mice per group). Mice were treated with vehicle, AZD8055 (16 mg/kg, 5 days a week, p.o.), DT2216 (15 mg/kg, every four days [q4d], i.p.), and a combination of AZD8055 and DT2216. AZD8055 was formulated in 10% (v/v) DMSO, and 90% of 20% (w/v) Captisol (Cat. No. NC0604701, Cydex Pharmaceuticals a Ligand Company, San Diego, CA, USA) in normal saline and DT2216 was formulated in 50% phosal 50 PG, 45% miglyol 810 N and 5% polysorbate 80. Mice were euthanized when they became moribund, or their tumor sizes reached a humane endpoint as per Institutional Animal Care and Use Committee (IACUC) policy. For euthanasia, animals were sacrificed by CO_2_ suffocation followed by cervical dislocation. The tumors were subsequently harvested, lysed, and used for immunoblot analysis.

The PDX experiments were performed at Johns Hopkins University. NOD-*SCID* IL2Rgamma^null^ (NSG) mice aged 5–6 weeks were purchased from the Jackson Laboratory (Stock No. 005557, Bar Harbor, Maine, USA). LX47 SCLC PDX model was established and characterized by Dr. Christine Hann’s lab at the Johns Hopkins University and was propagated in NSG mice as reported previously [38]. The tumors were harvested after they reached 1500 mm^3^ and cut into ~2–3 mm fragments and were implanted s.c. after submerging in Matrigel in additional NSG mice [38]. The animals were randomized into different treatment groups when the tumors reached 100–200`mm^3^ and were treated as described above (*n* = 8–9 mice per group). The tumor volumes were normalized with their baseline readings on day 1 of treatment and are presented as normalized tumor volumes. The number of mice per group for all animal studies was determined based on our own previous experience as these numbers of mice are sufficient to observe significant differences among different groups.

### Tumor induction and treatments in *Rb1/p53/p130* GEM model

The *Rb1*^*loxP/loxP*^ *p53*^*lox/loxP*^ *p130*^*lox2722/lox2722*^ mouse strain was provided by Dr. Maria Zajac-Kaye (University of Florida) [33, 34]. The presence of floxed sequences (loxP and lox2722) was confirmed by PCR as described previously [34]. Tumors were initiated when mice were 6–8 weeks of age by nasal inhalation of adenovirus serotype 5 expressing Cre-recombinase fused to enhancer GFP under a CMV promoter (Ad5CMVCre-eGFP, University of Iowa Vector Core, Cat. No. WC-U of Iowa 1174) at 2.5 × 10^7^ pfu/mouse. Drug treatments were started 110 days after Ad-Cre delivery, which was the timing to be required for the onset of SCLC tumorlets. Mice were treated with vehicle, AZD8055 (16 mg/kg, 5 days a week, p.o.), and a combination of DT2216 (15 mg/kg, q4d, i.p.) and AZD8055 (*n* = 7–8 mice per group). Survival events were scored when the body condition score (as defined by the American Association for Laboratory Animal Science) of animals declined or per absolute survival events [35]. The lungs were excised upon euthanizing the individual mice, stained in Bouins’ solution, and photographed as described previously [45, 46]. The number of visible tumor nodules was counted on the surface of each pair of lungs and is presented as the average number of tumor nodules/lungs.

### Statistical analysis

All the graphs presented in this manuscript were made and statistical analyses were performed using the GraphPad Prism-9 software. A two-sided unpaired Student’s *t* test was used for comparisons between the means of the two groups. *P* < 0.05 was considered to be statistically significant. The combination index (CI) was calculated using Compusyn software (https://www.combosyn.com/) based on the Chou–Talalay method [30]. CI < 1 indicates a synergistic effect, CI = 1 indicates an additive effect and CI > 1 indicates an antagonistic effect. CDI < 0.7 indicates a significant synergistic effect.

## Supplementary information

Supplementary Information

Original Data File

## Data Availability

All the relevant data are available in the main text or the supplementary information. The raw immunoblot images are supplied as “Supplemental Material”.
