# Supplementary material for: Co-targeting BCL-XL and MCL-1 with DT2216 and AZD8055 synergistically inhibit small-cell lung cancer growth without causing on-target toxicities in mice
Source: Cell Death Discov. 2023 Jan 2;9:1. doi: 10.1038/s41420-022-01296-8 (PMC9806104; doi:10.1038/s41420-022-01296-8)

**Figure 3a: H378**

MCL-1


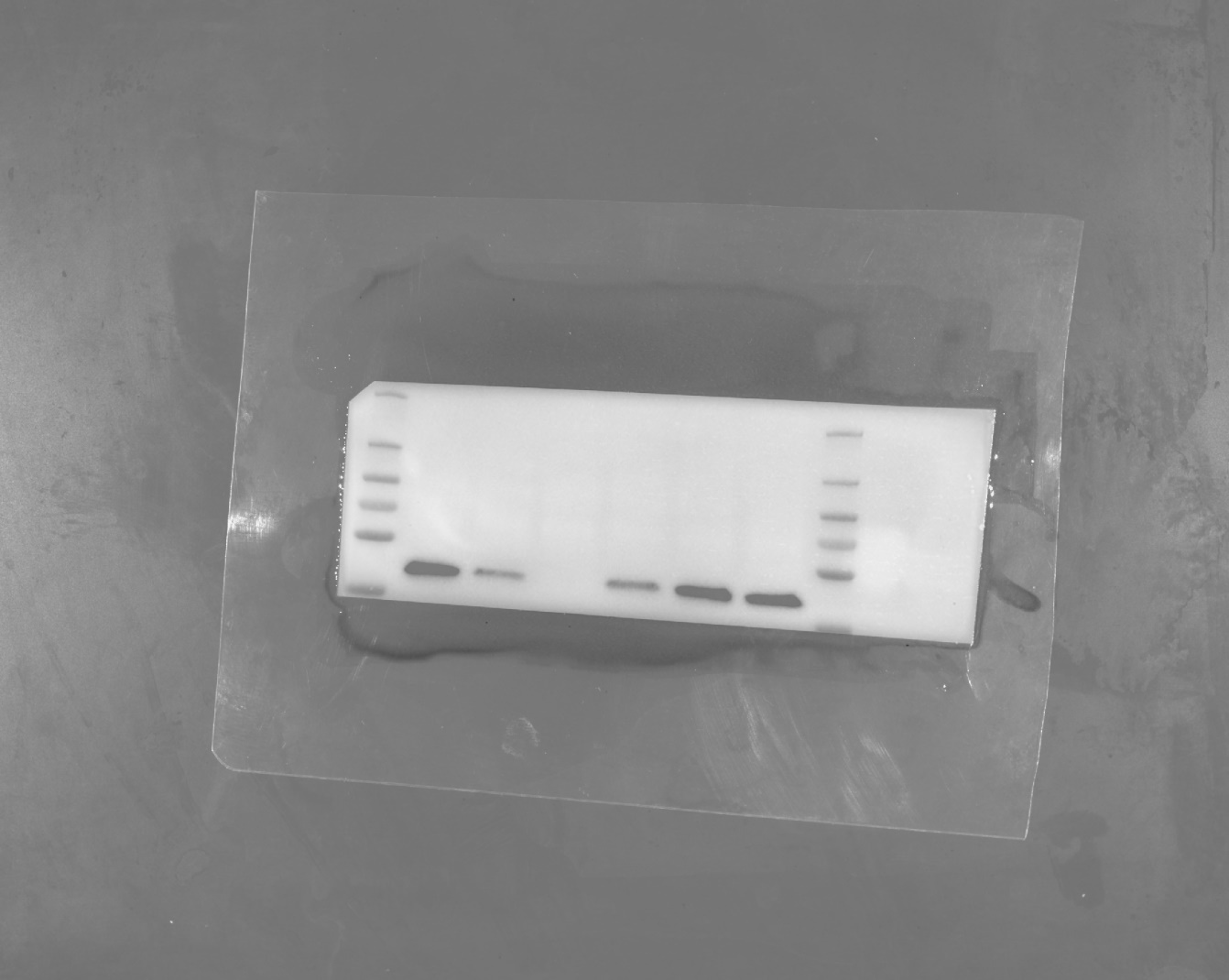


BCL-X_L_


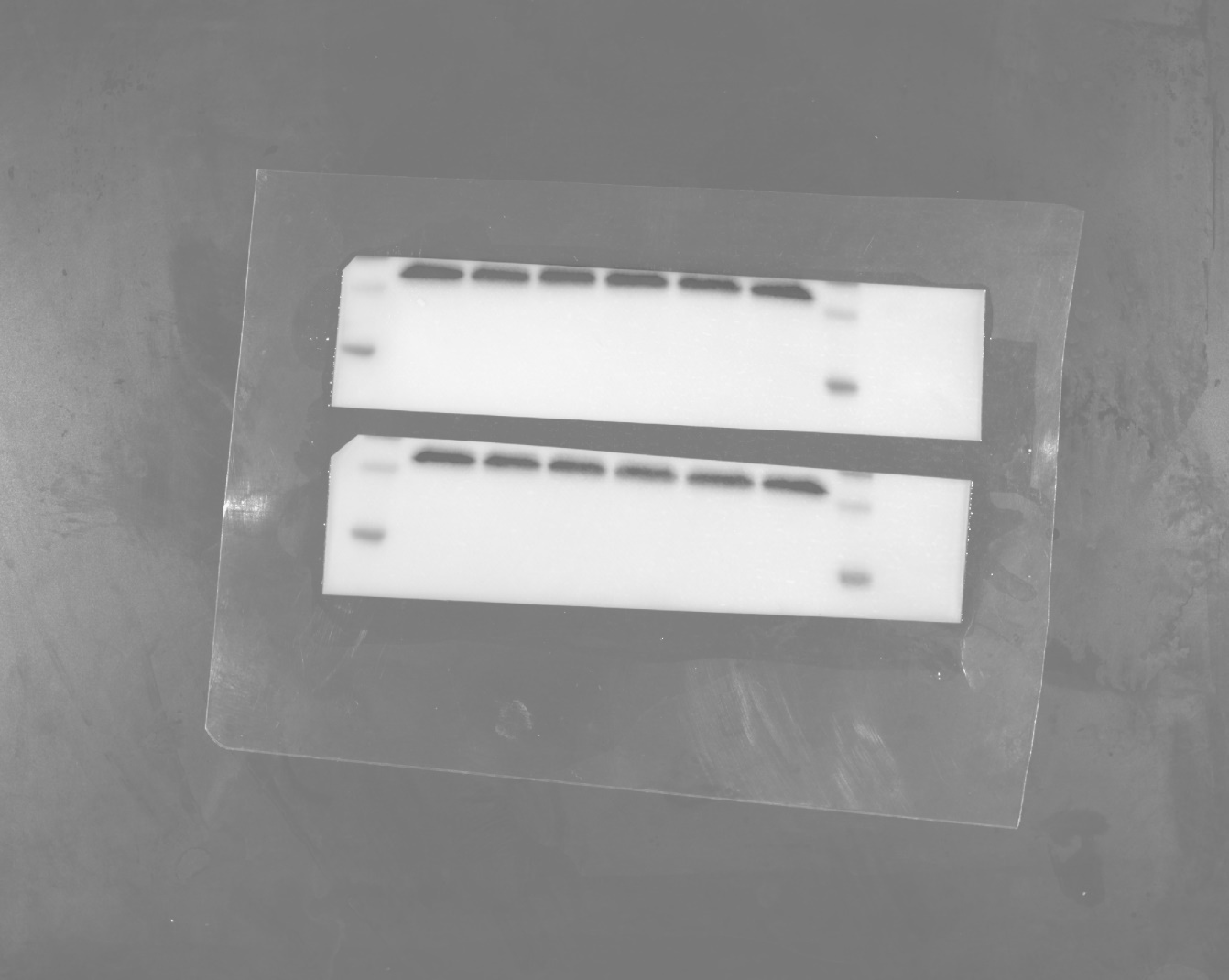


BCL-2


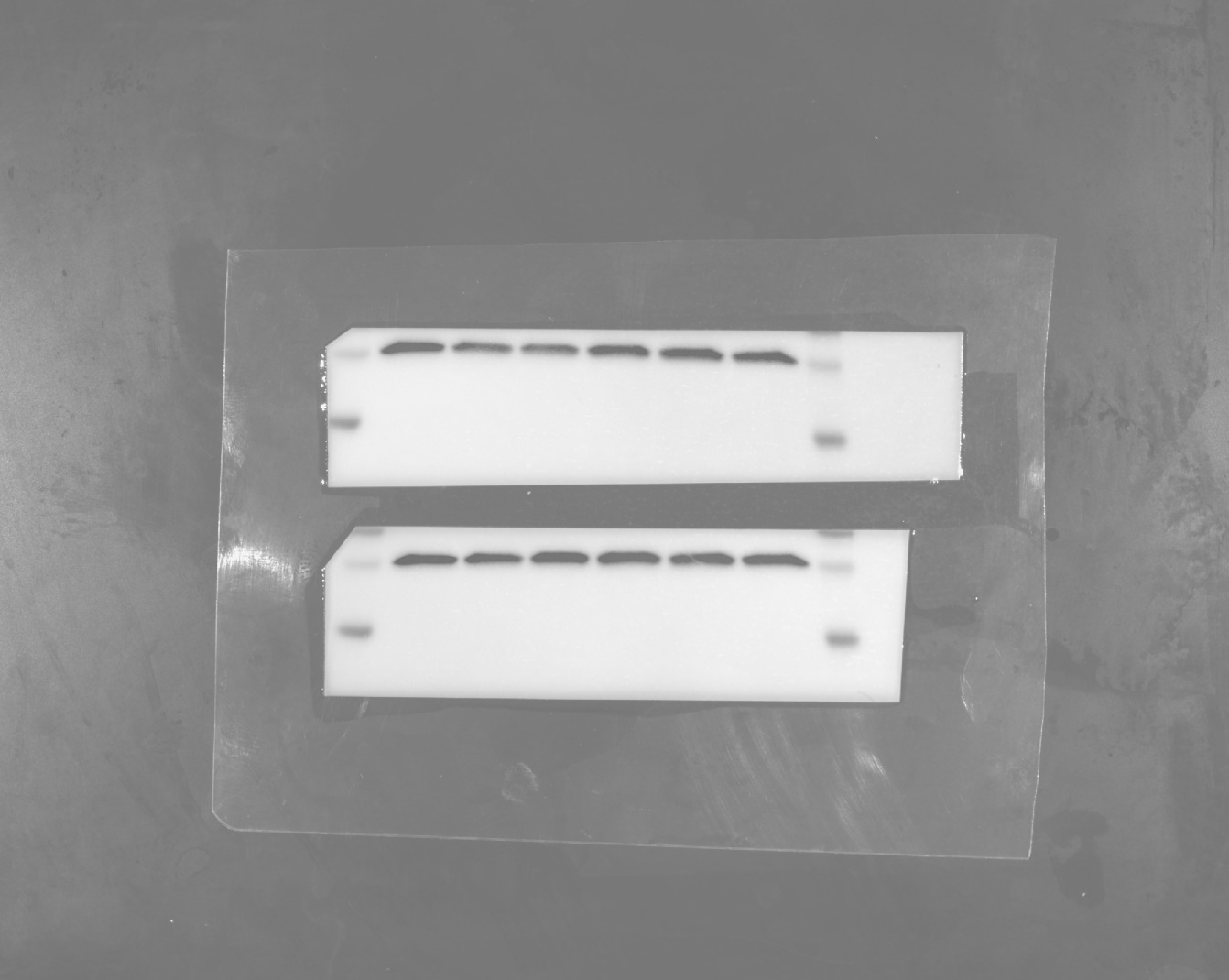


β-Tubulin


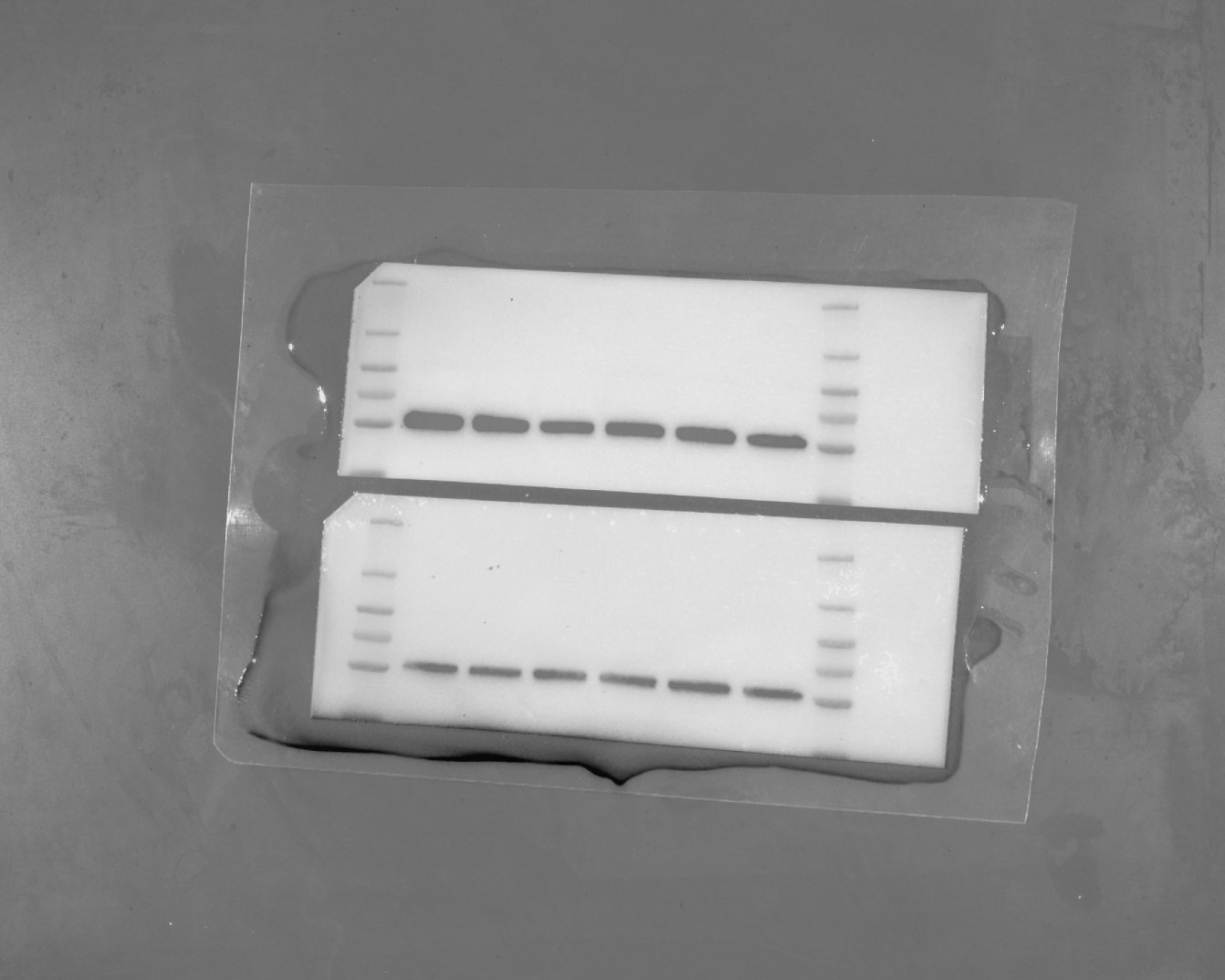


**Figure 3a: H1048**

MCL-1


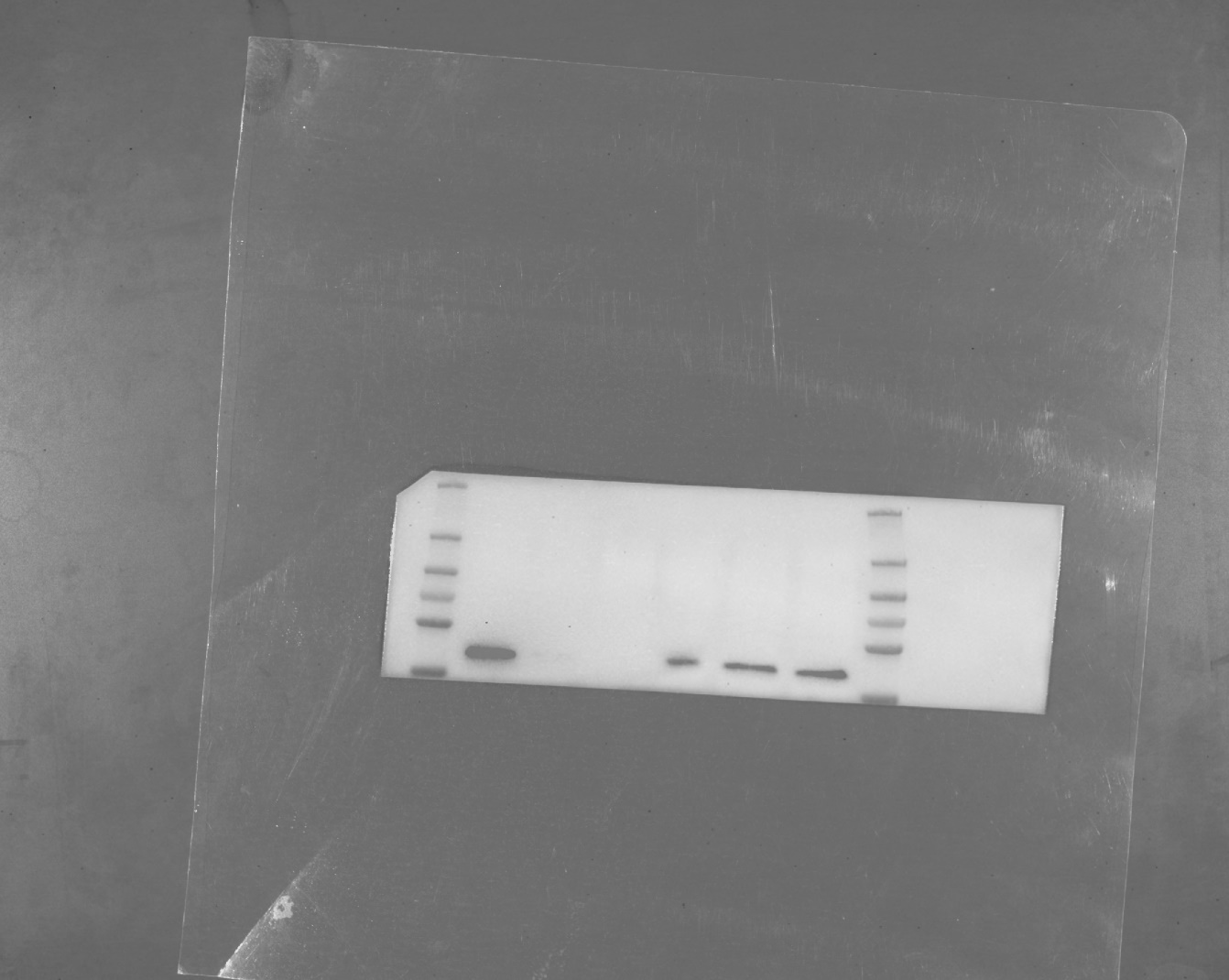


BCL-X_L_


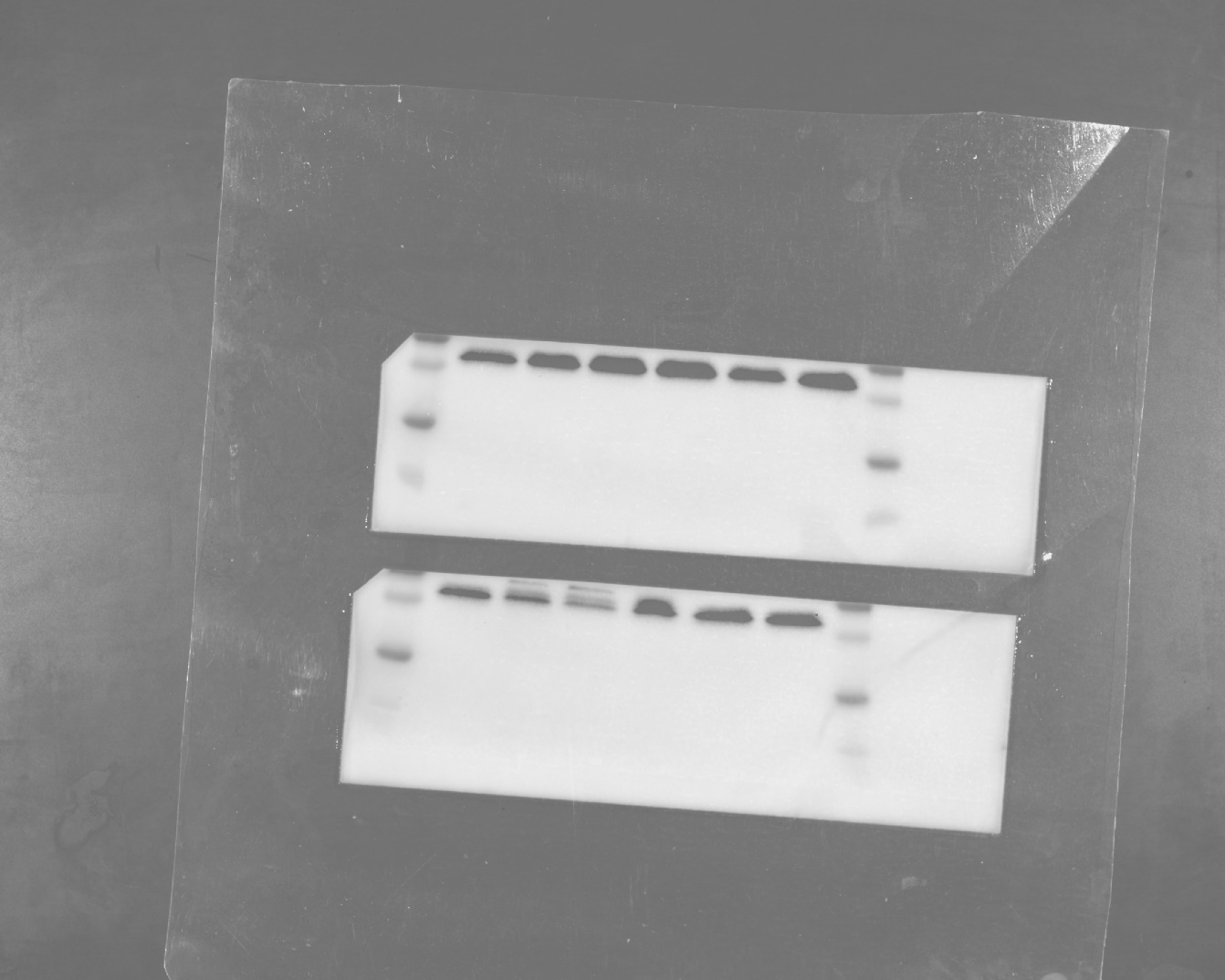


BCL-2


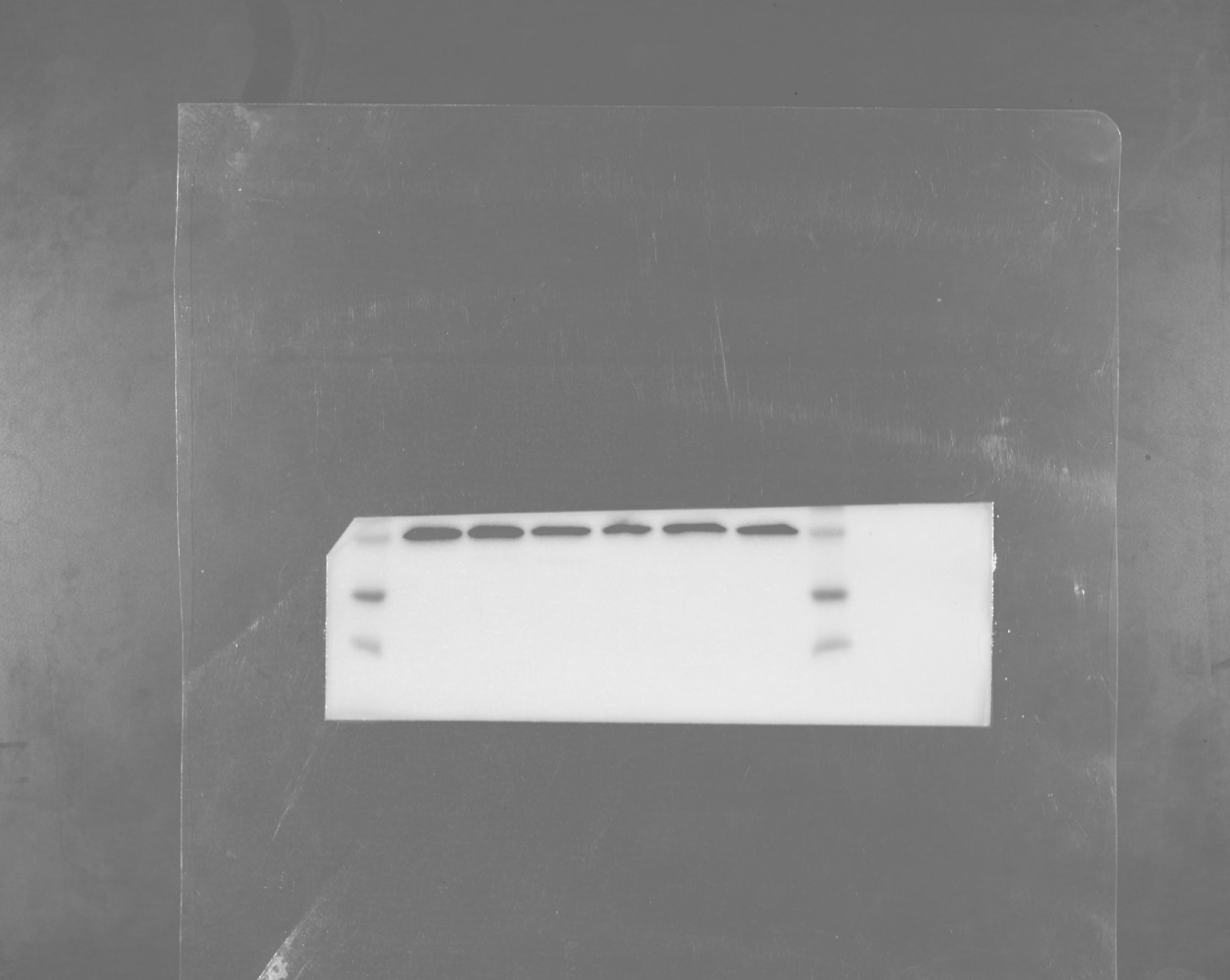


β-Tubulin


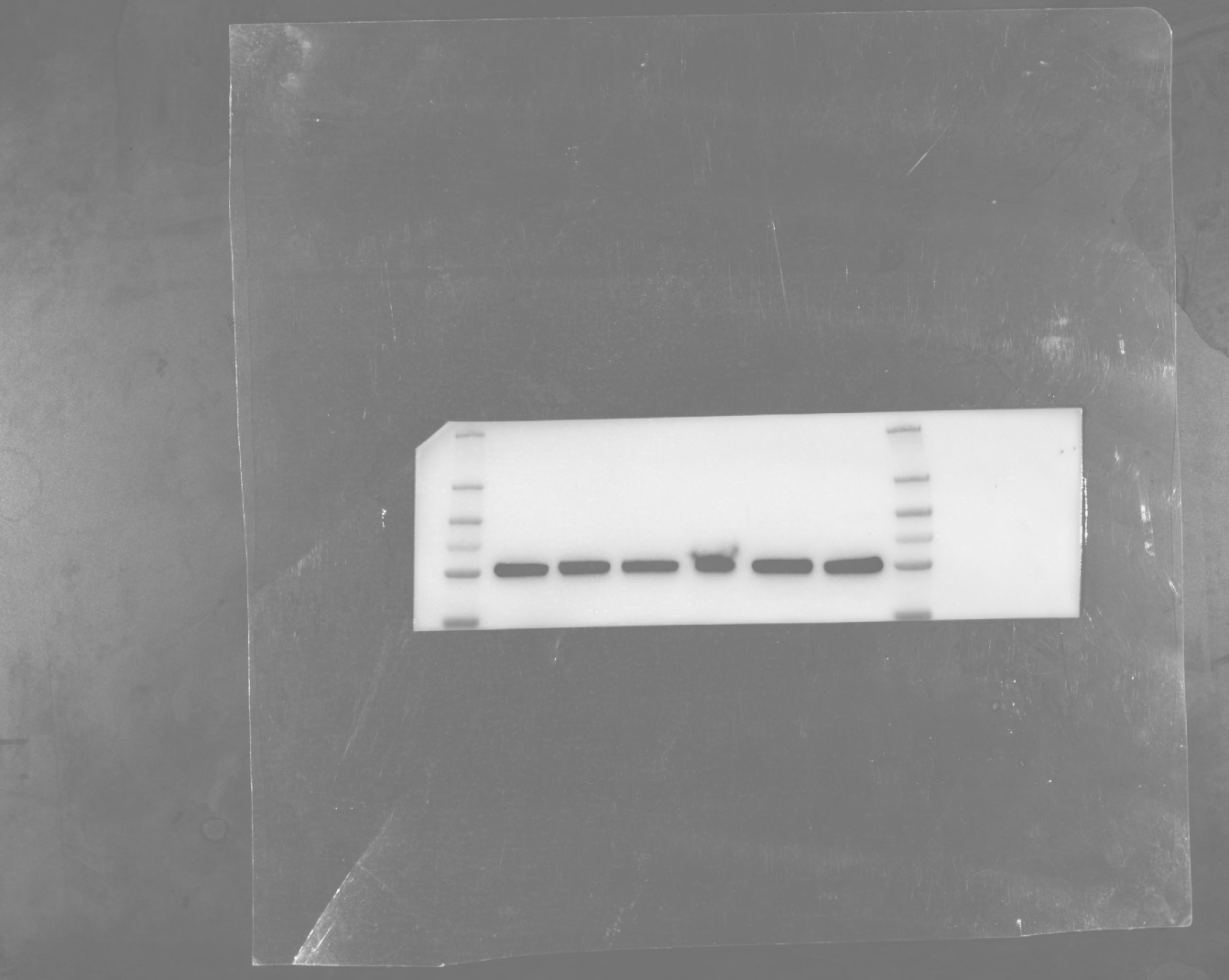


**Figure 3b**

MCL-1


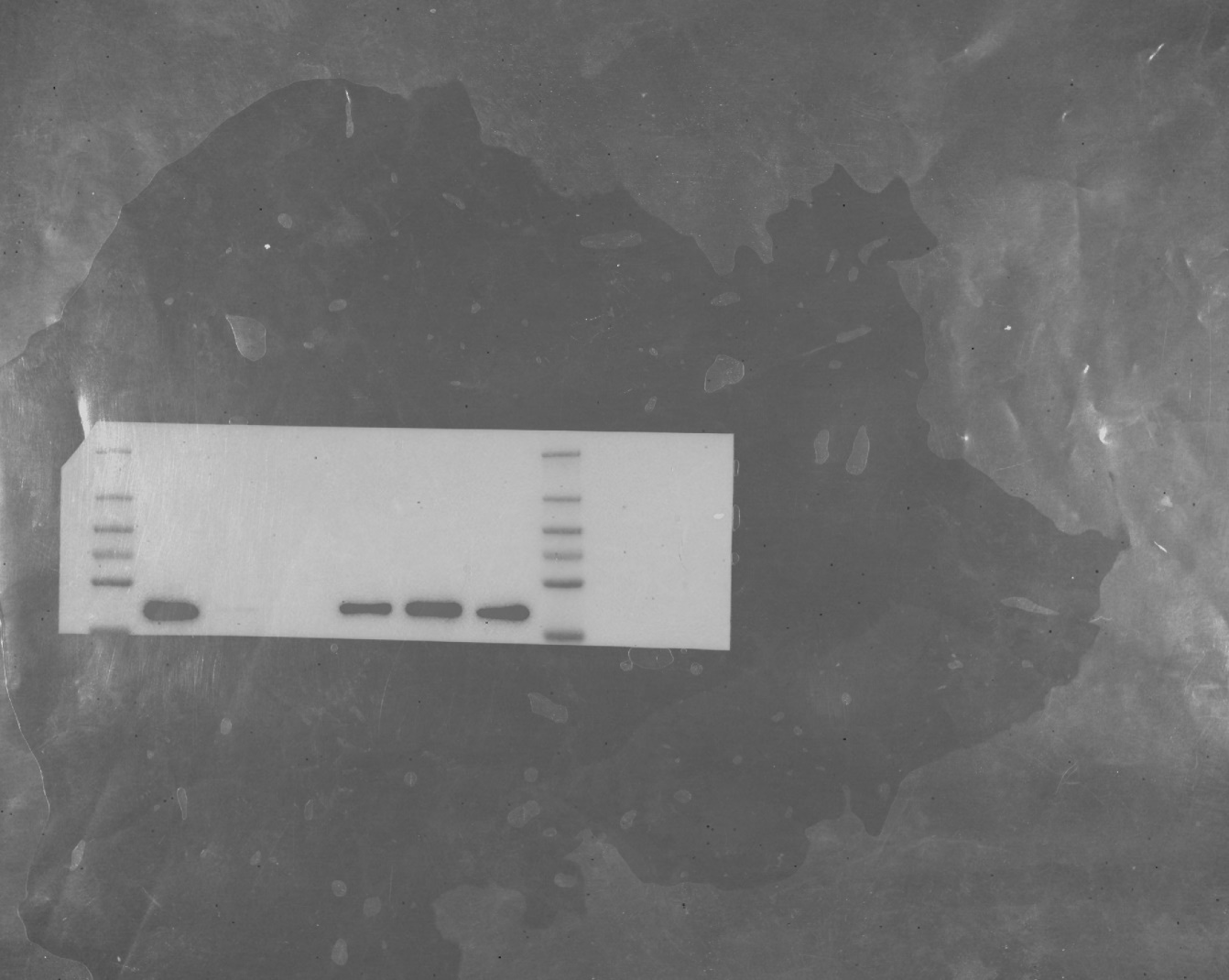


BCL-X_L_


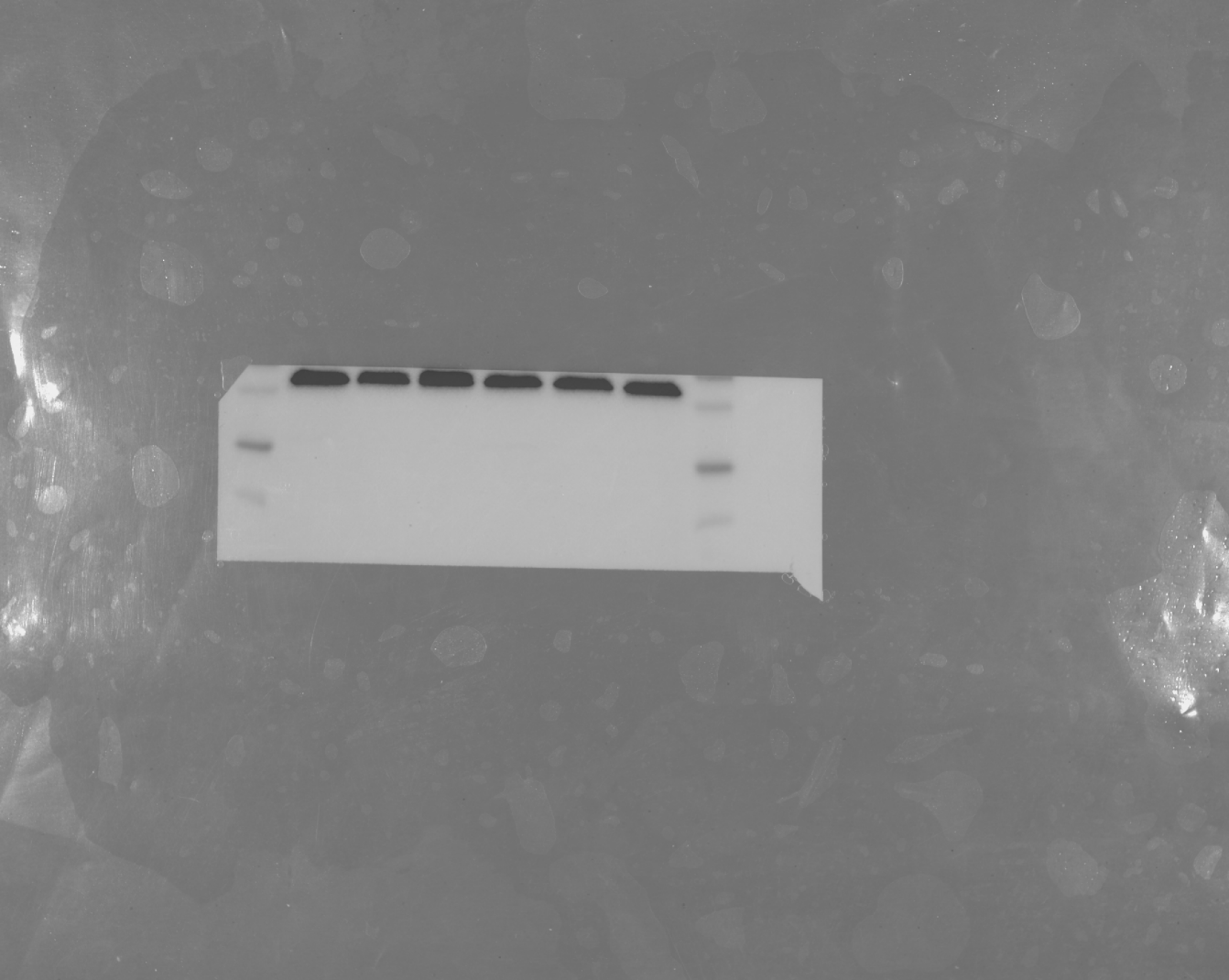


BCL-2


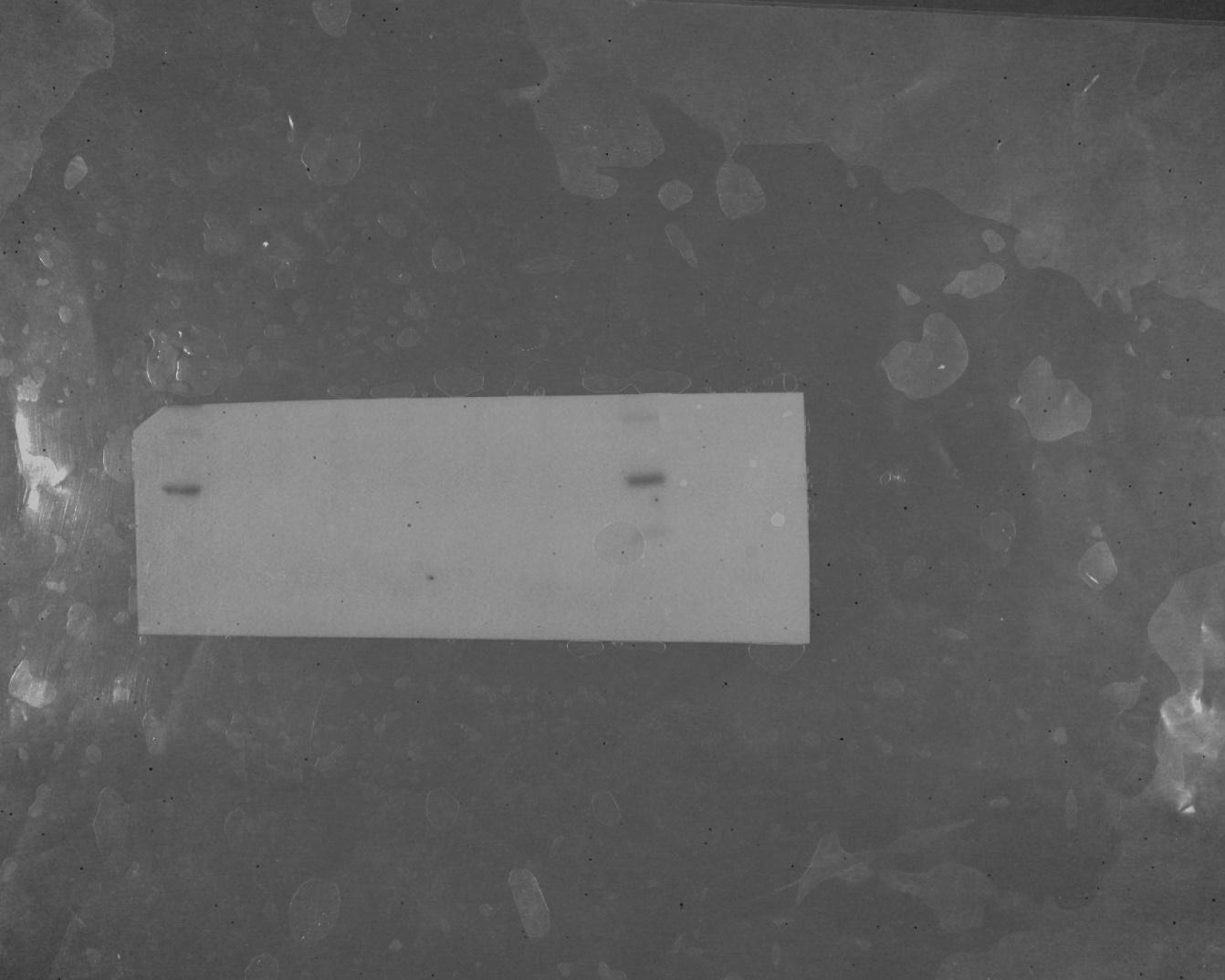


β-Tubulin


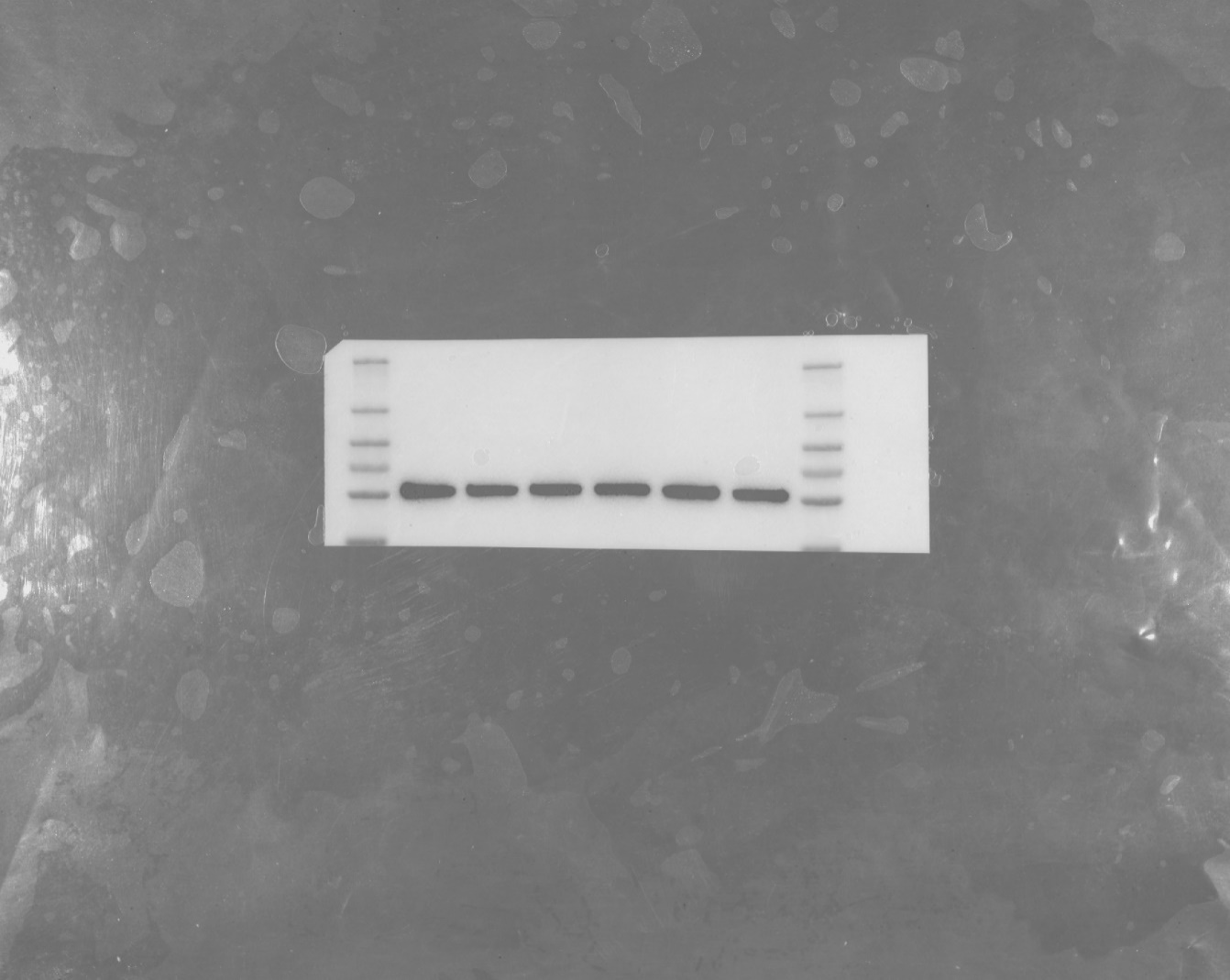


**Figure 3c: H378**

p-4EBP1^T37/46^


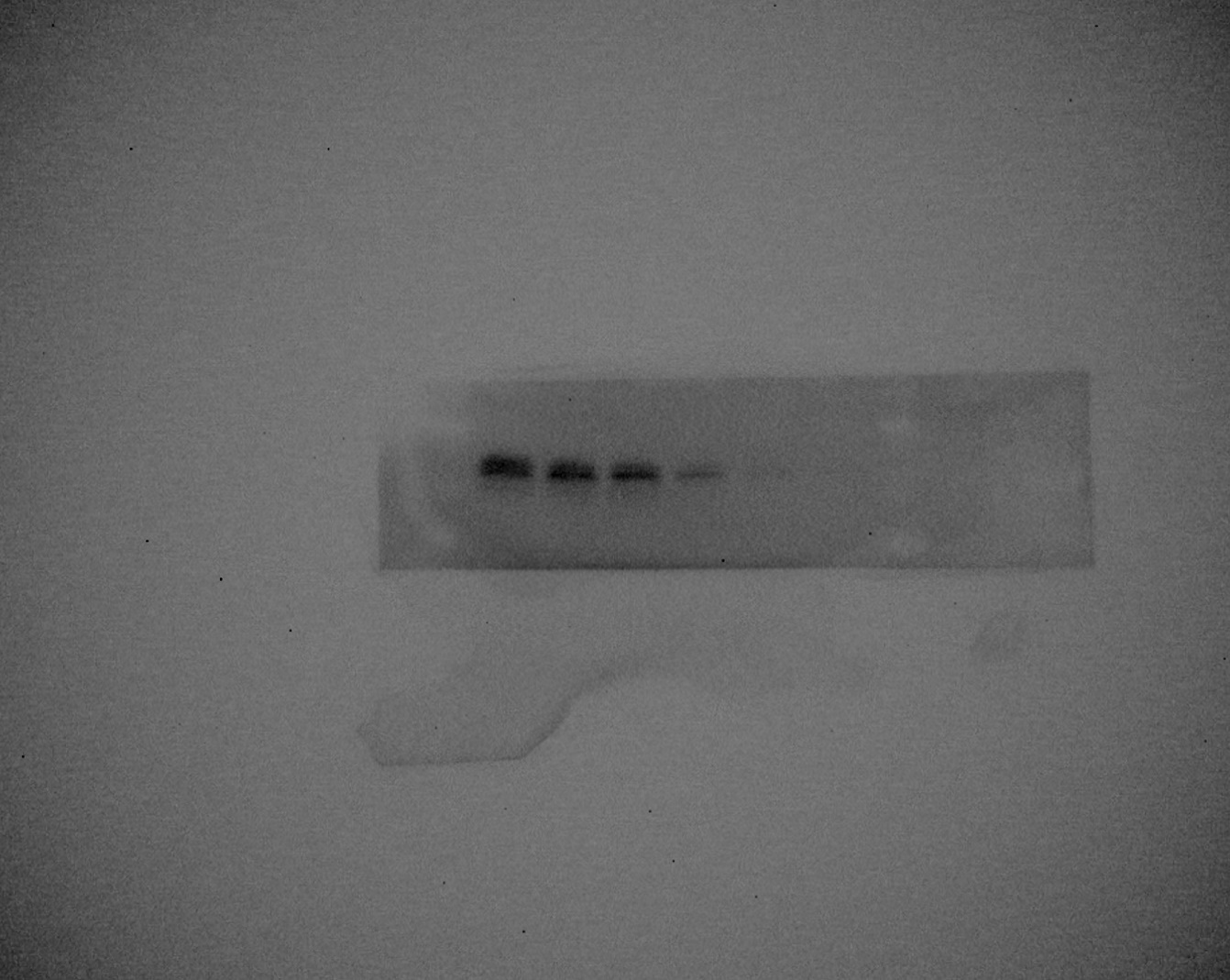


4EBP1


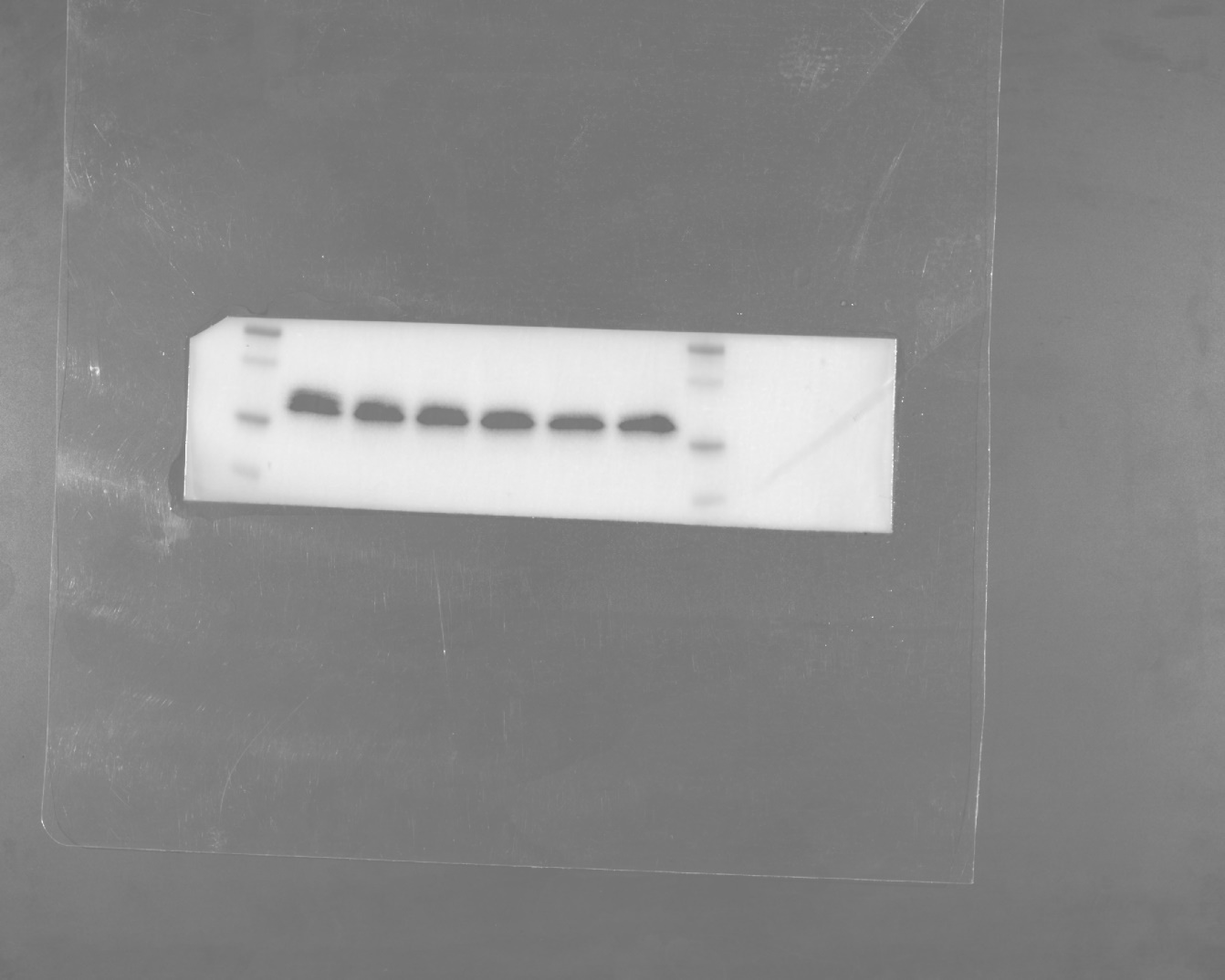


p-S6^S240/244^


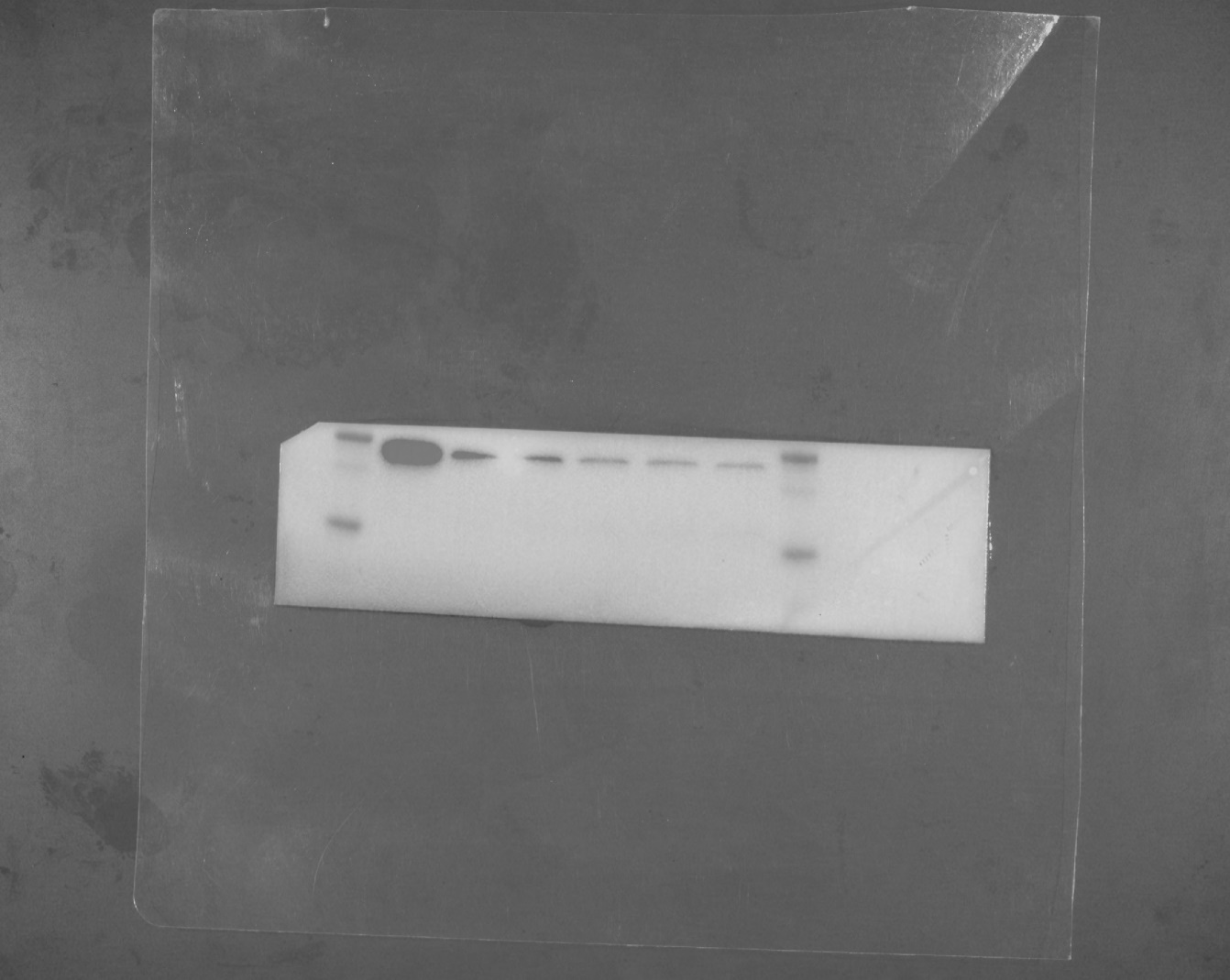


S6


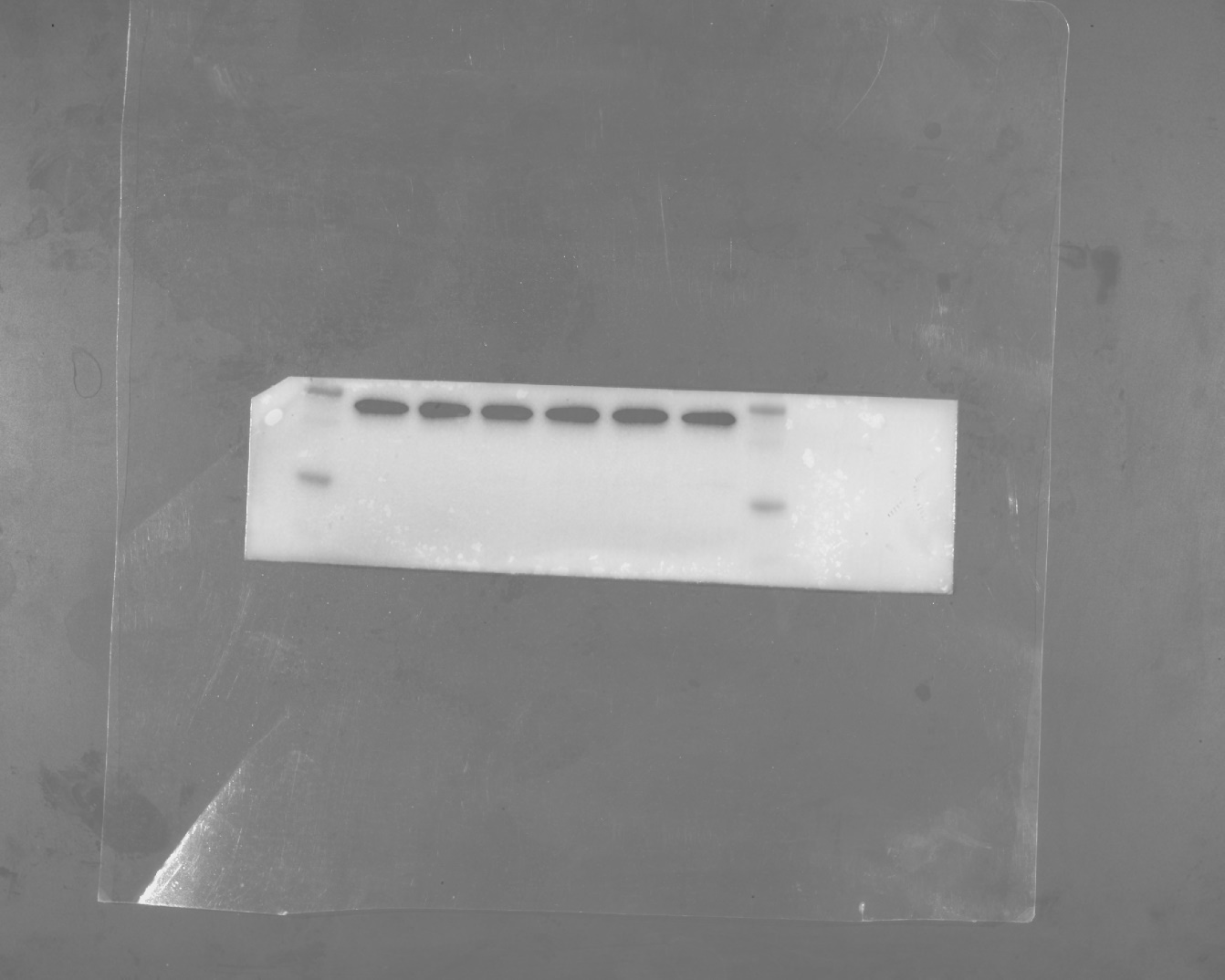


β-Tubulin


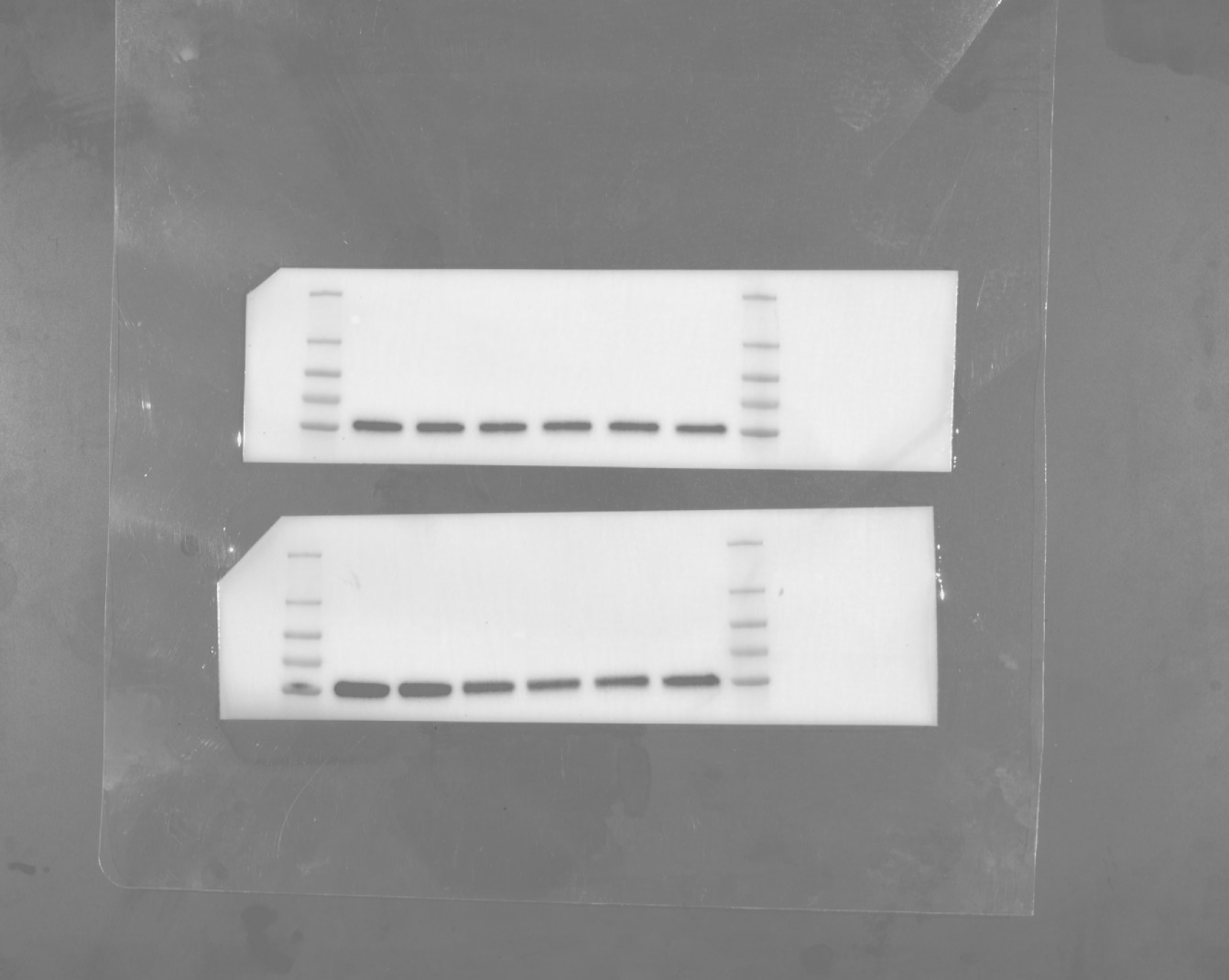


**Figure 3c: H378**

MCL-1


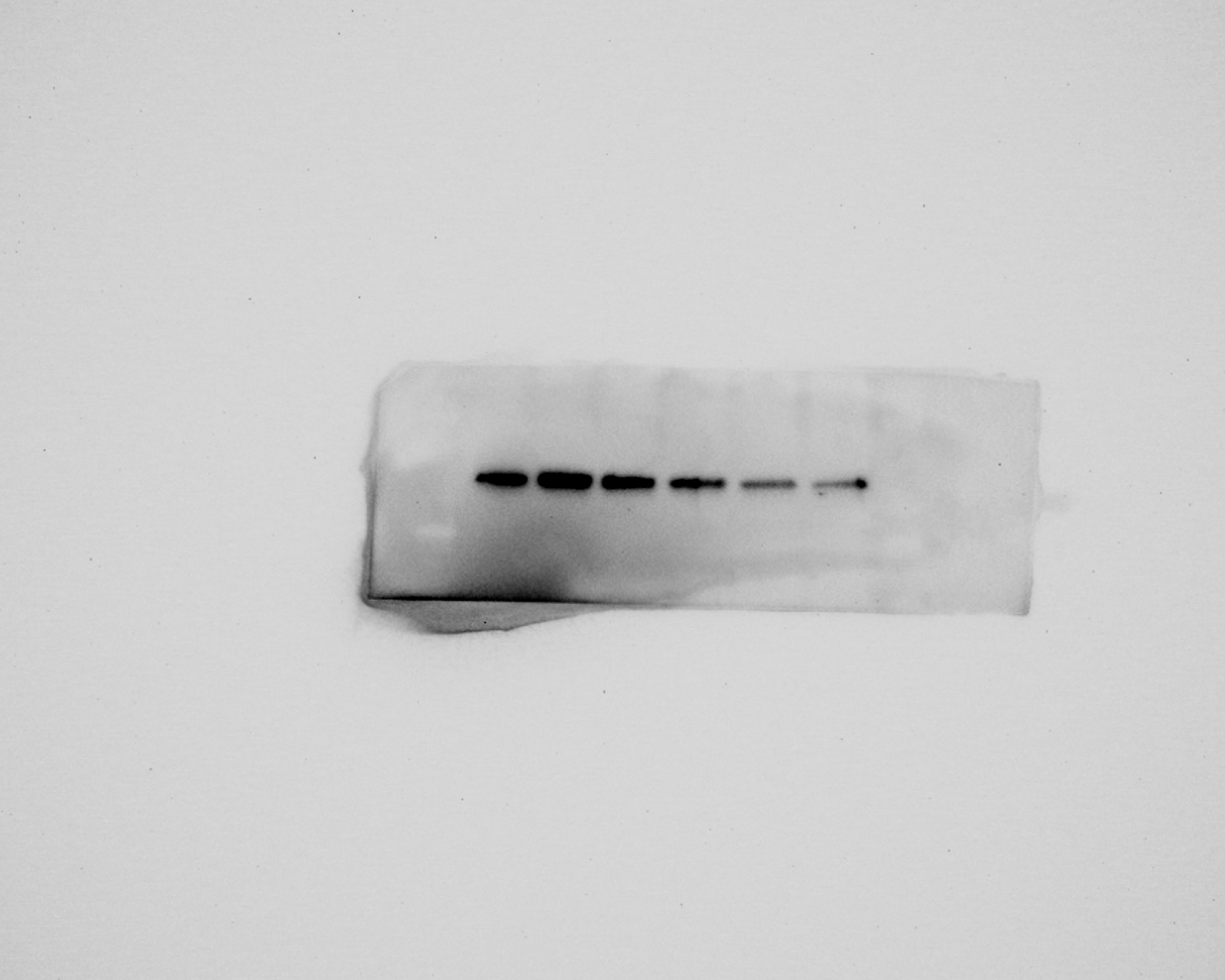


BCL-X_L_


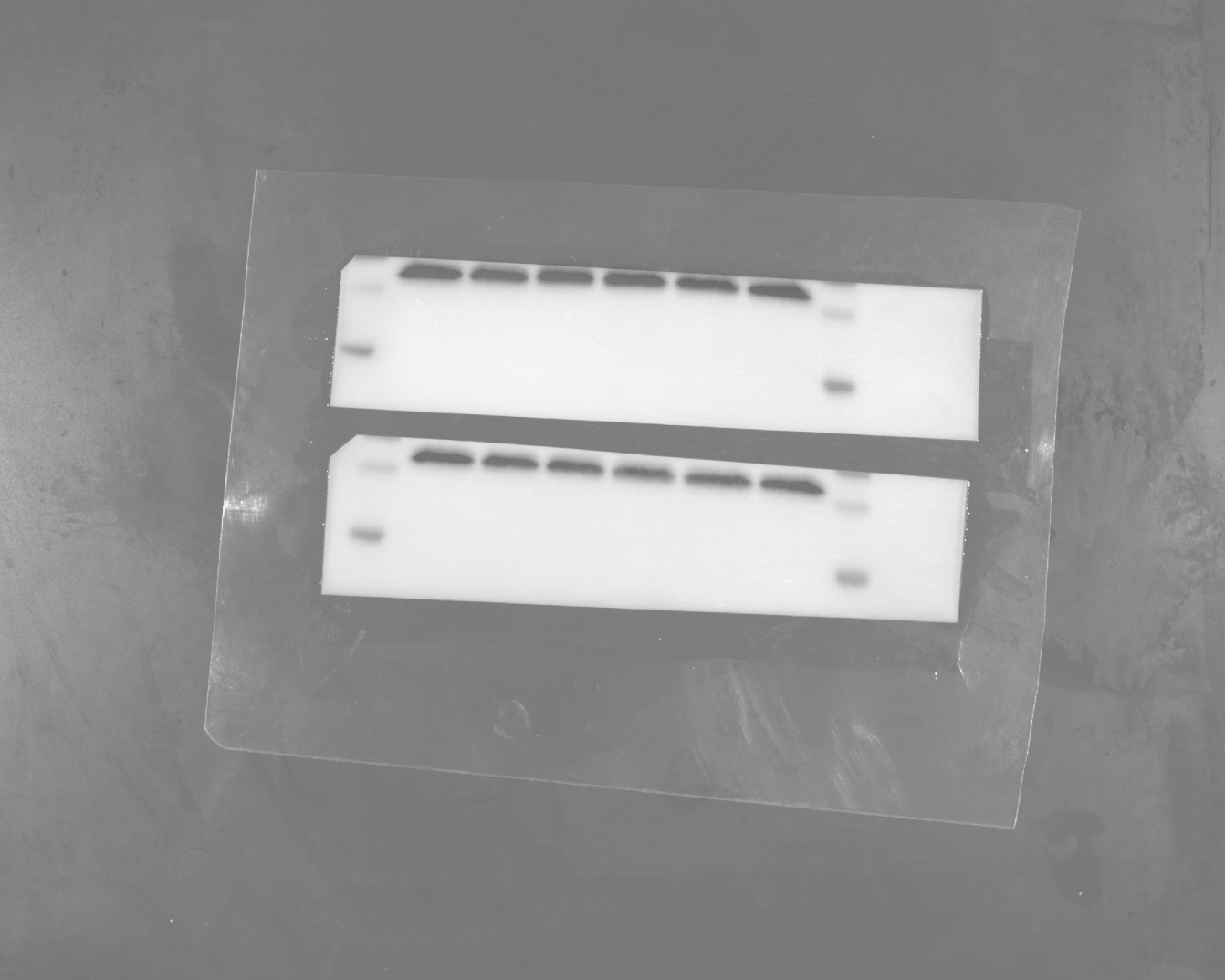


BCL-2


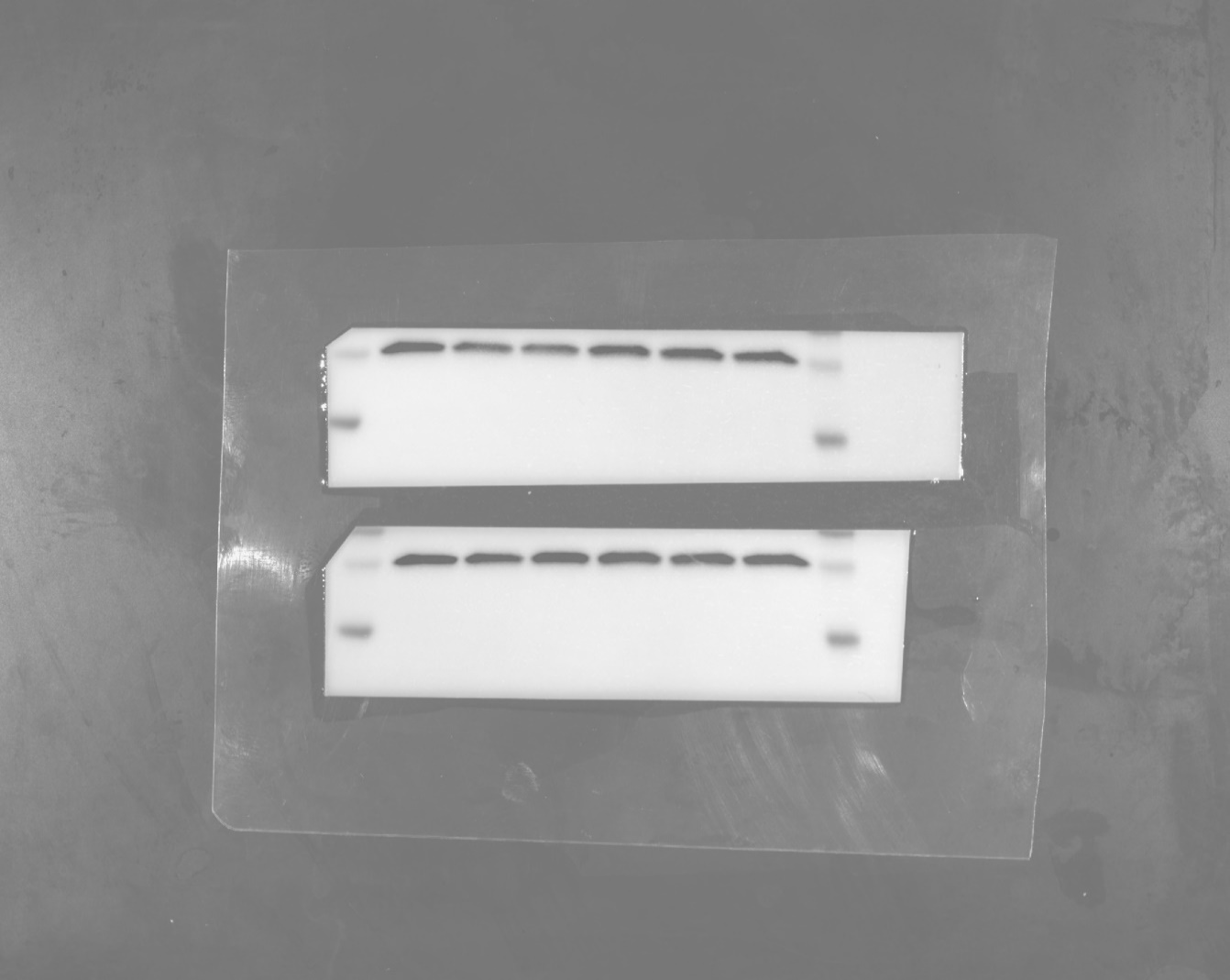


β-Tubulin


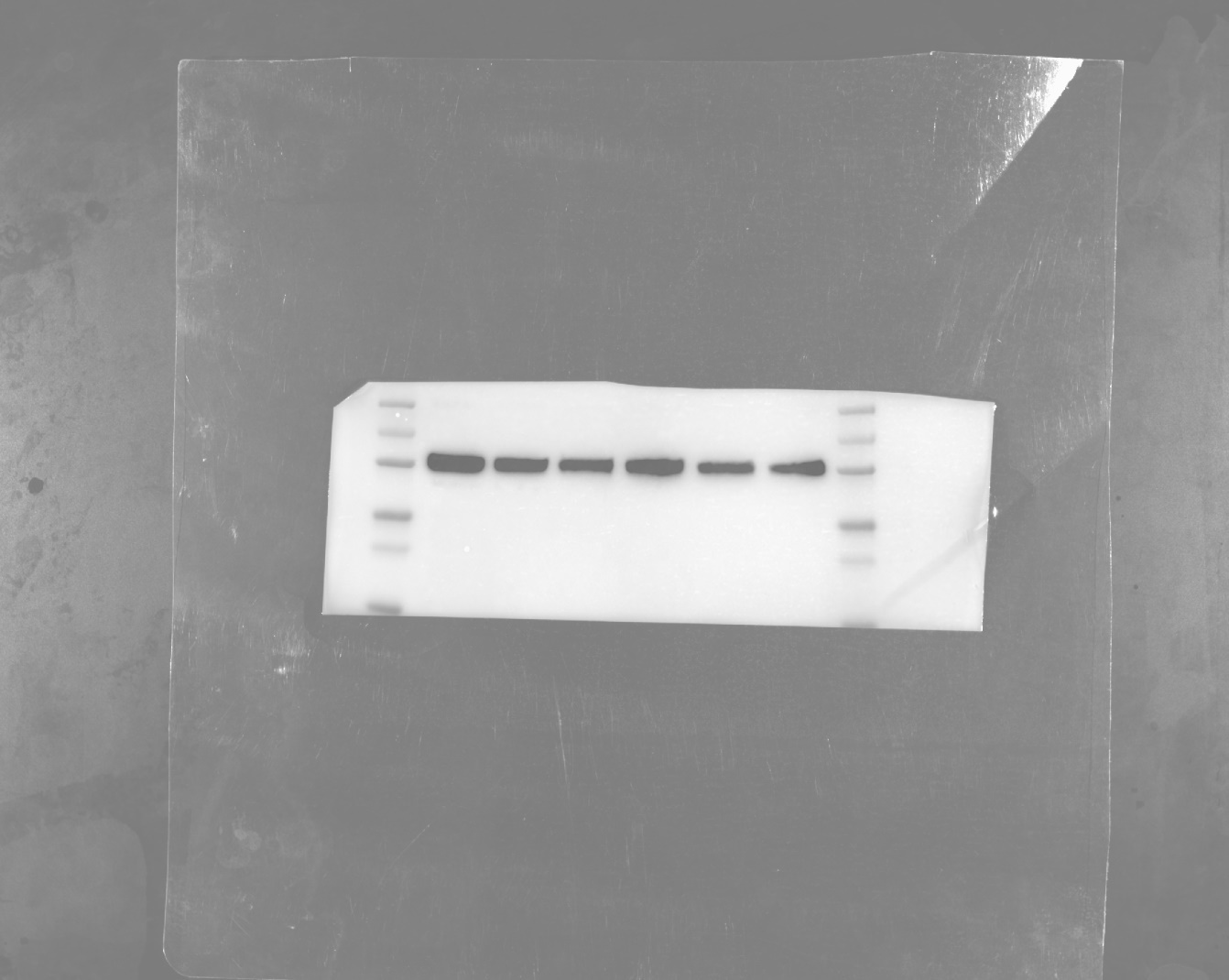


**Figure 3c: H1048**

p-4EBP1^T37/46^


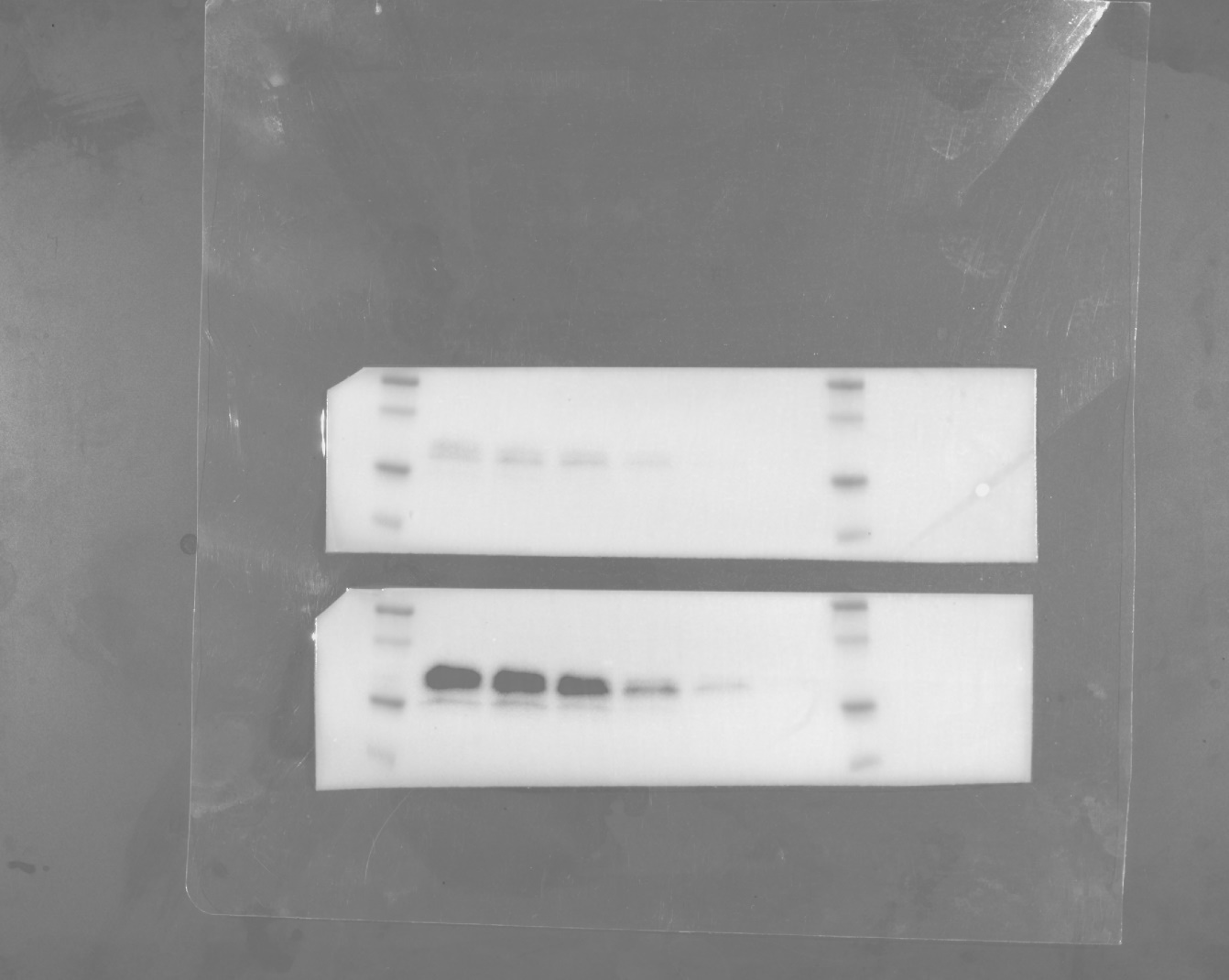


4EBP1


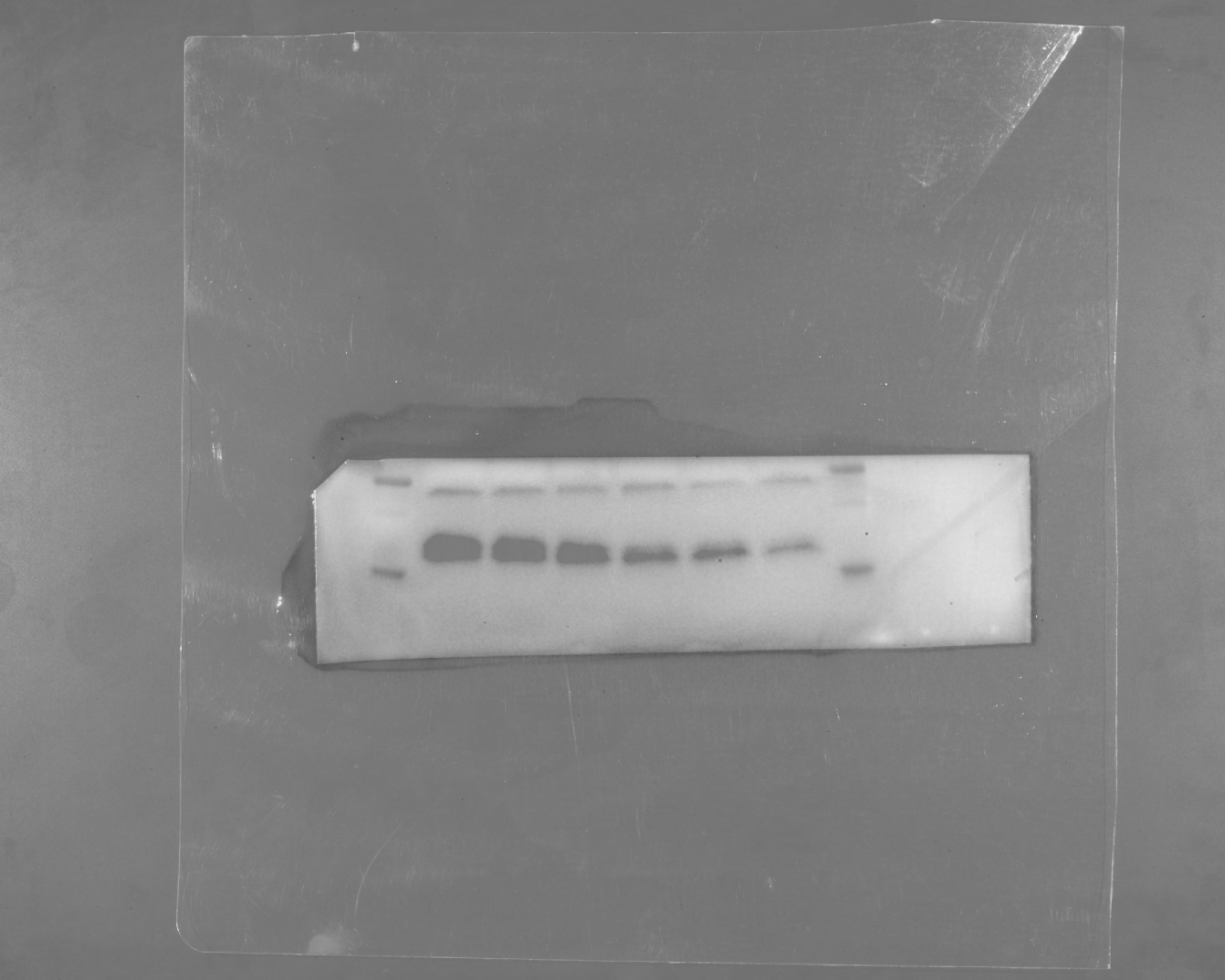


p-S6^S240/244^


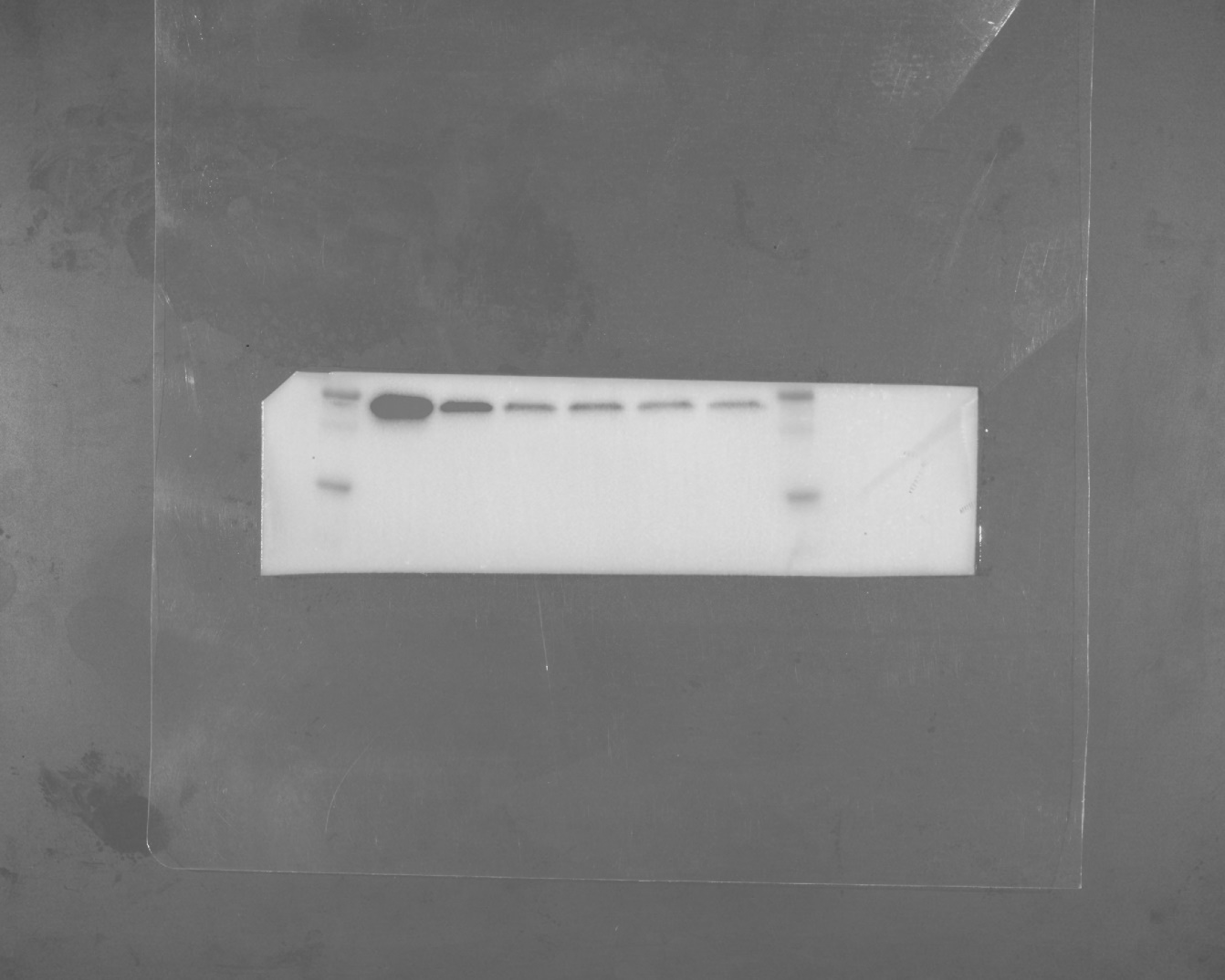


S6


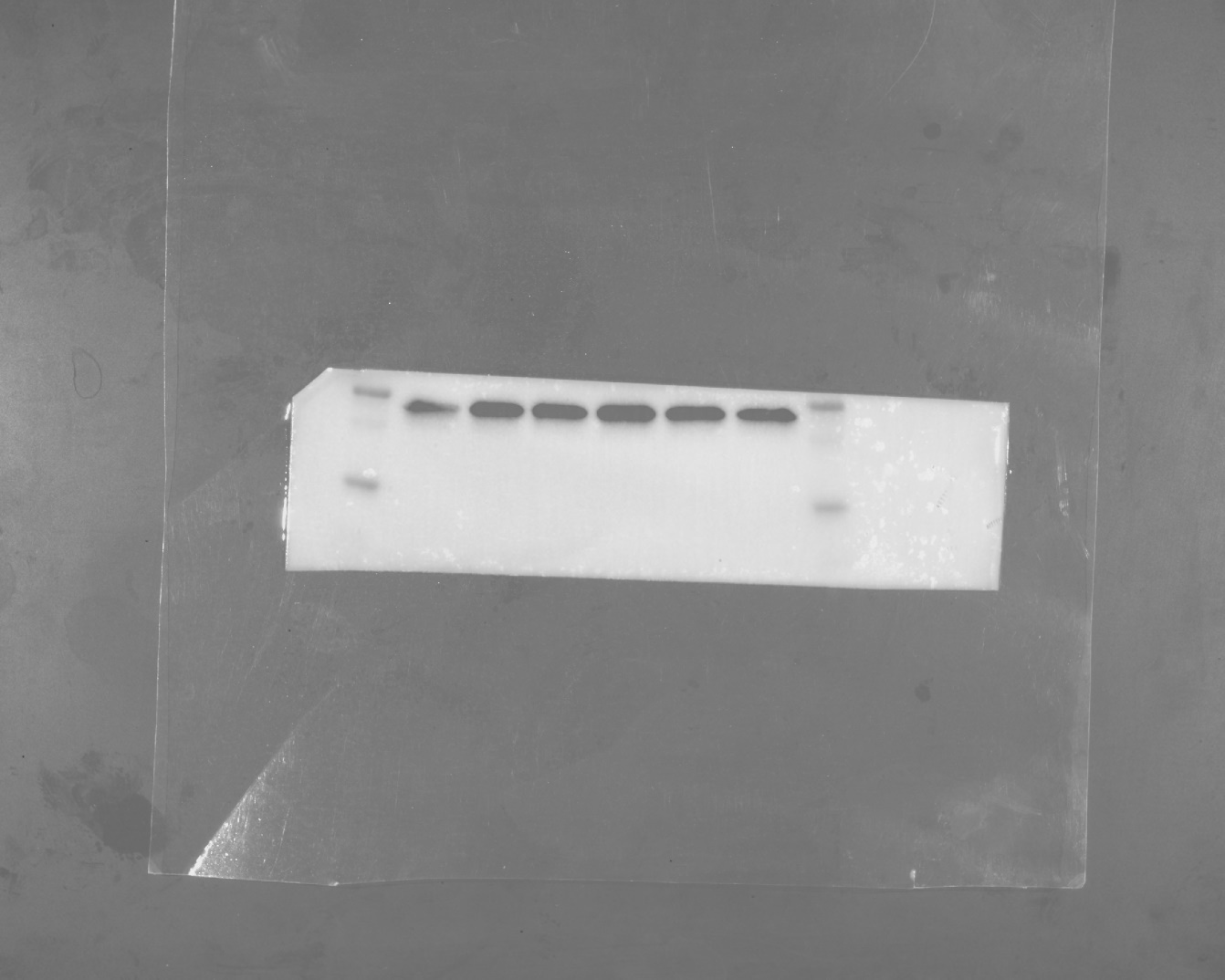


β-Tubulin


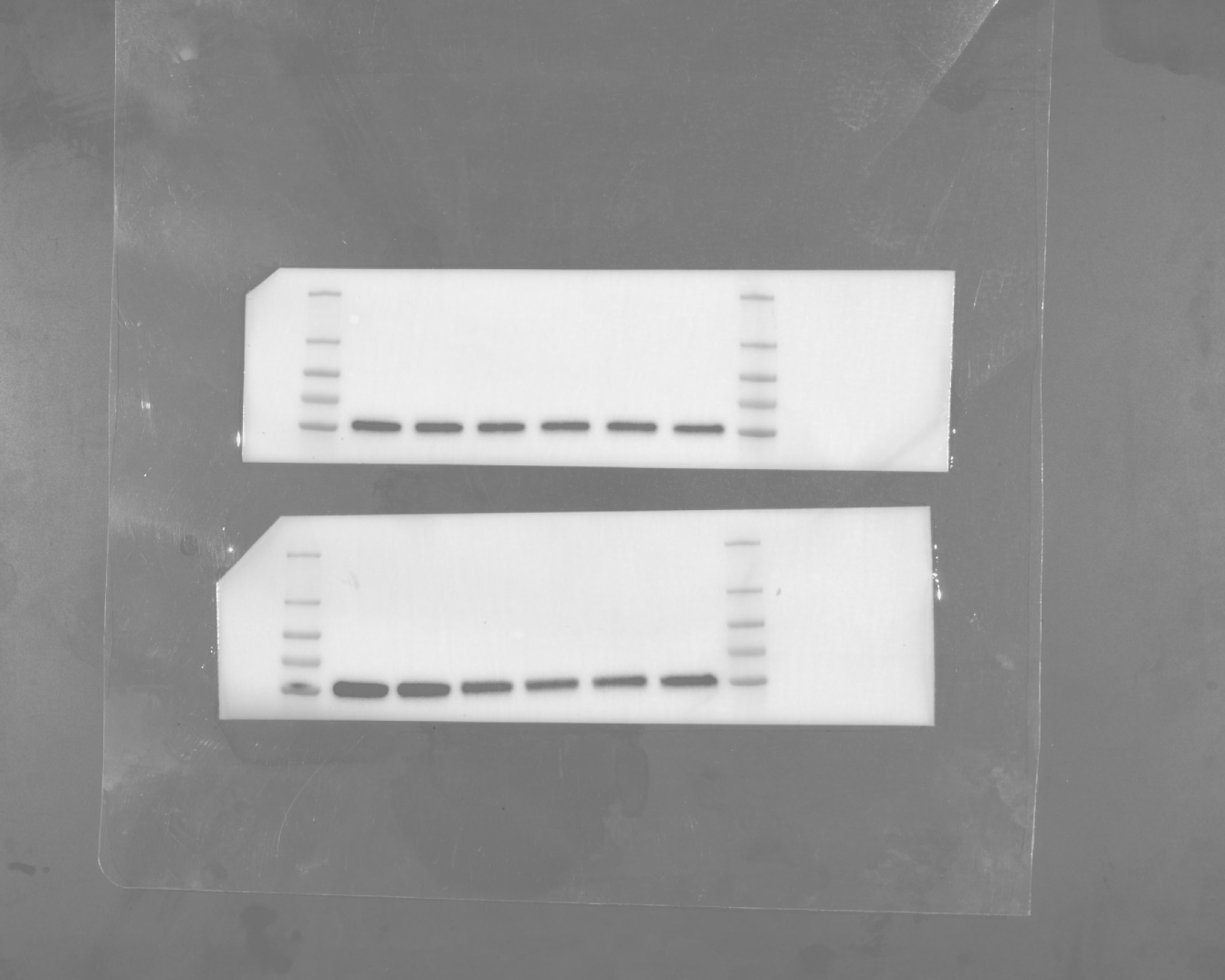


**Figure 3c: H1048**

MCL-1


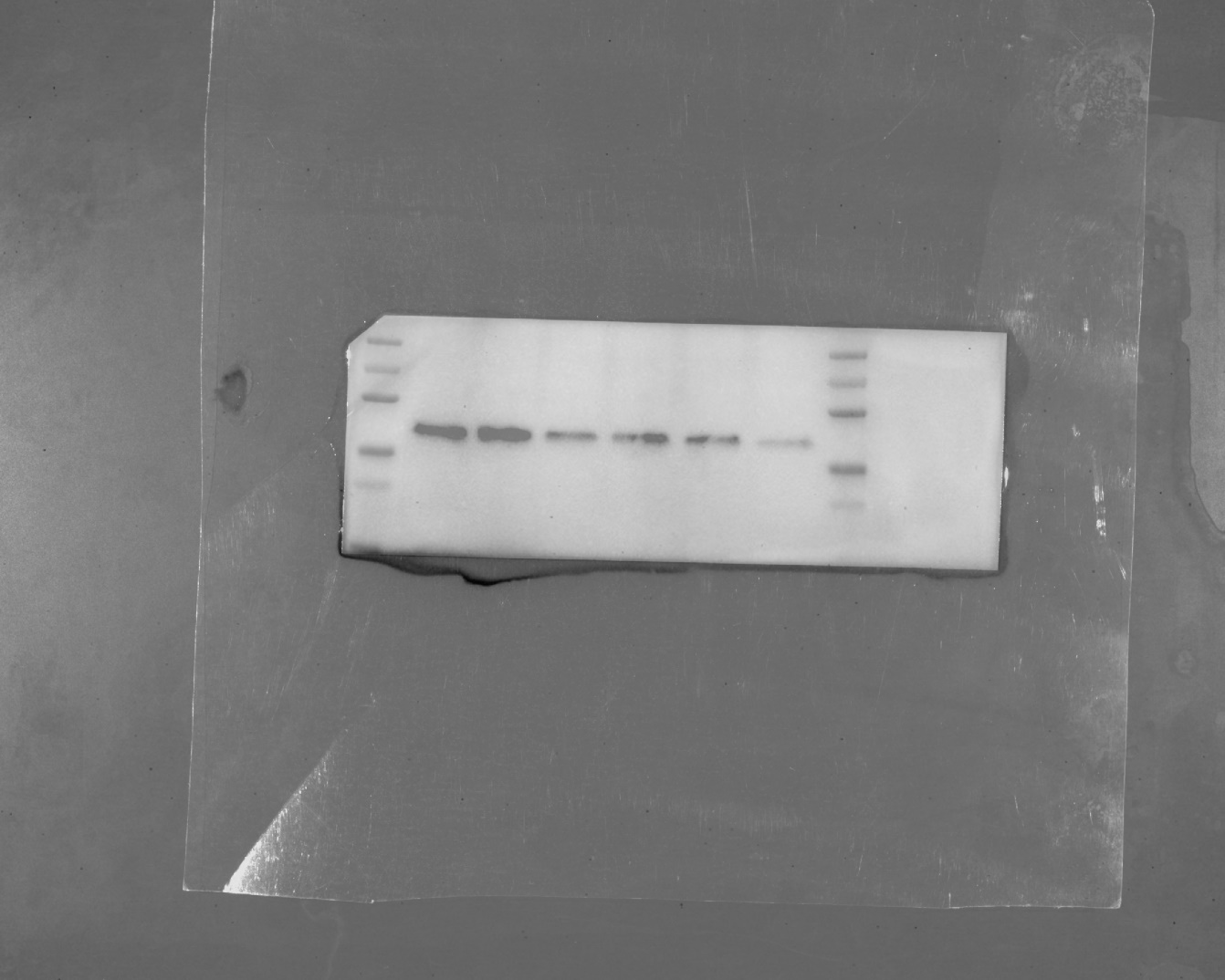


BCL-X_L_


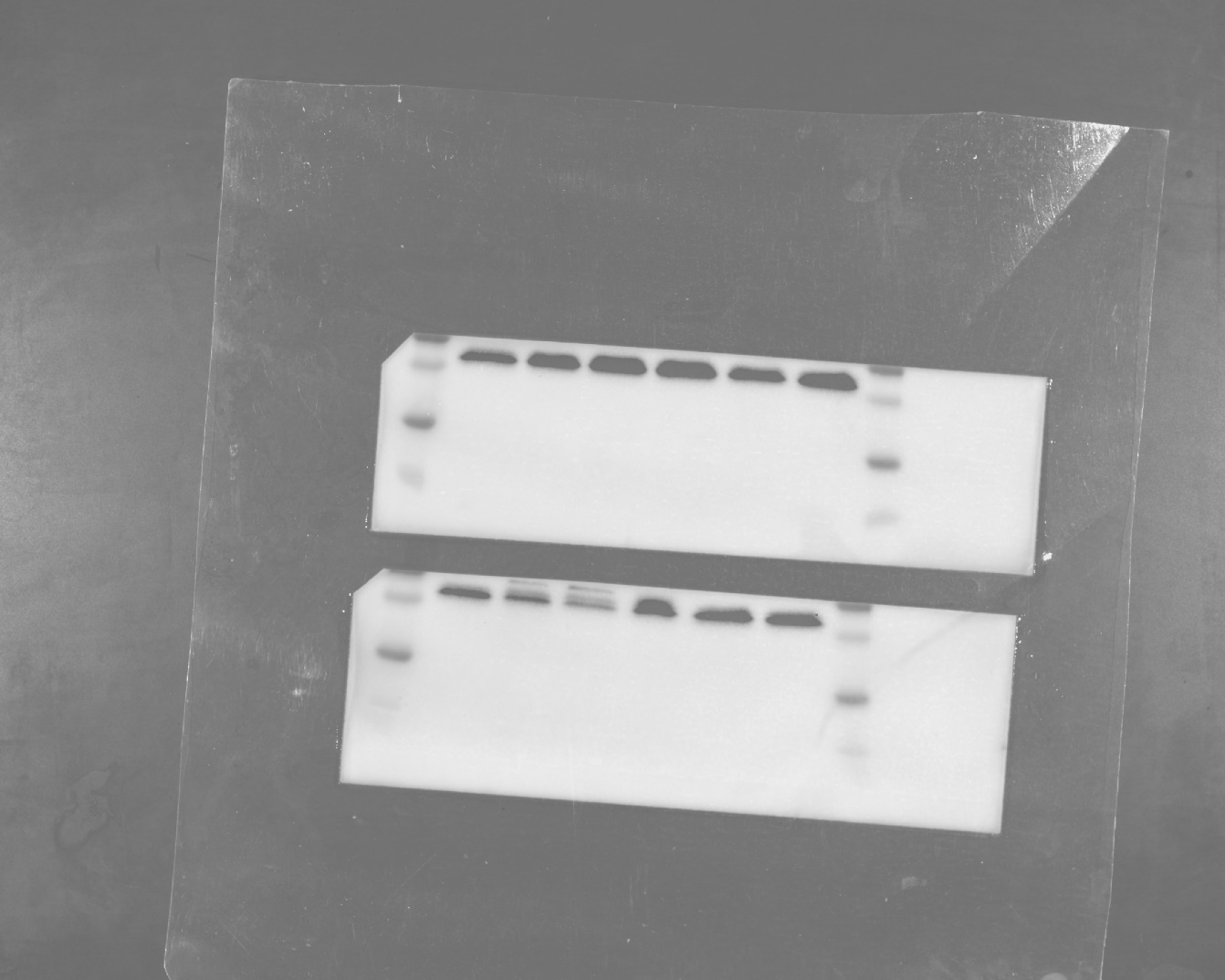


BCL-2


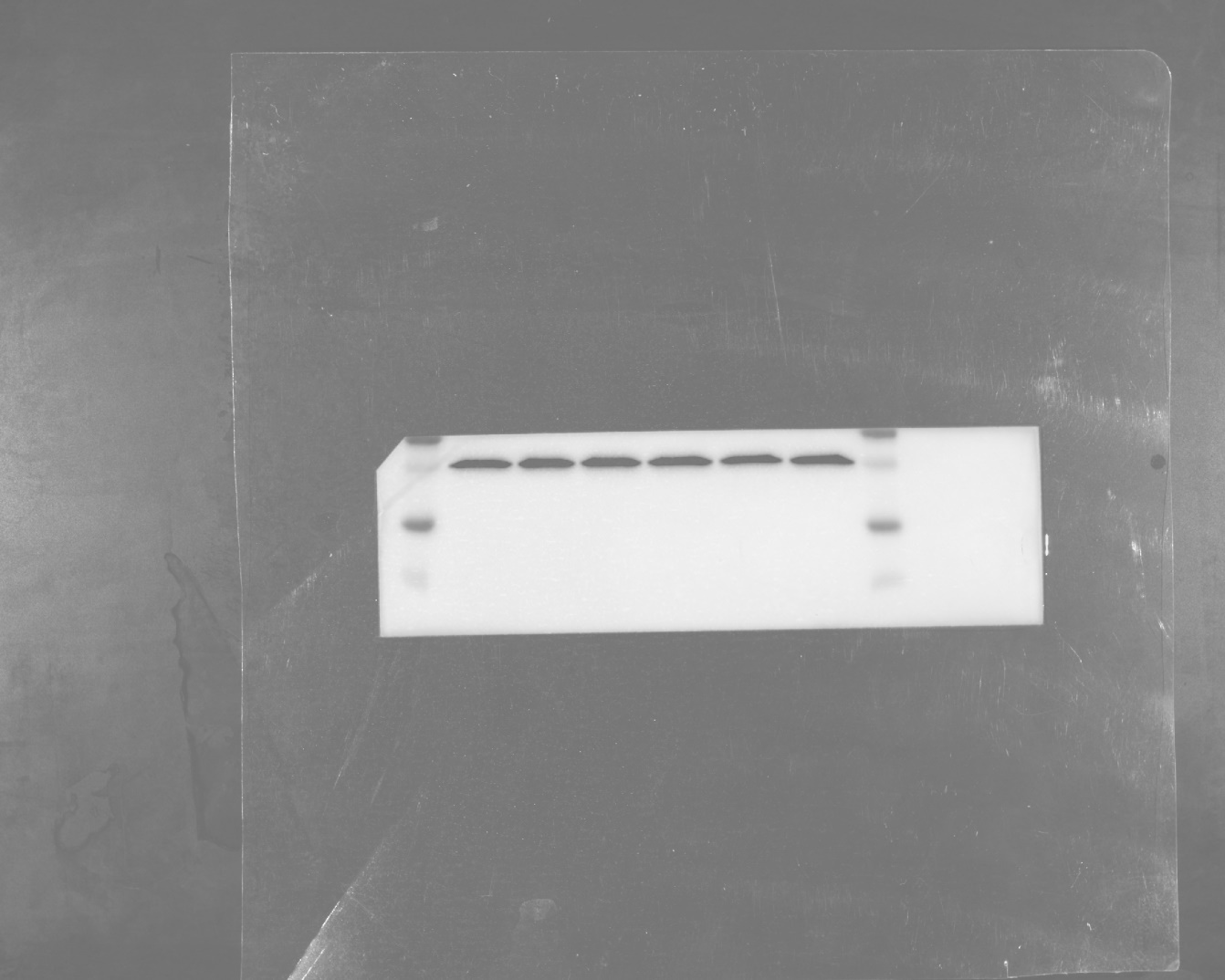


β-Tubulin


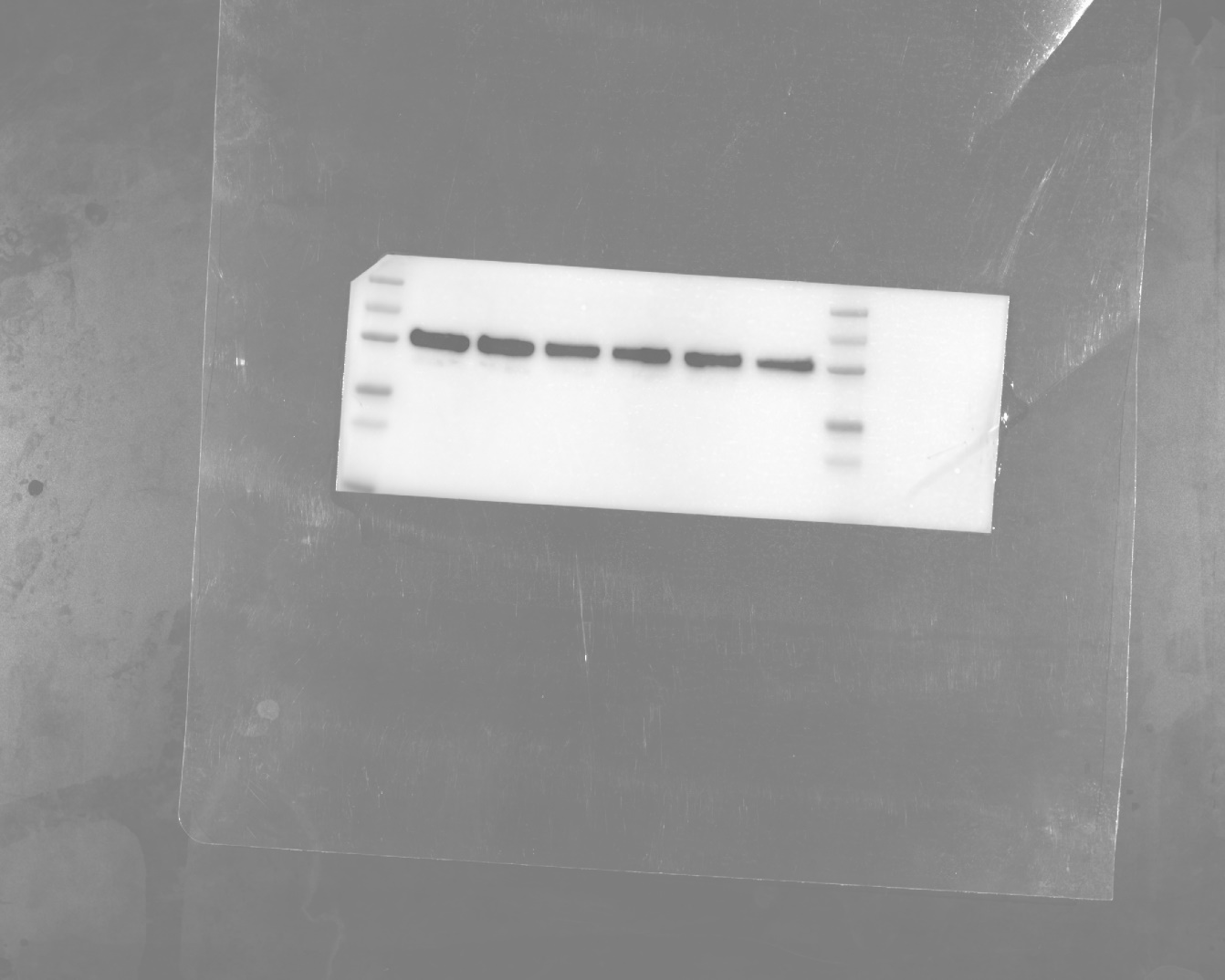


**Figure 3d**

p-4EBP1^T37/46^


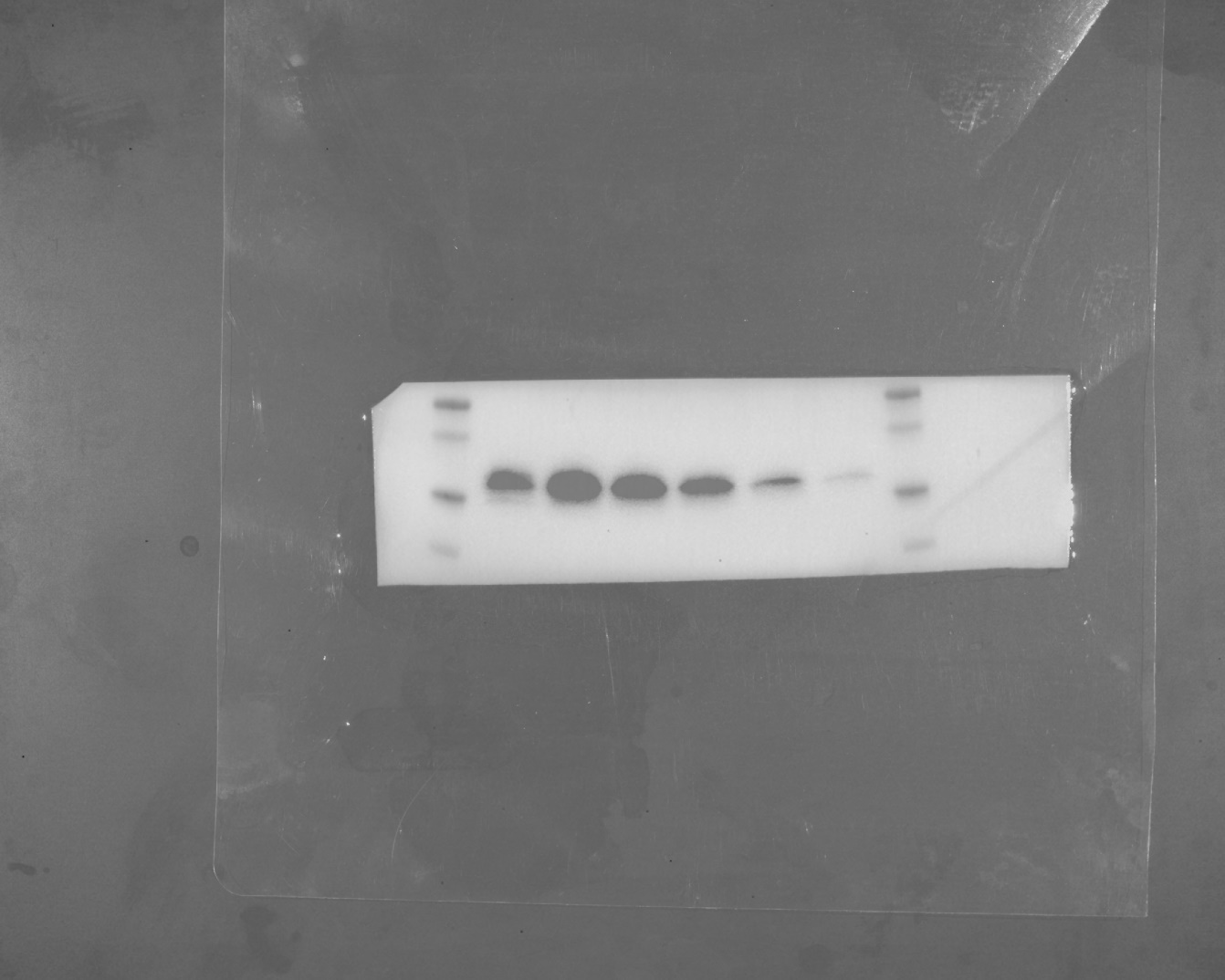


4EBP1


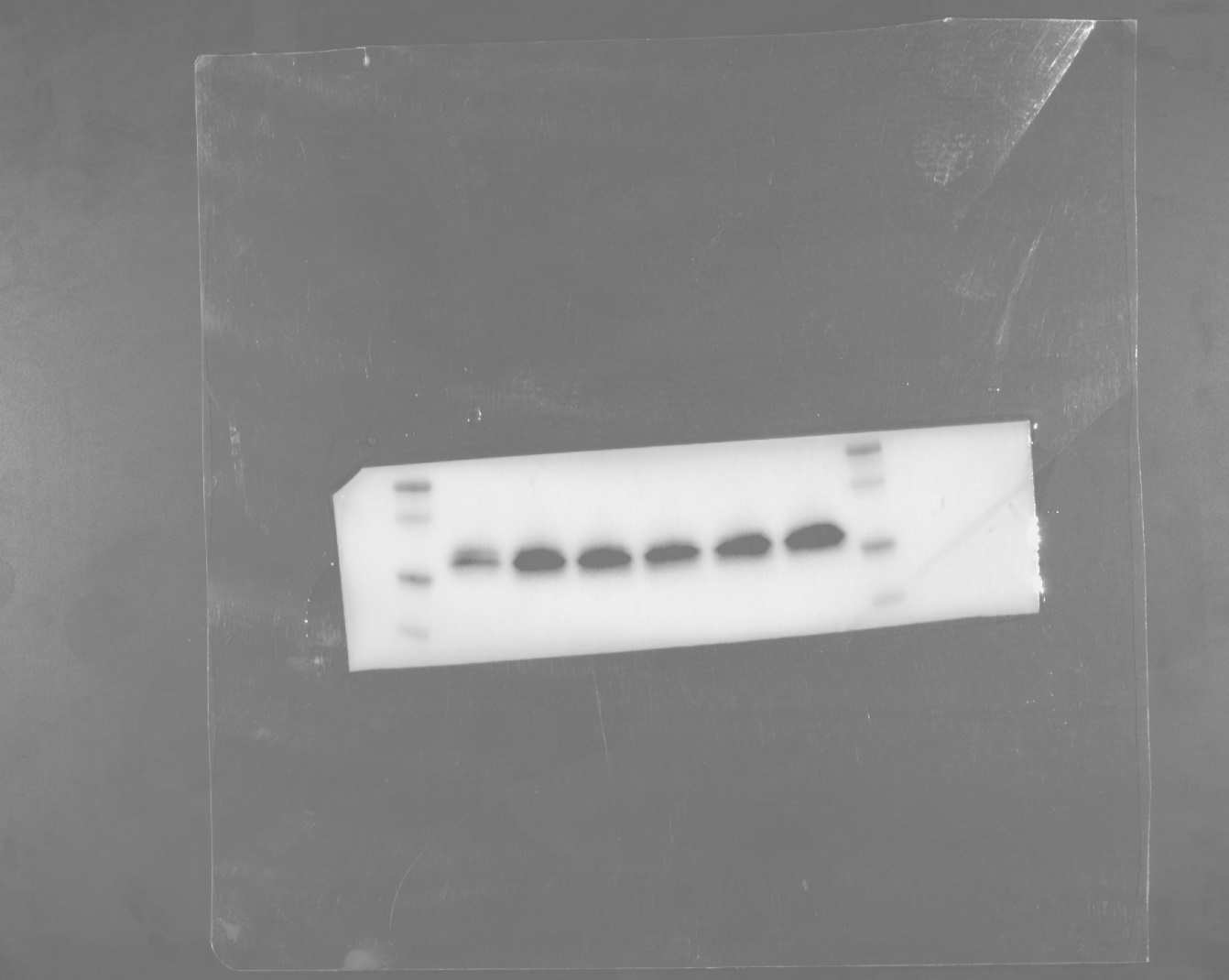


p-S6^S240/244^


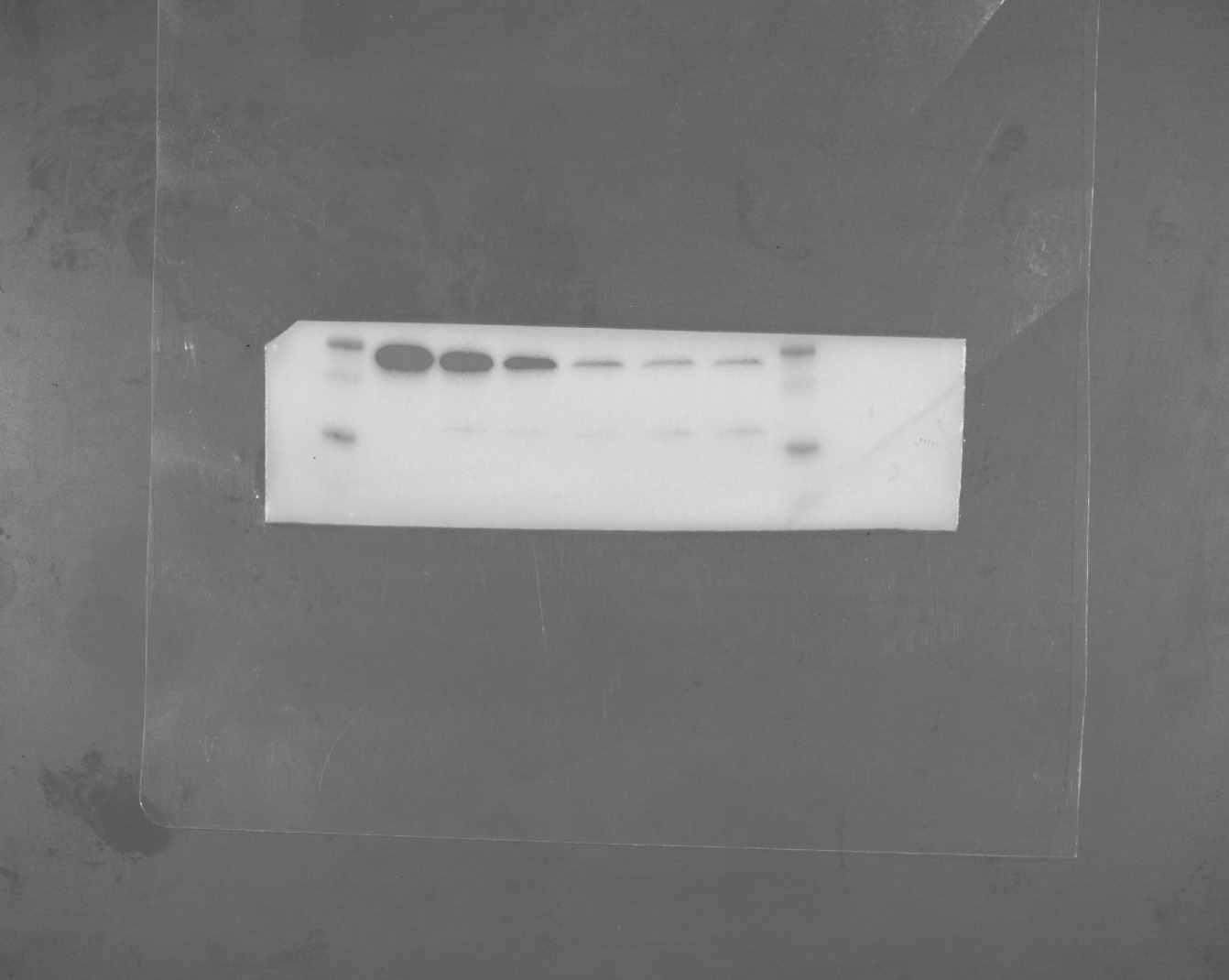


S6


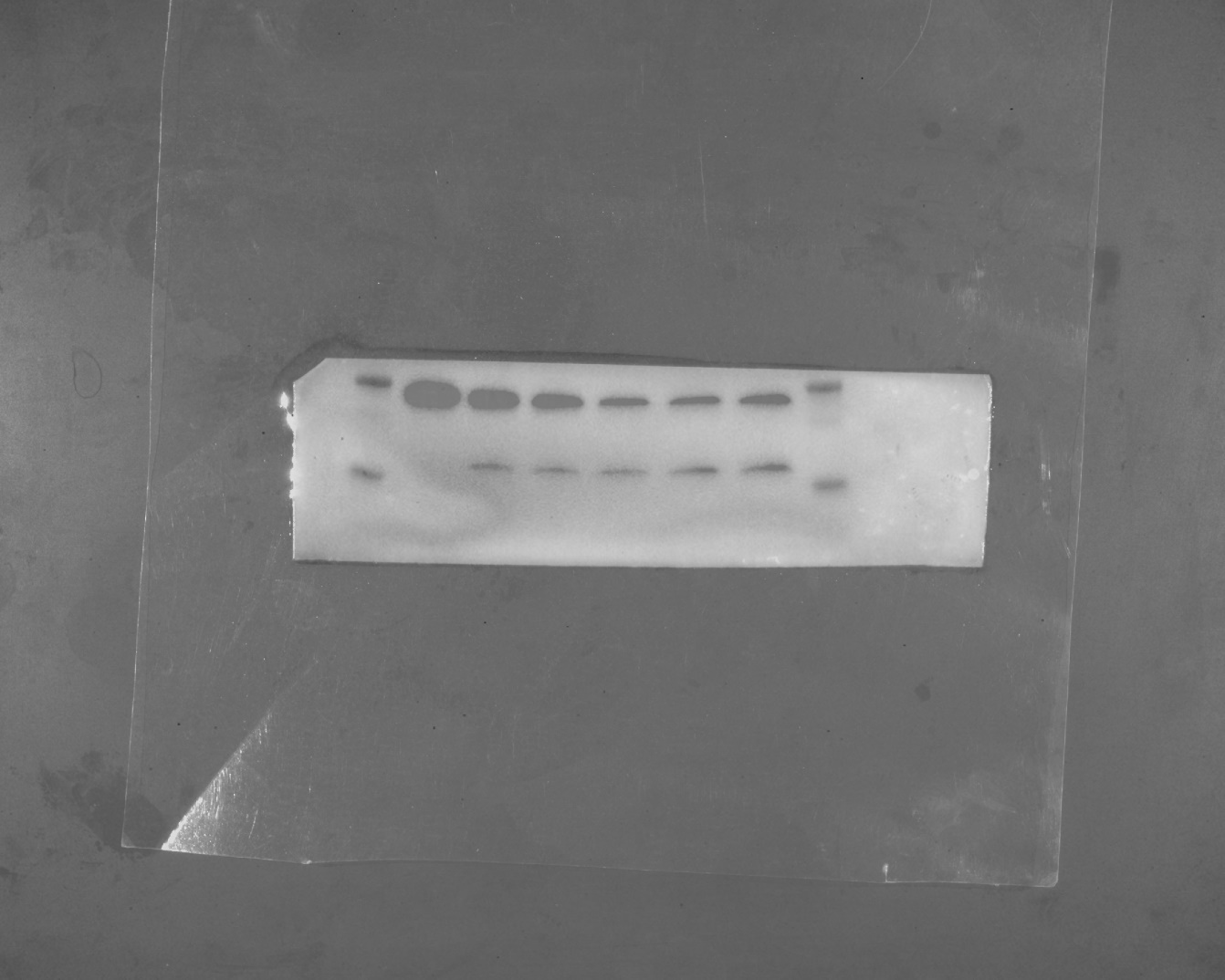


β-Tubulin


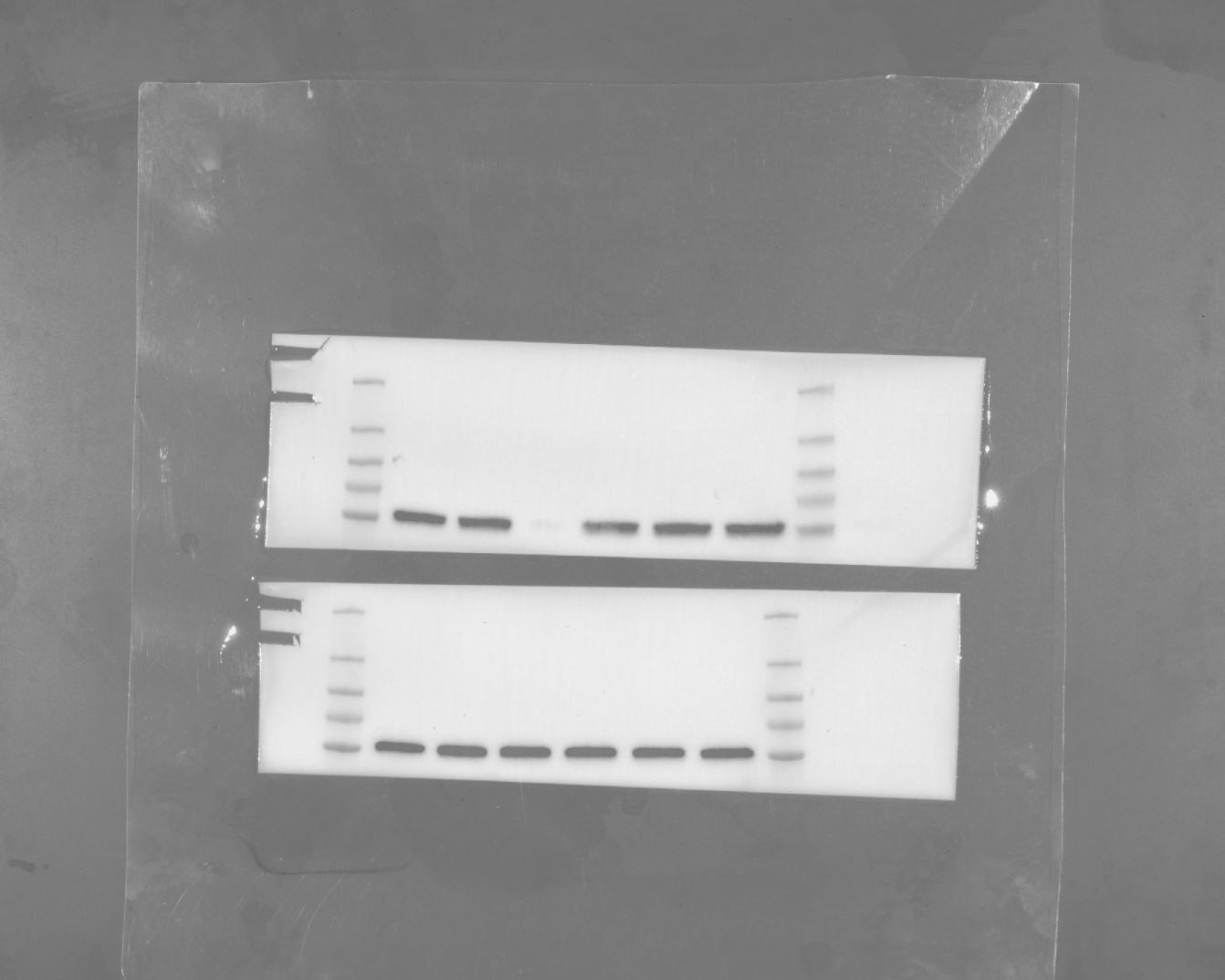


**Figure 3d**

MCL-1


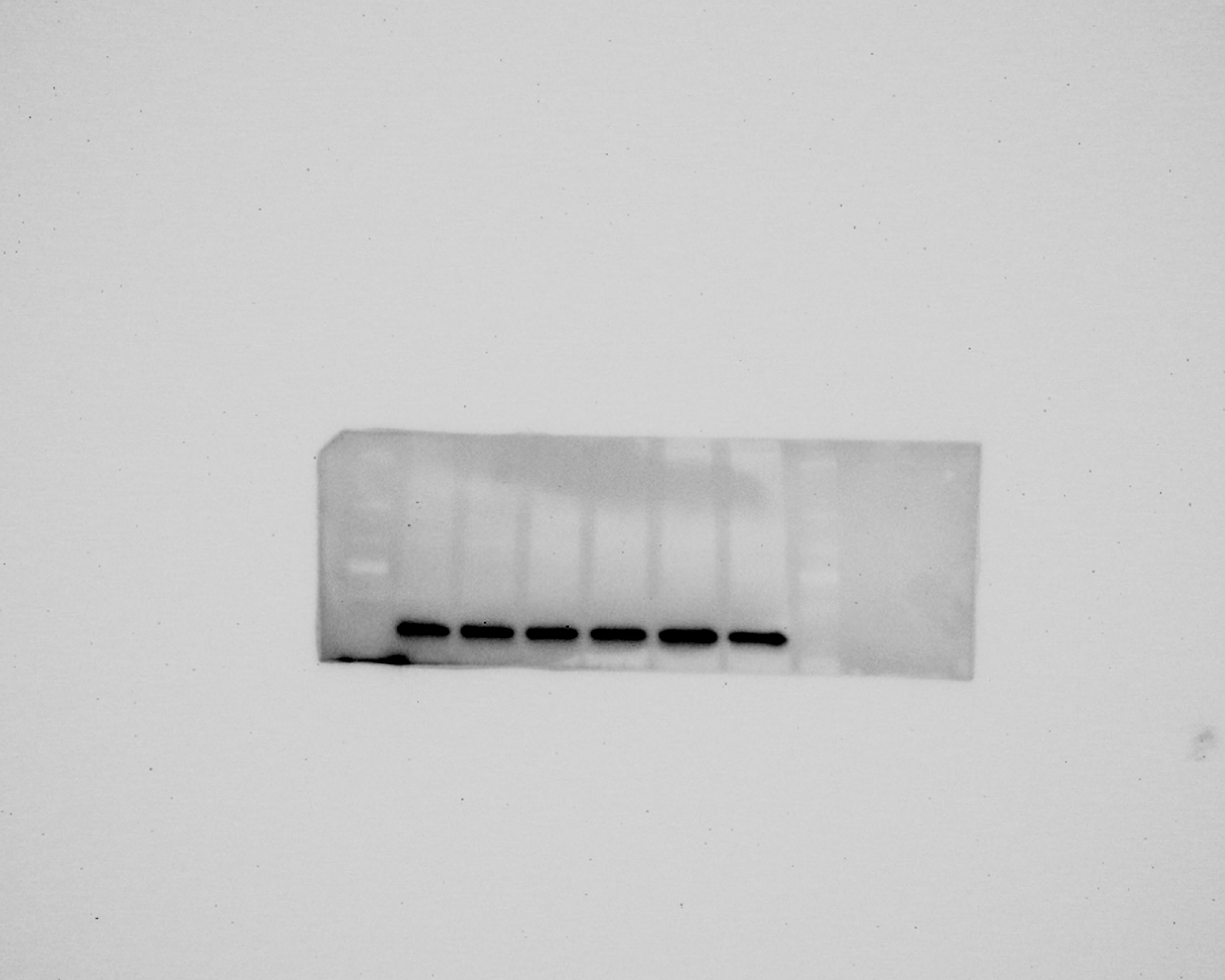


BCL-X_L_


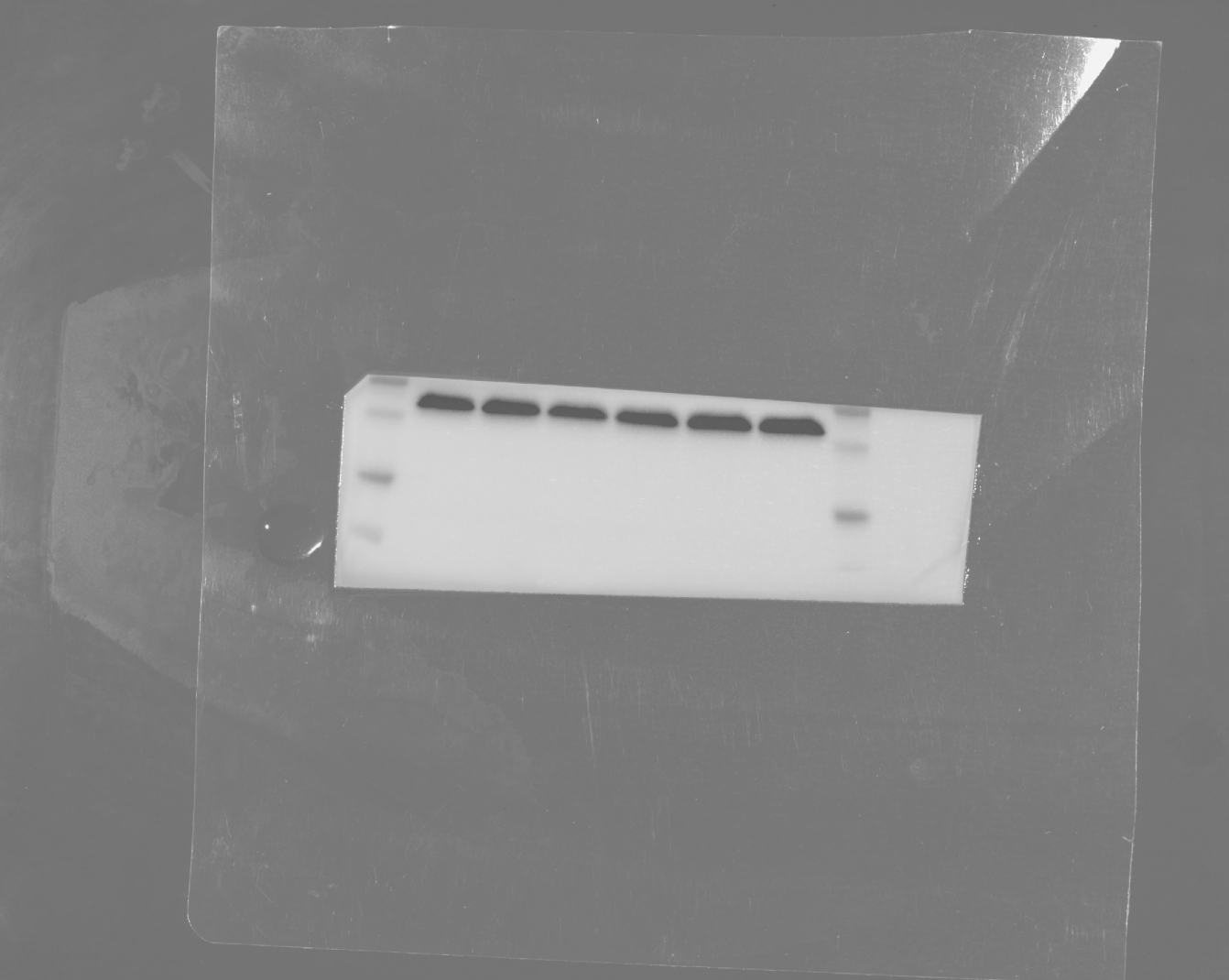


BCL-2


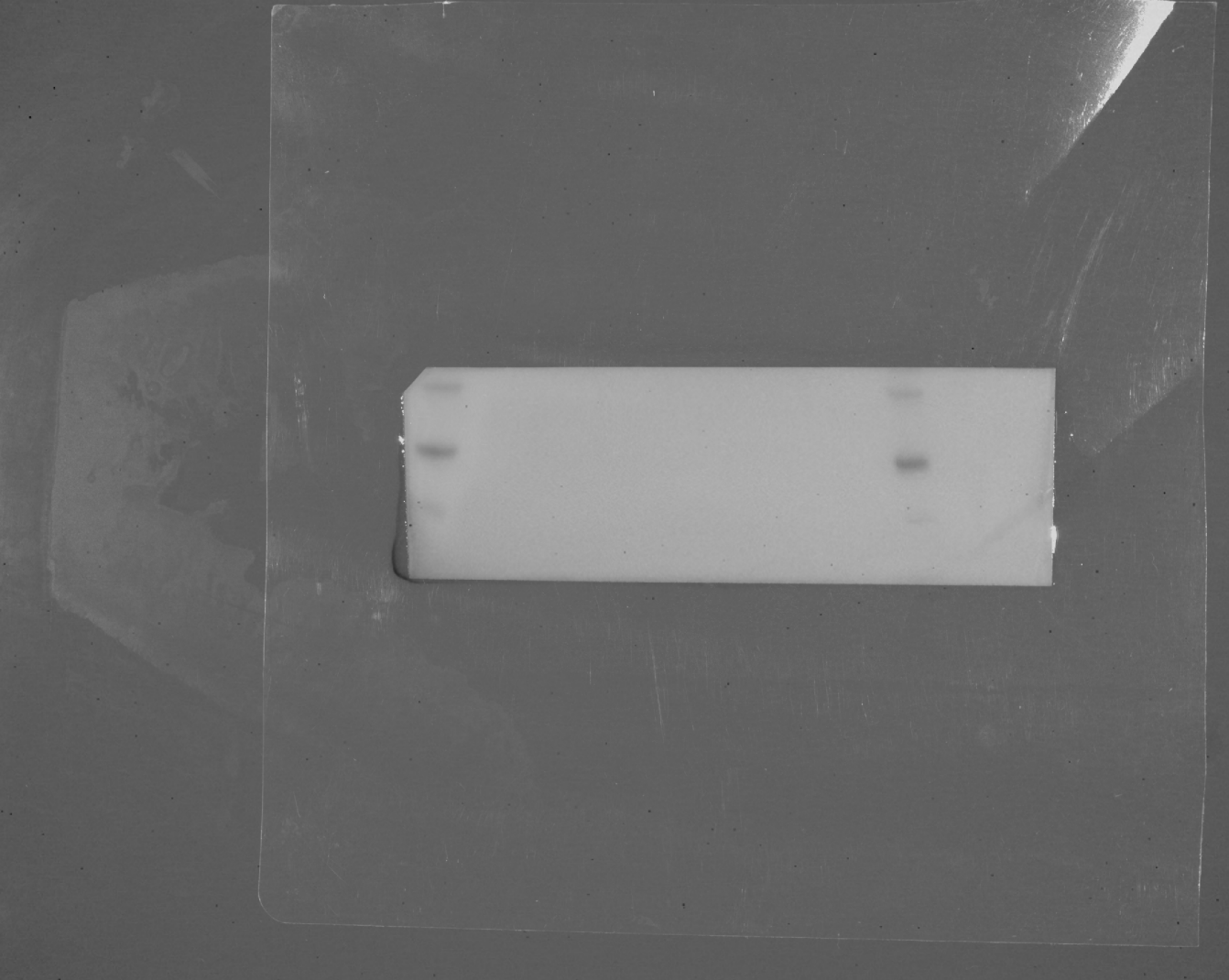


β-Tubulin


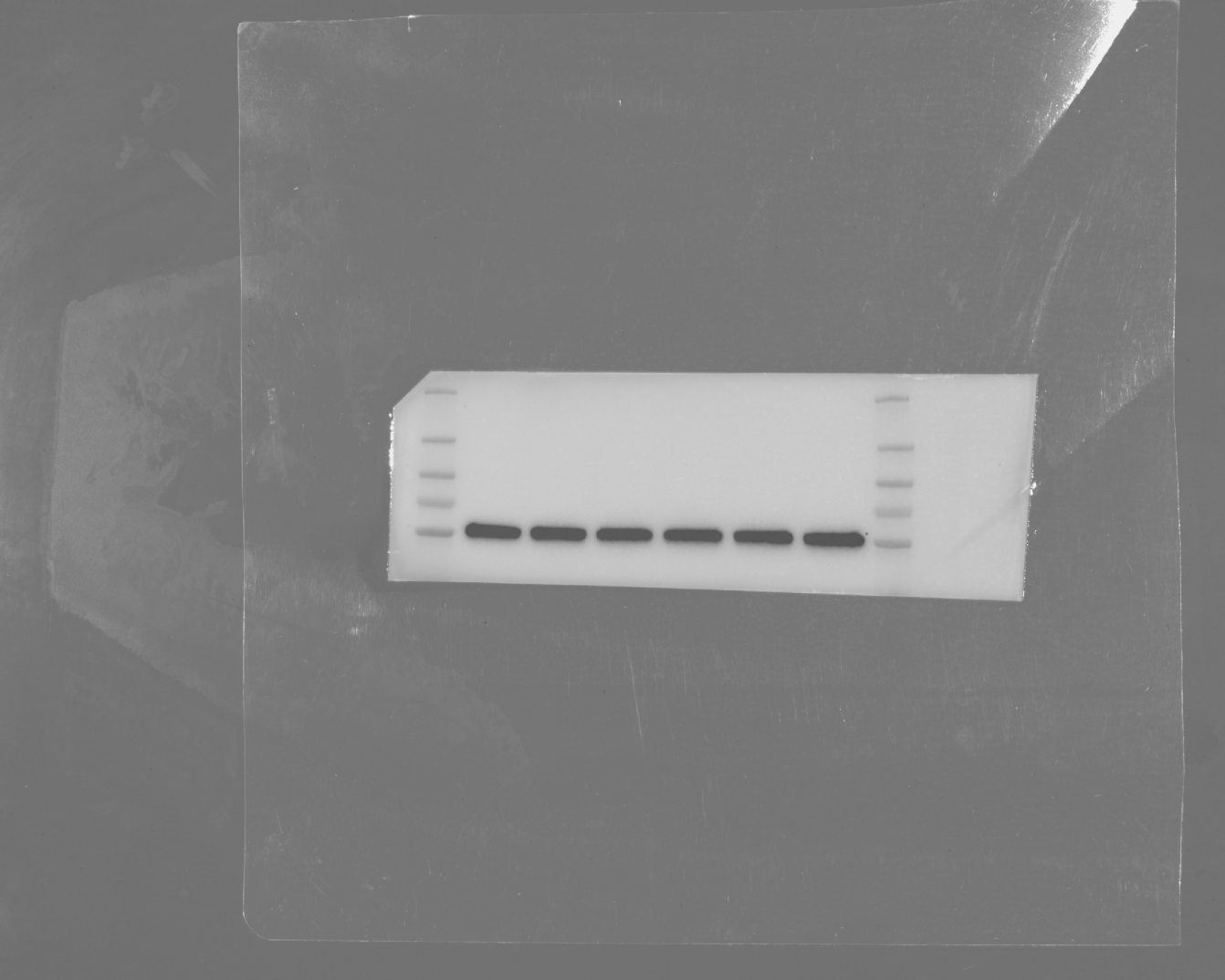


**Figure 4c: H378**

PARP


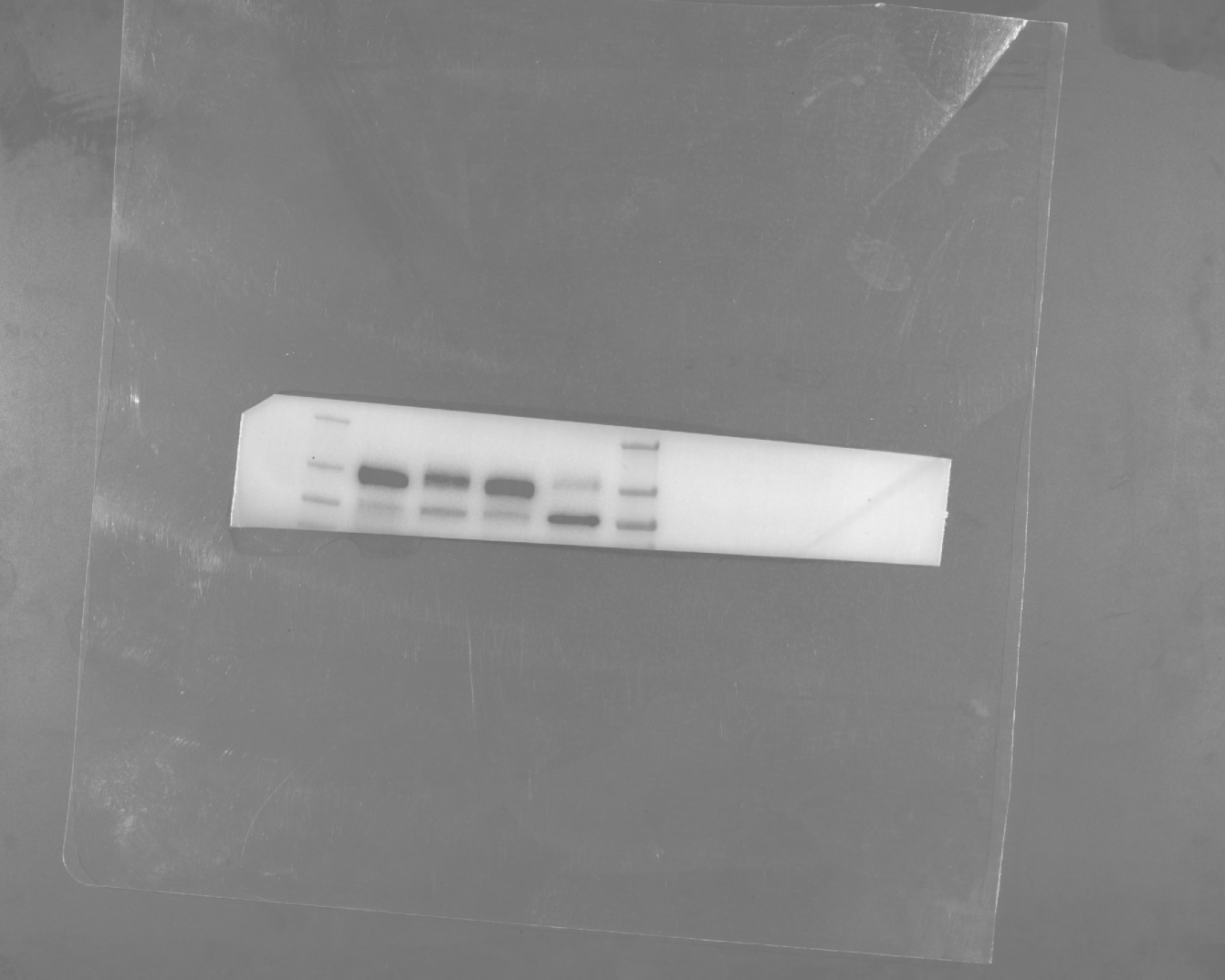


β-Tubulin


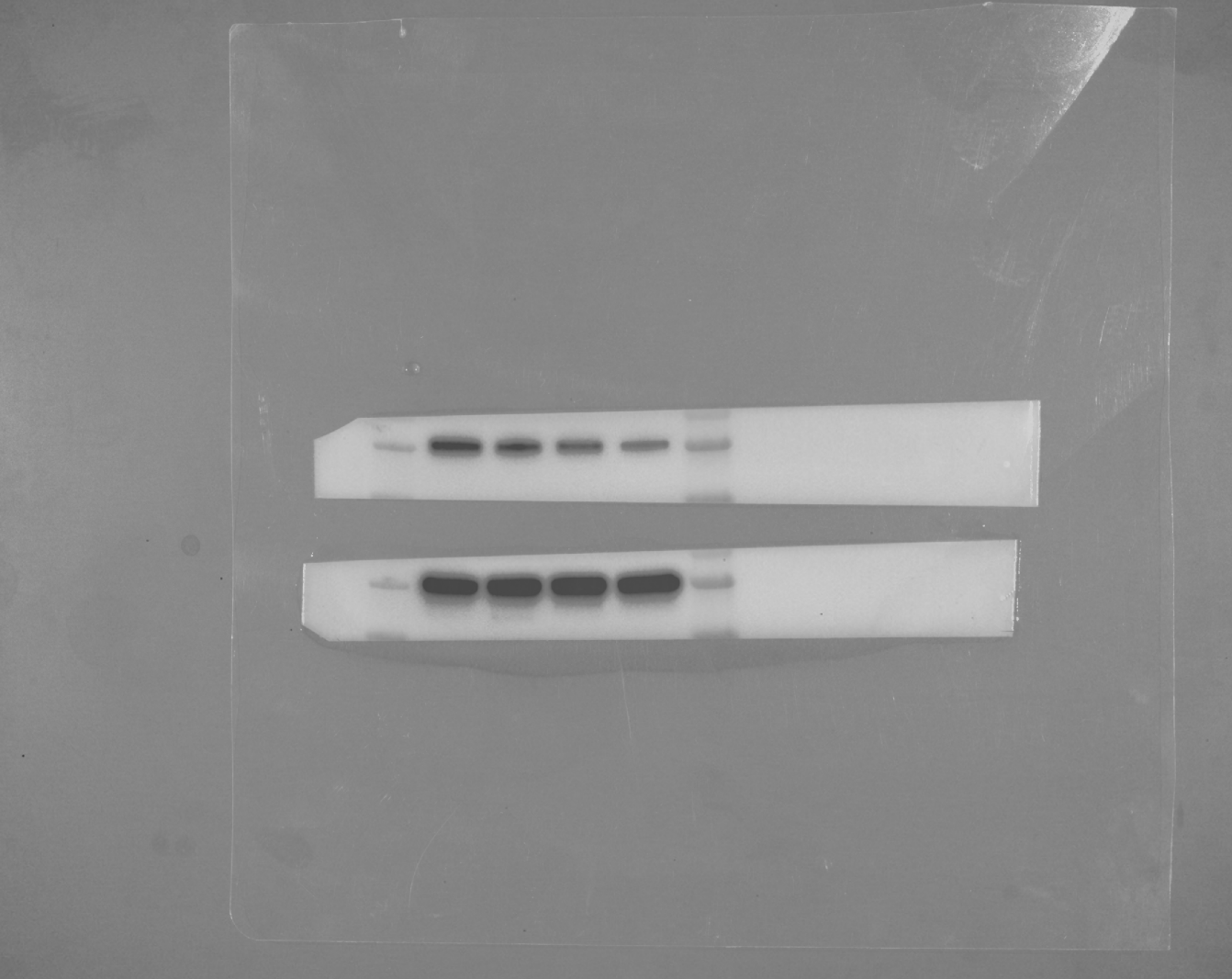


**Figure 4c: H1048**

PARP


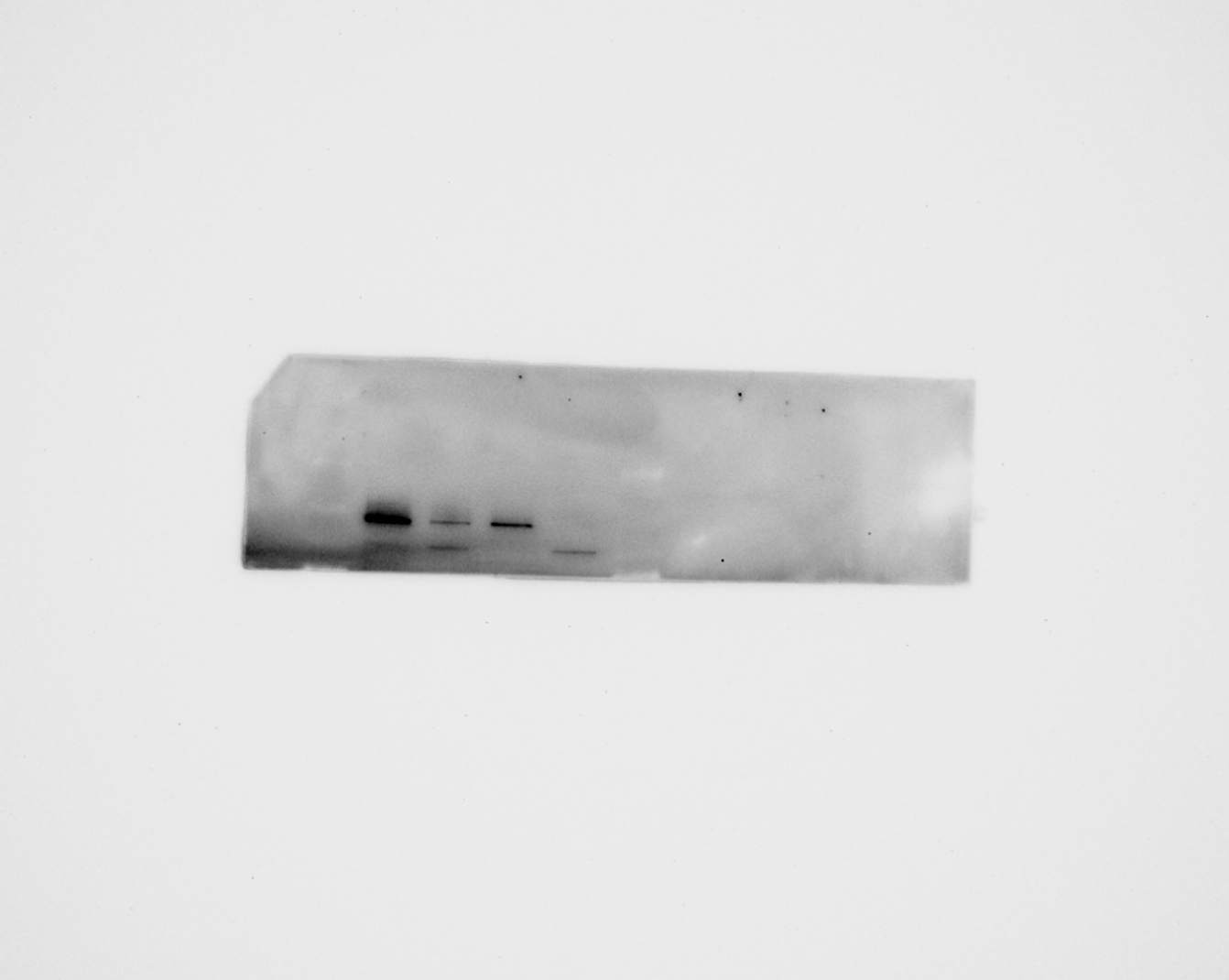


β-Tubulin


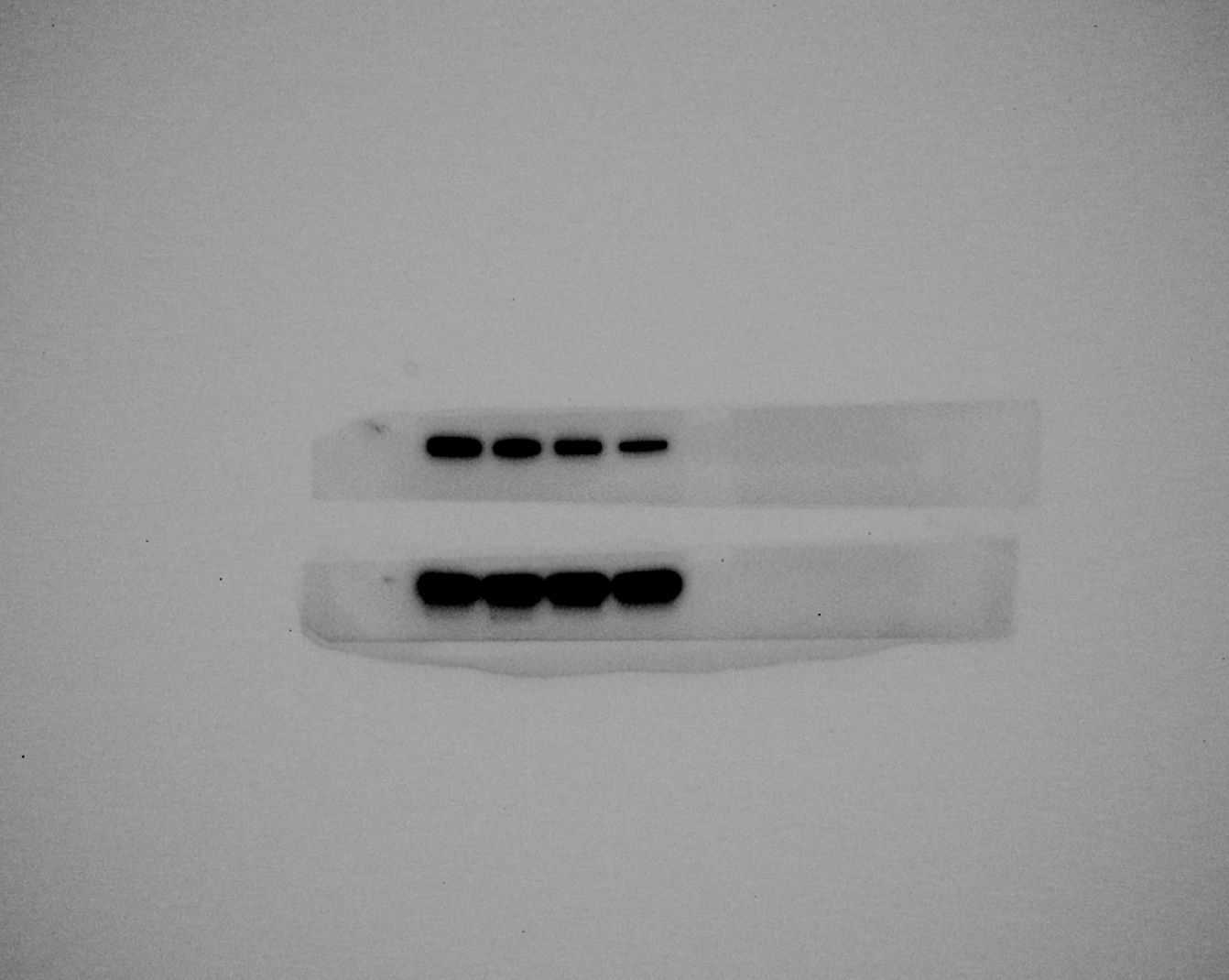


**Figure 4d**

PARP


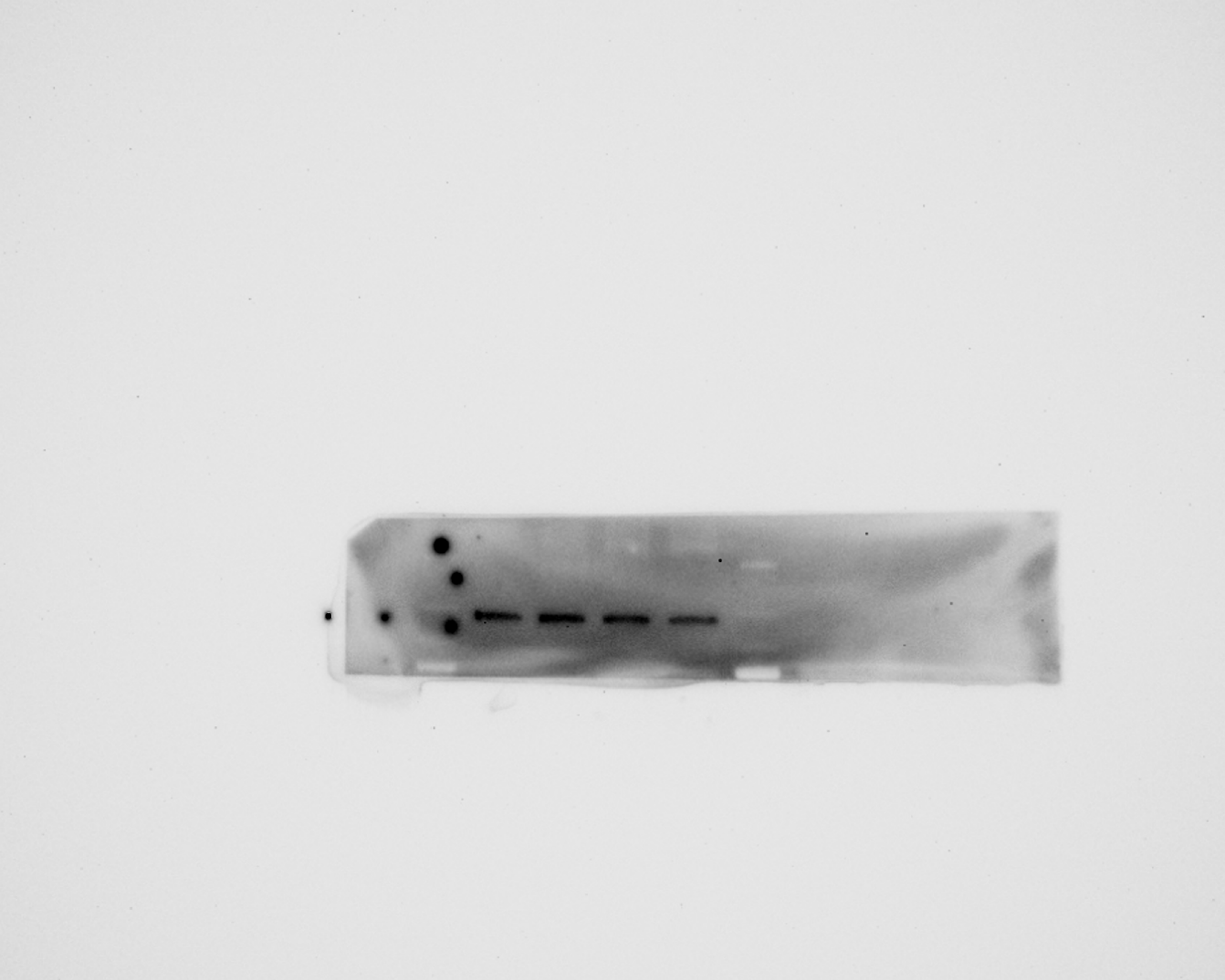


β-Tubulin


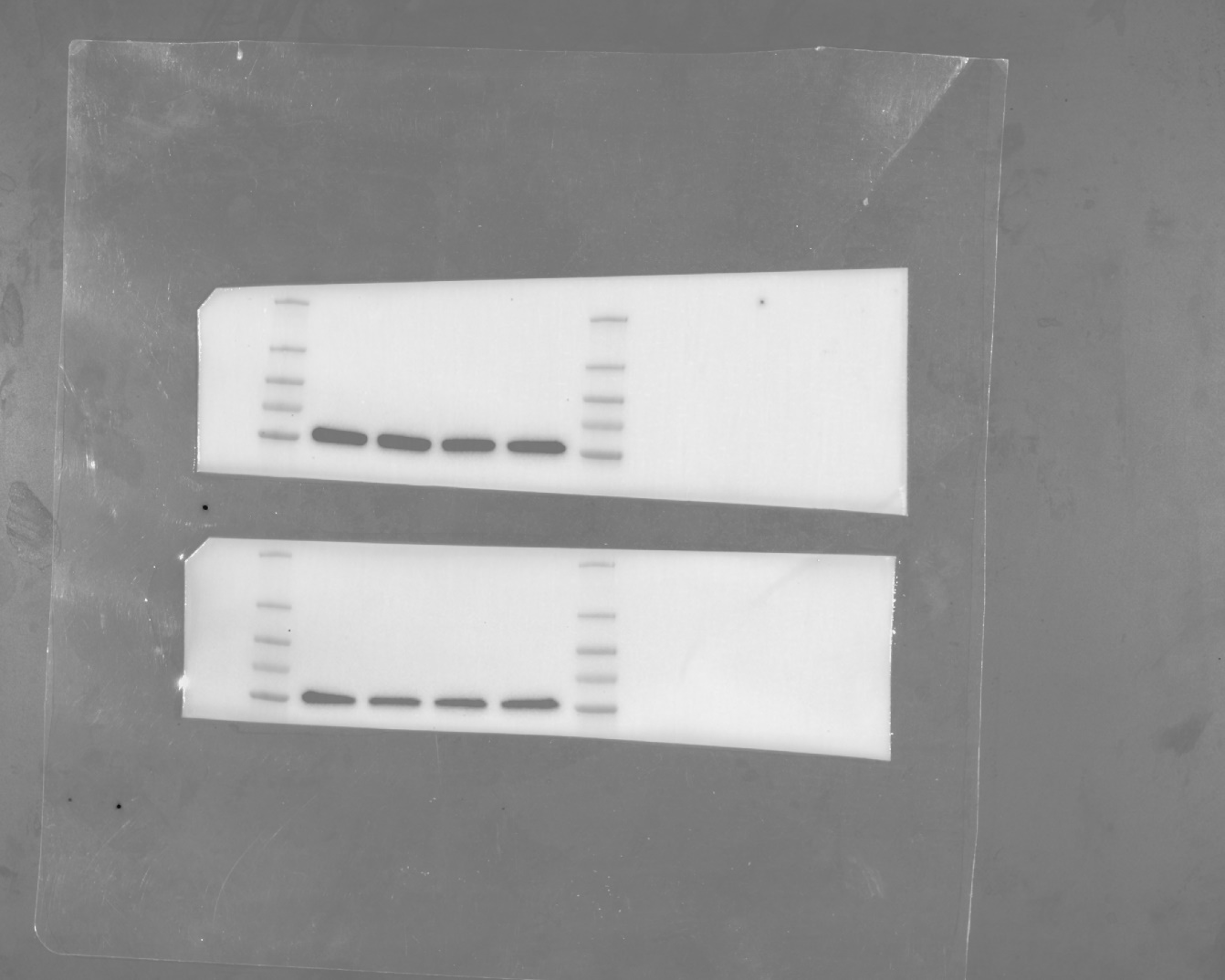


**Figure 4e: IP: MCL-1**

BIM


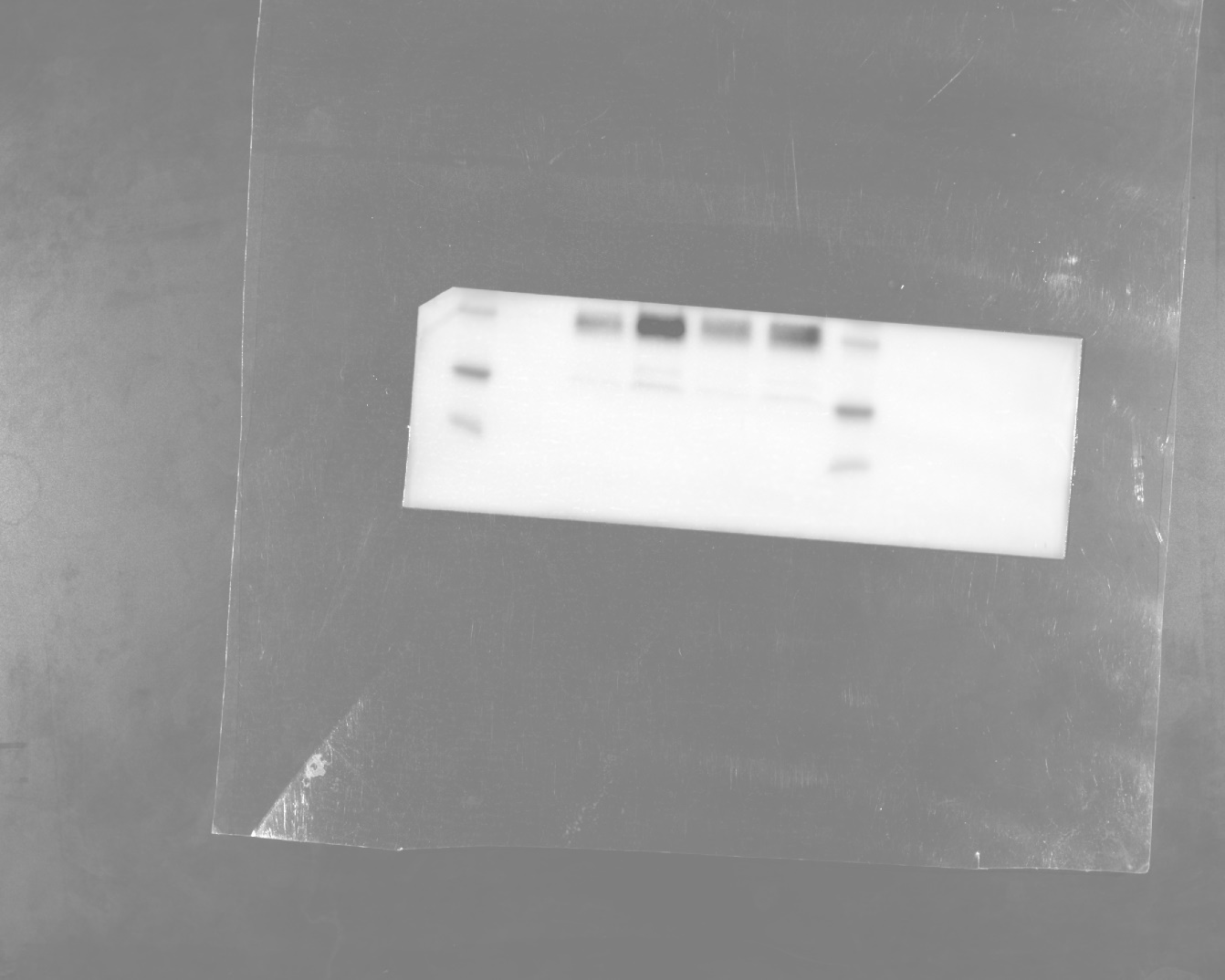


MCL-1


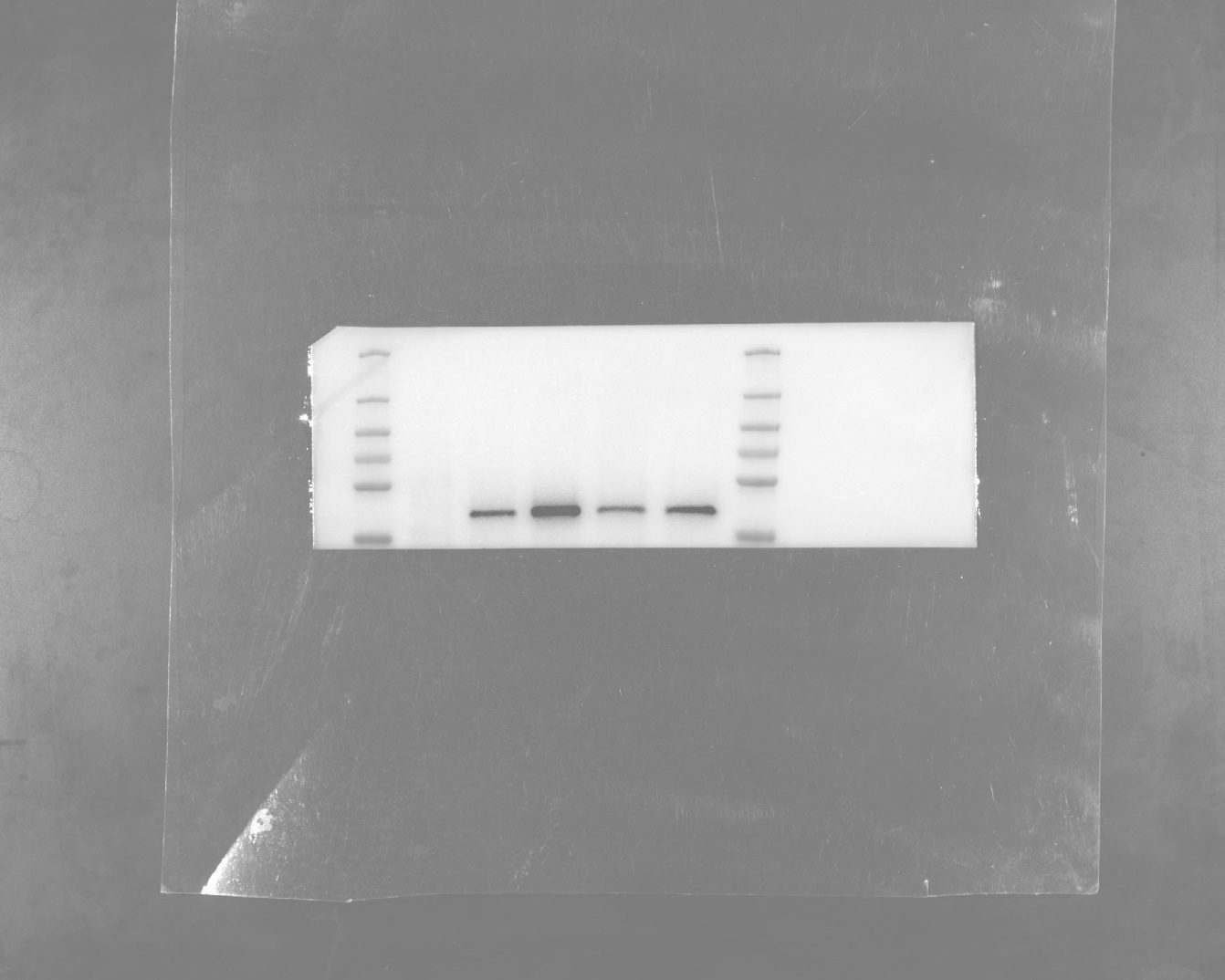


**Figure 4e: Input**


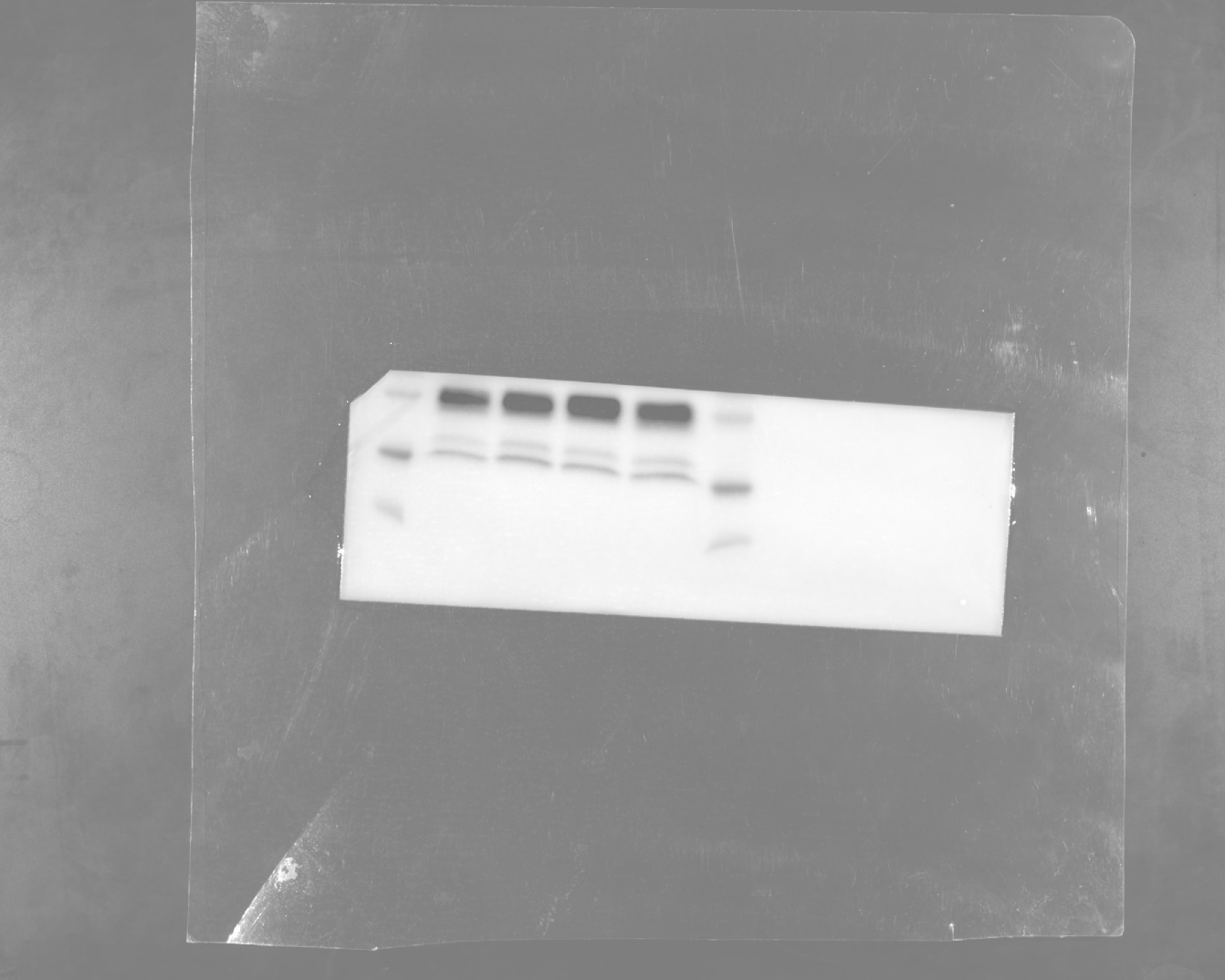
BIM

MCL-1


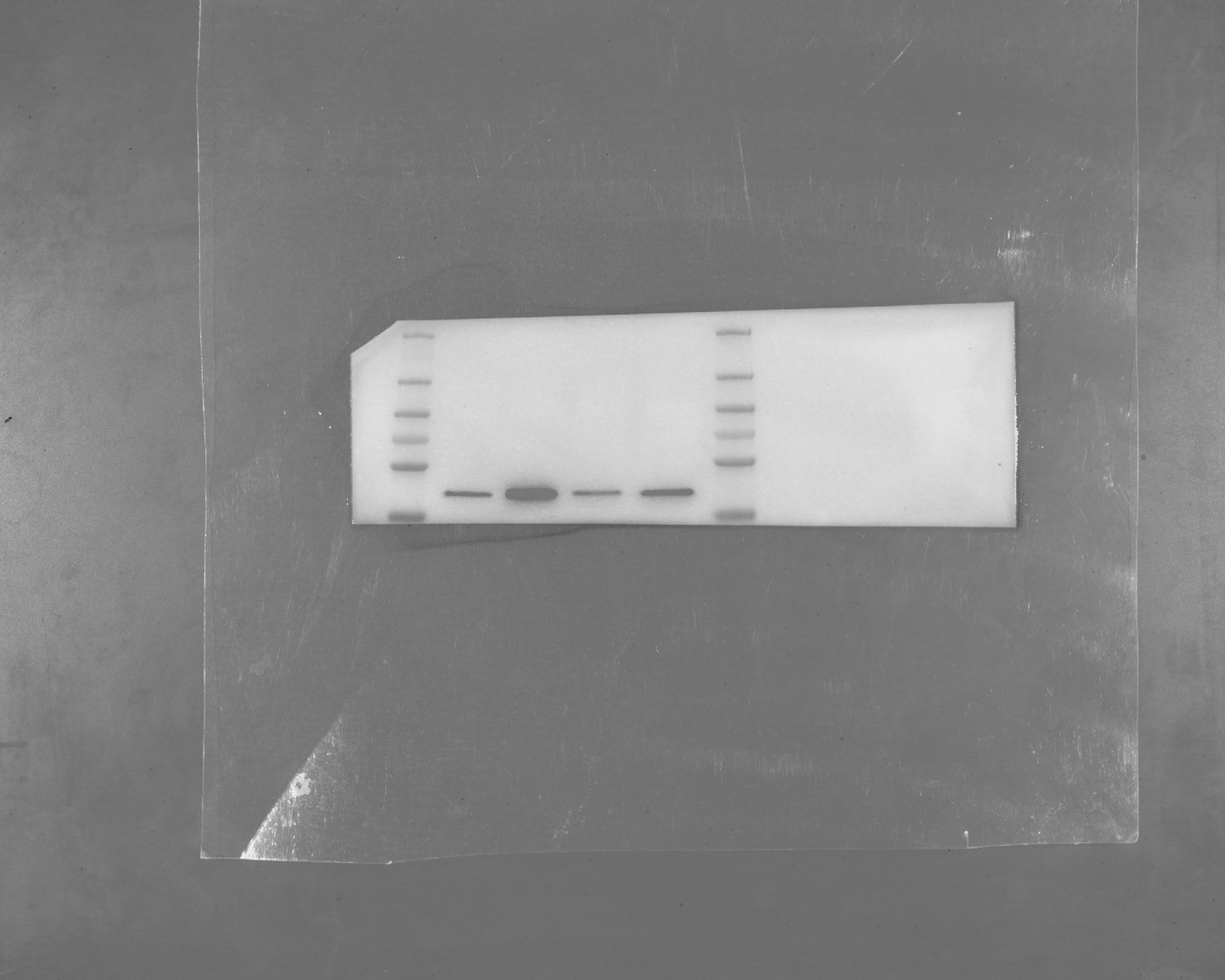


BCL-X_L_


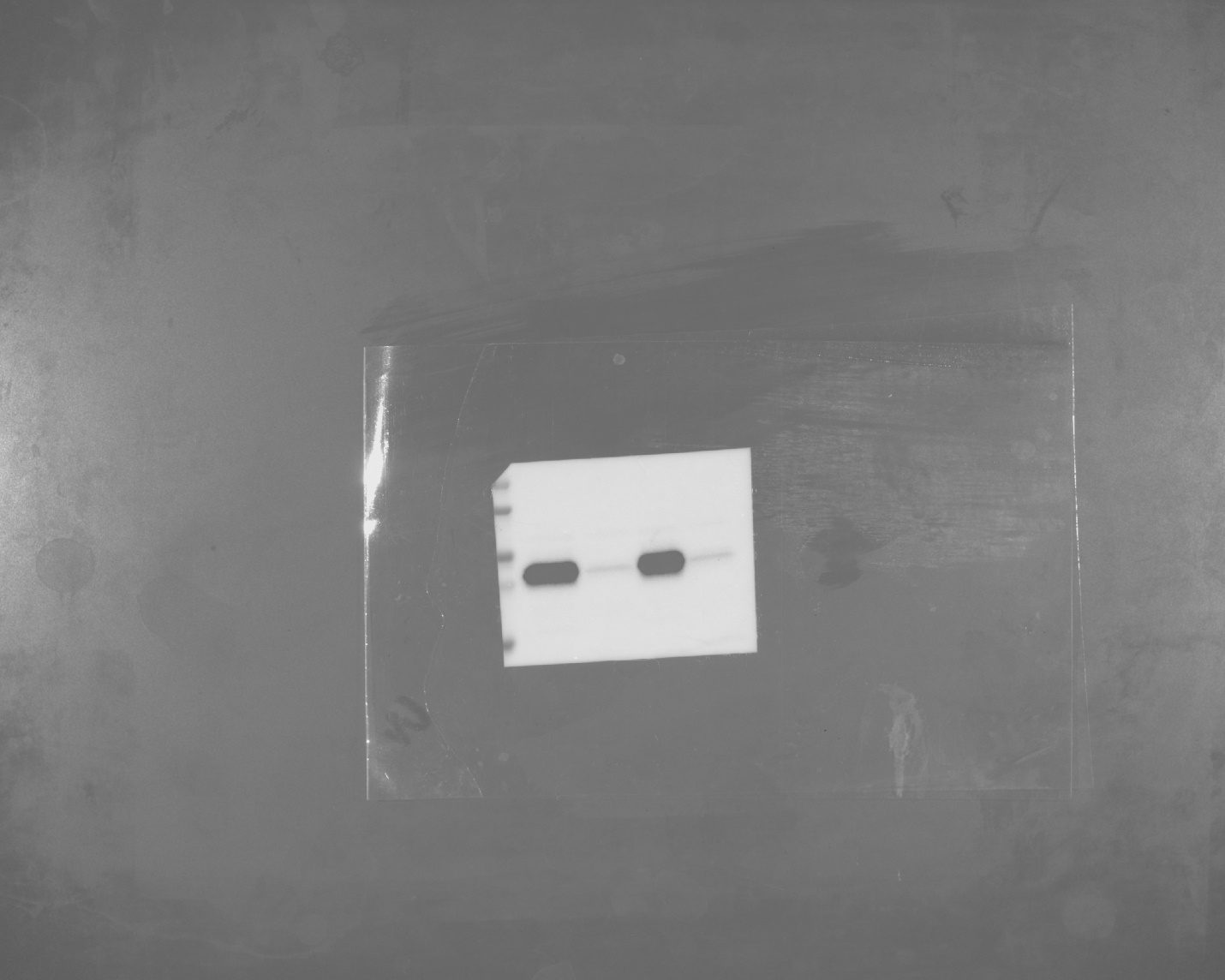


β-Tubulin


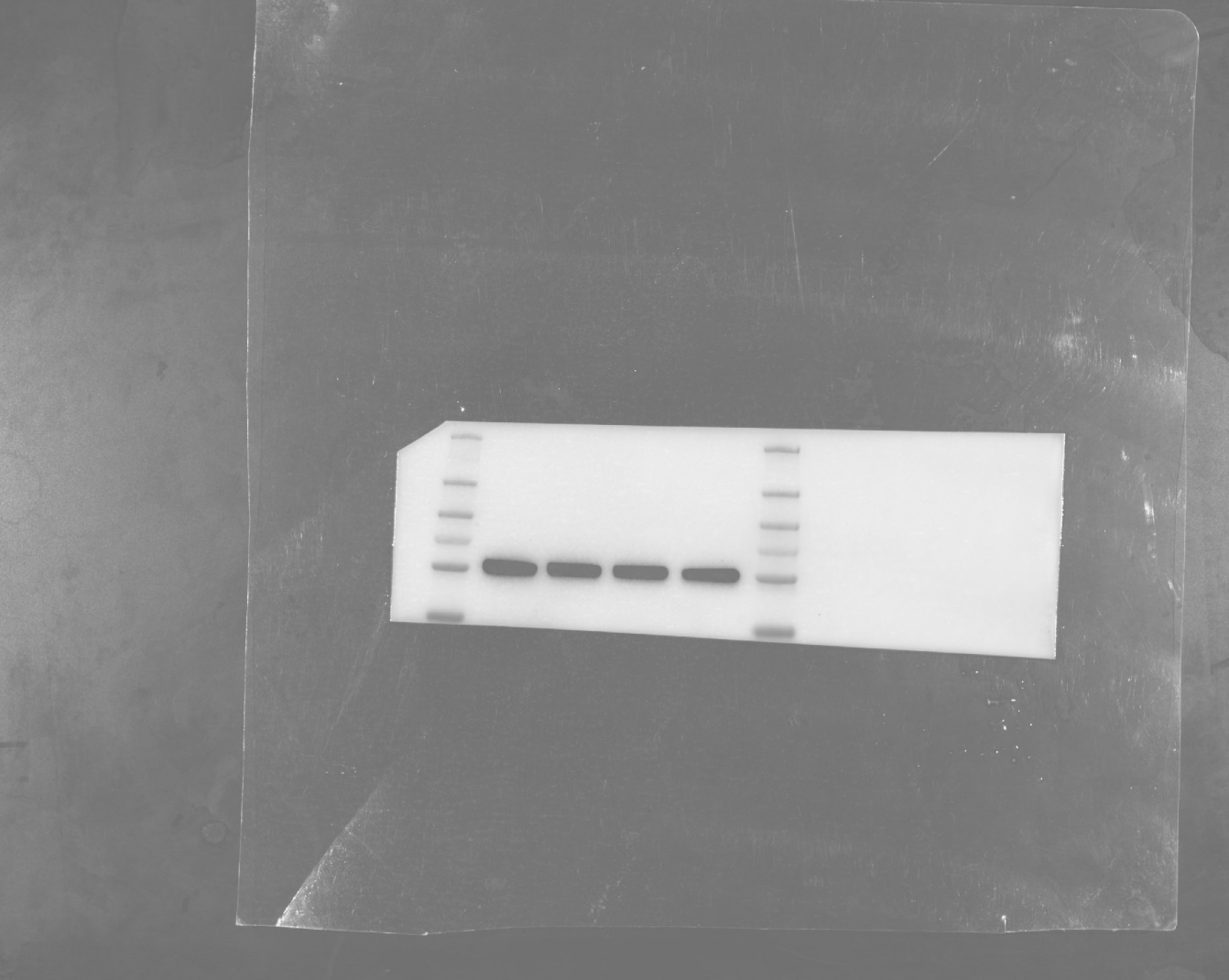


**Figure 5d**

p-4EBP1^T37/46^


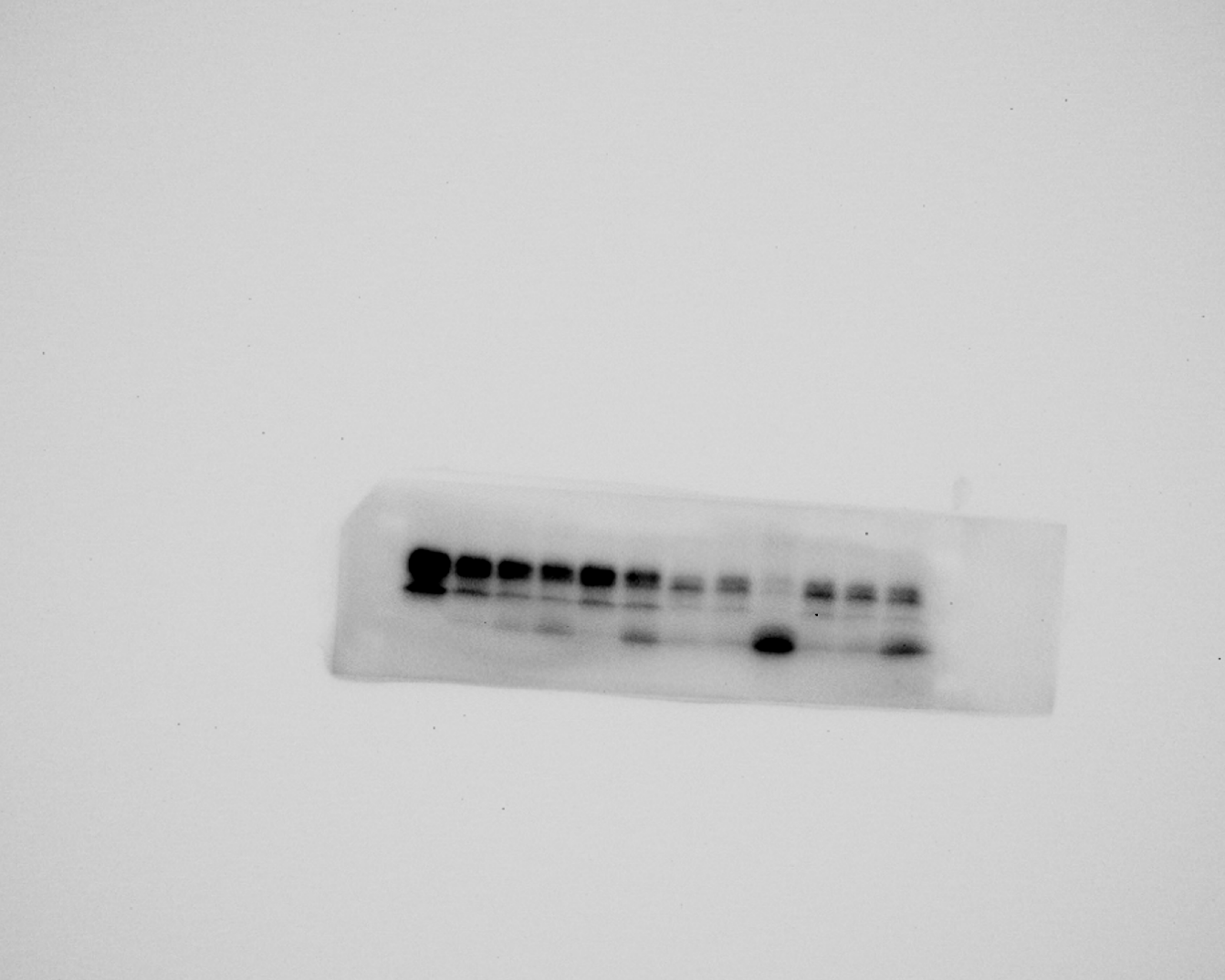


4EBP1


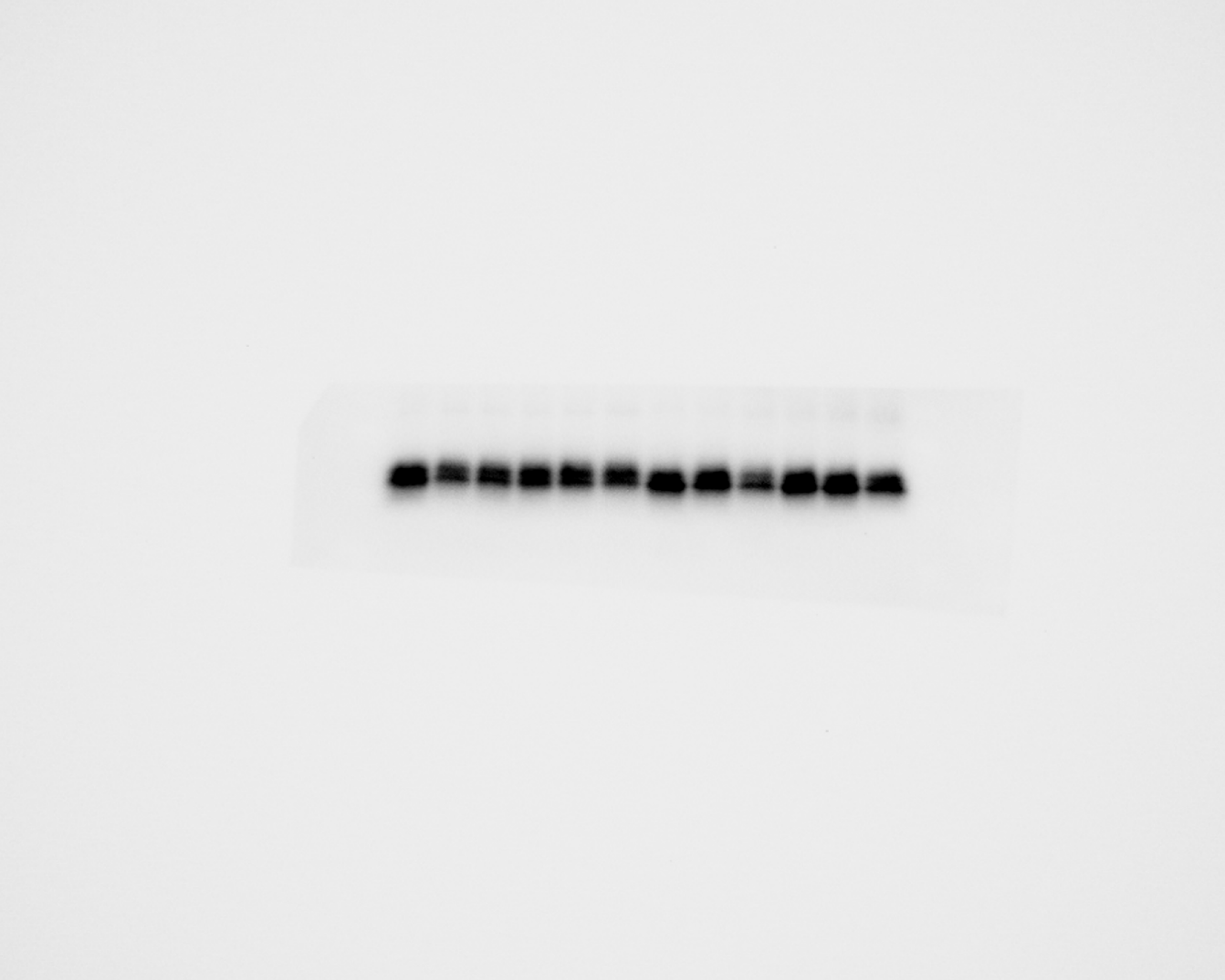


p-S6^S240/244^


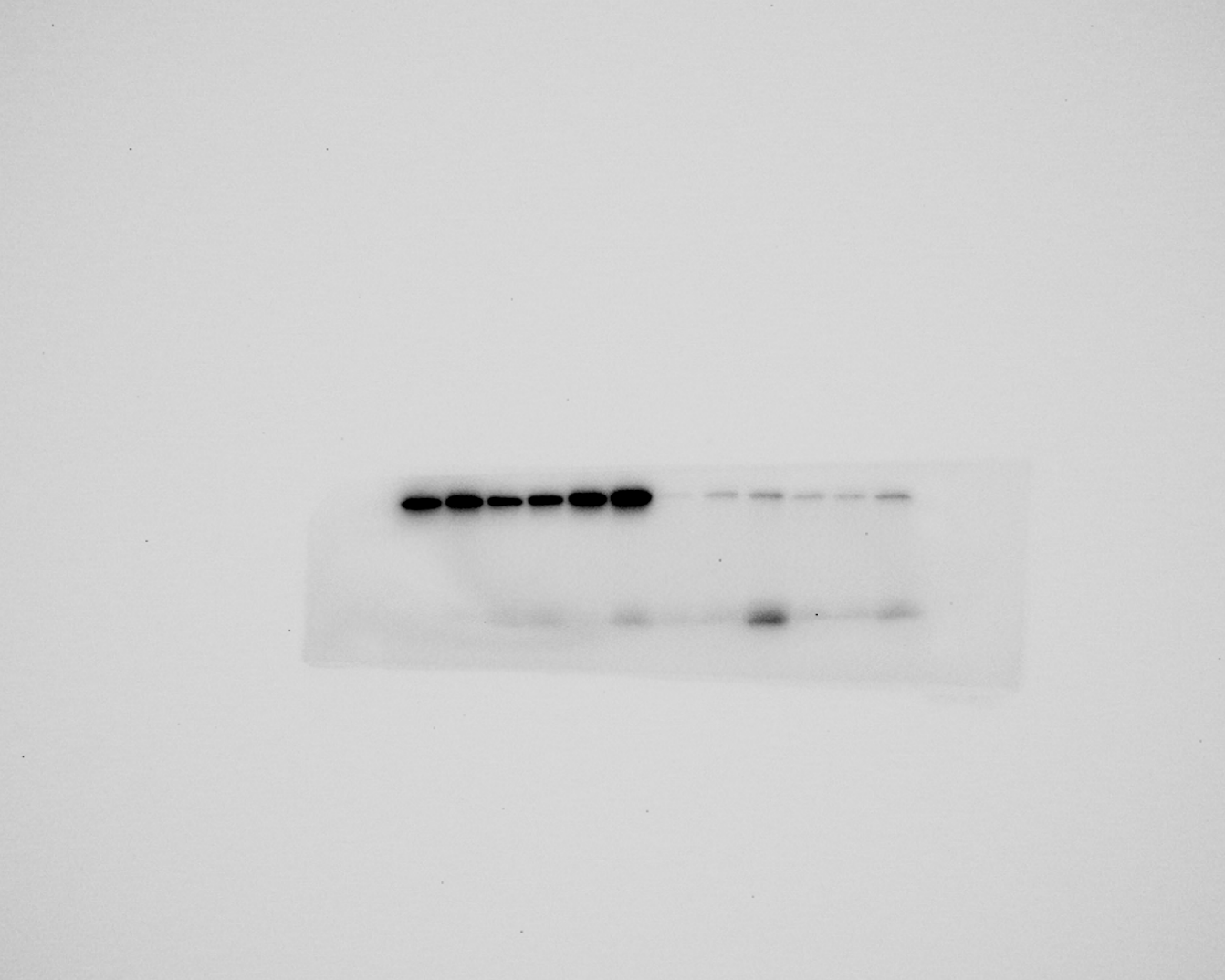


S6


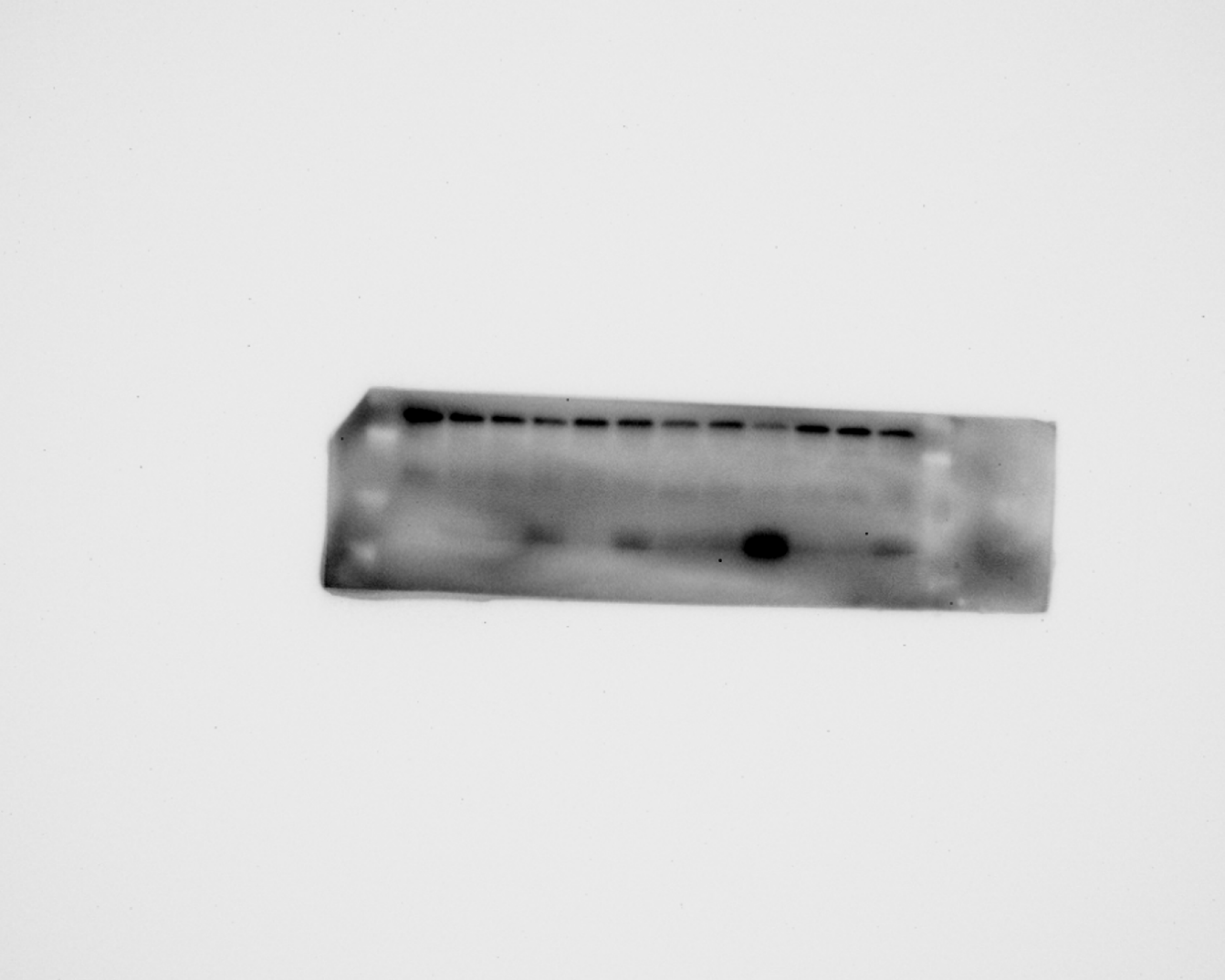


β-Tubulin


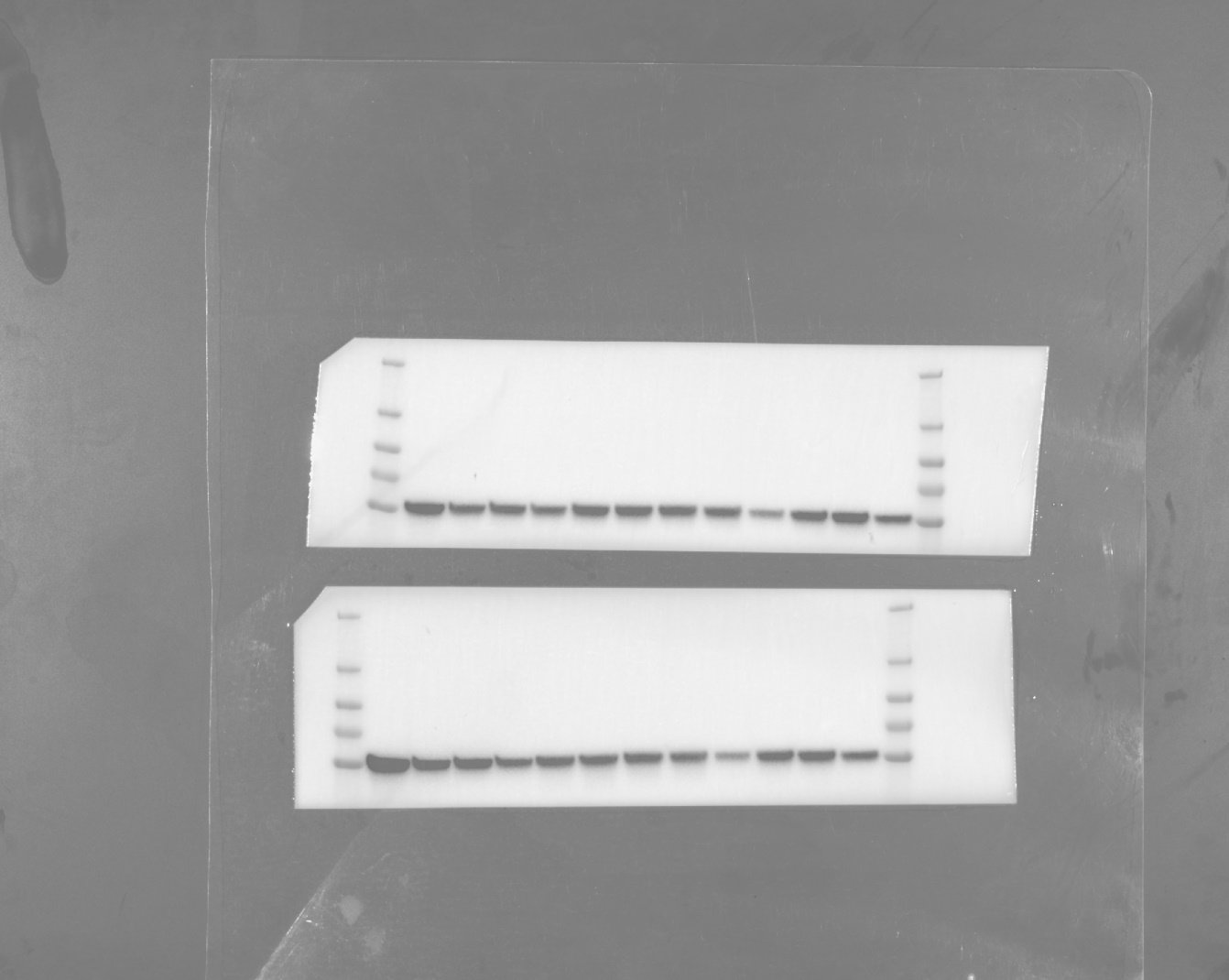


**Figure 5d**

MCL1


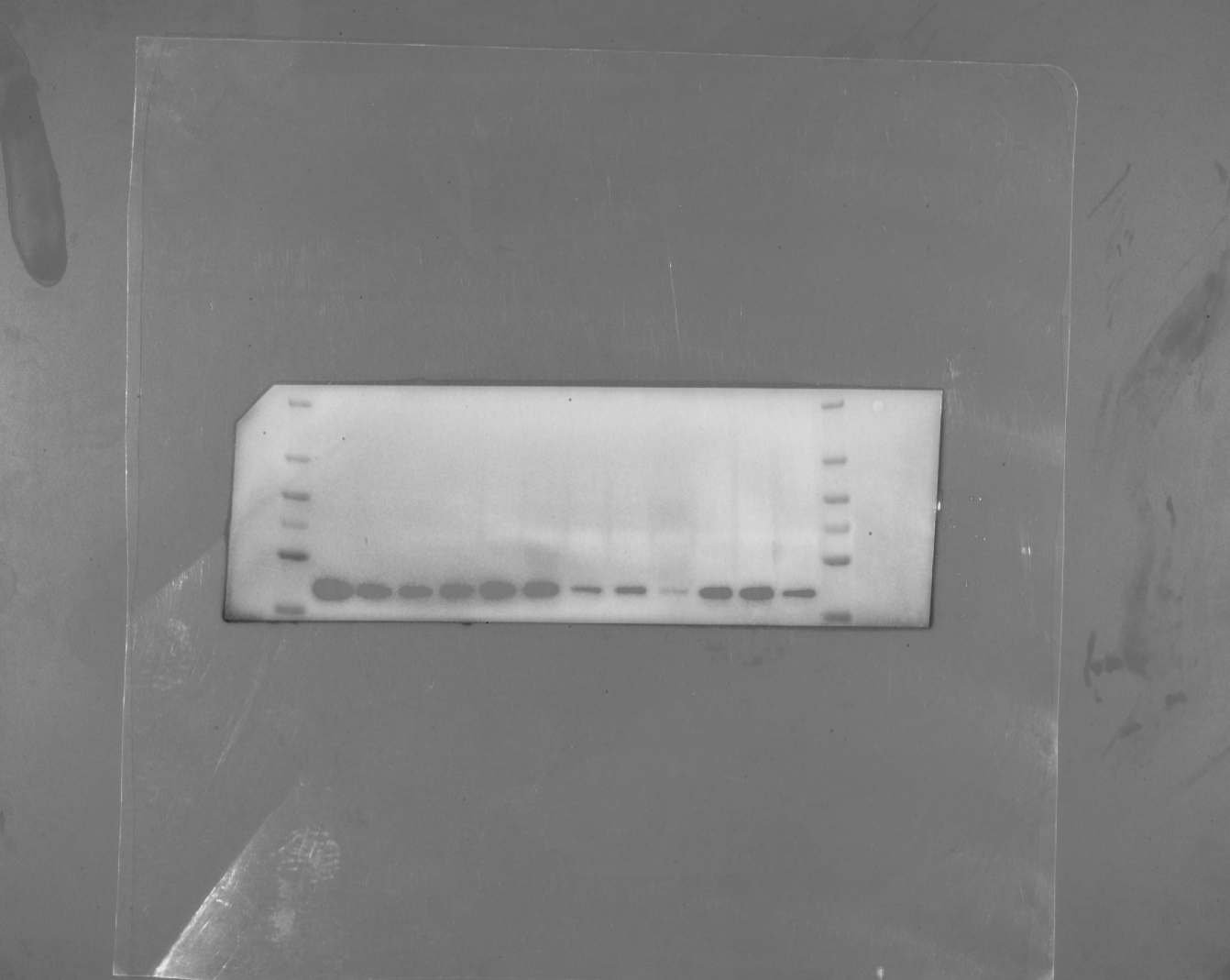


BCL-X_L_


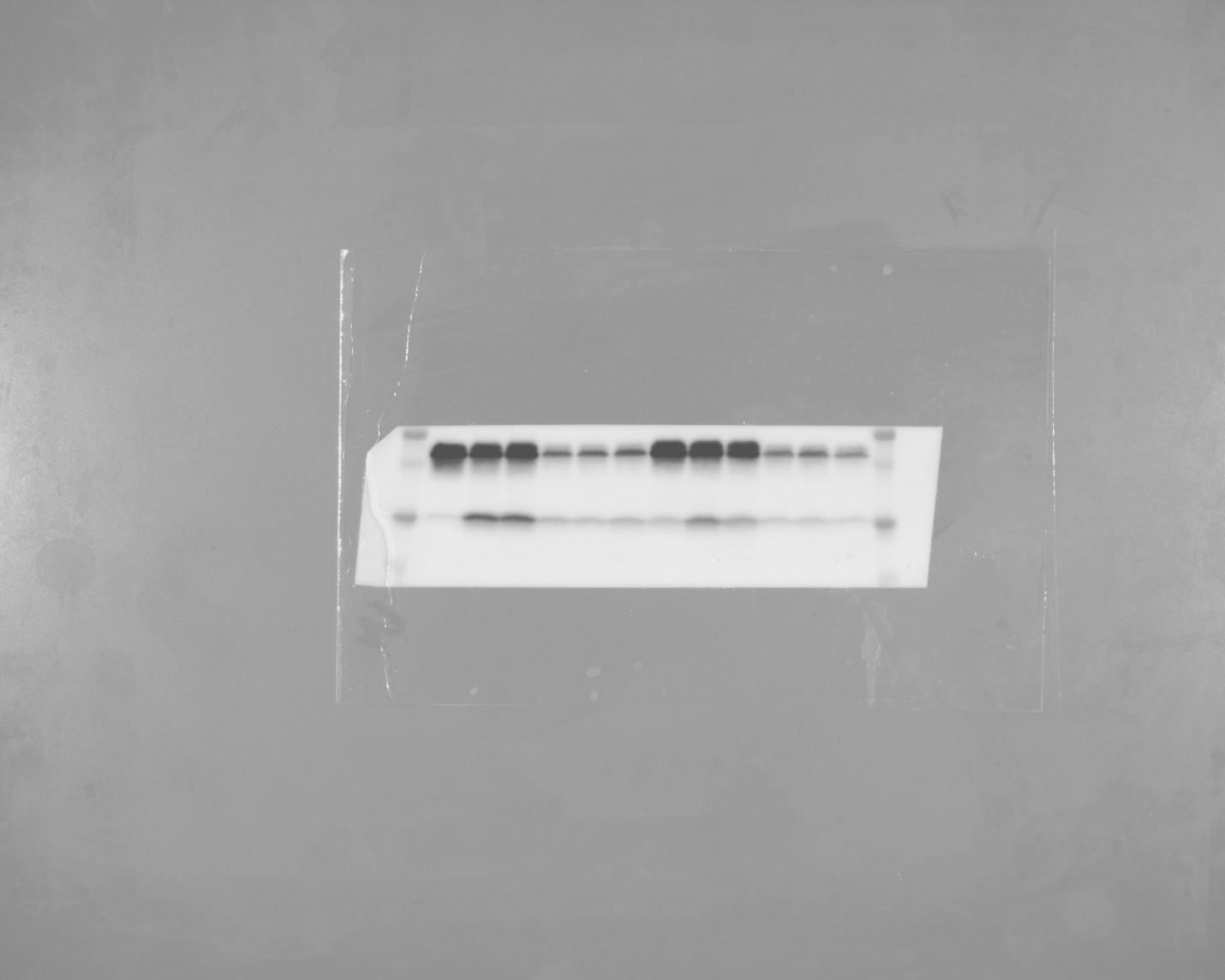


BCL-2


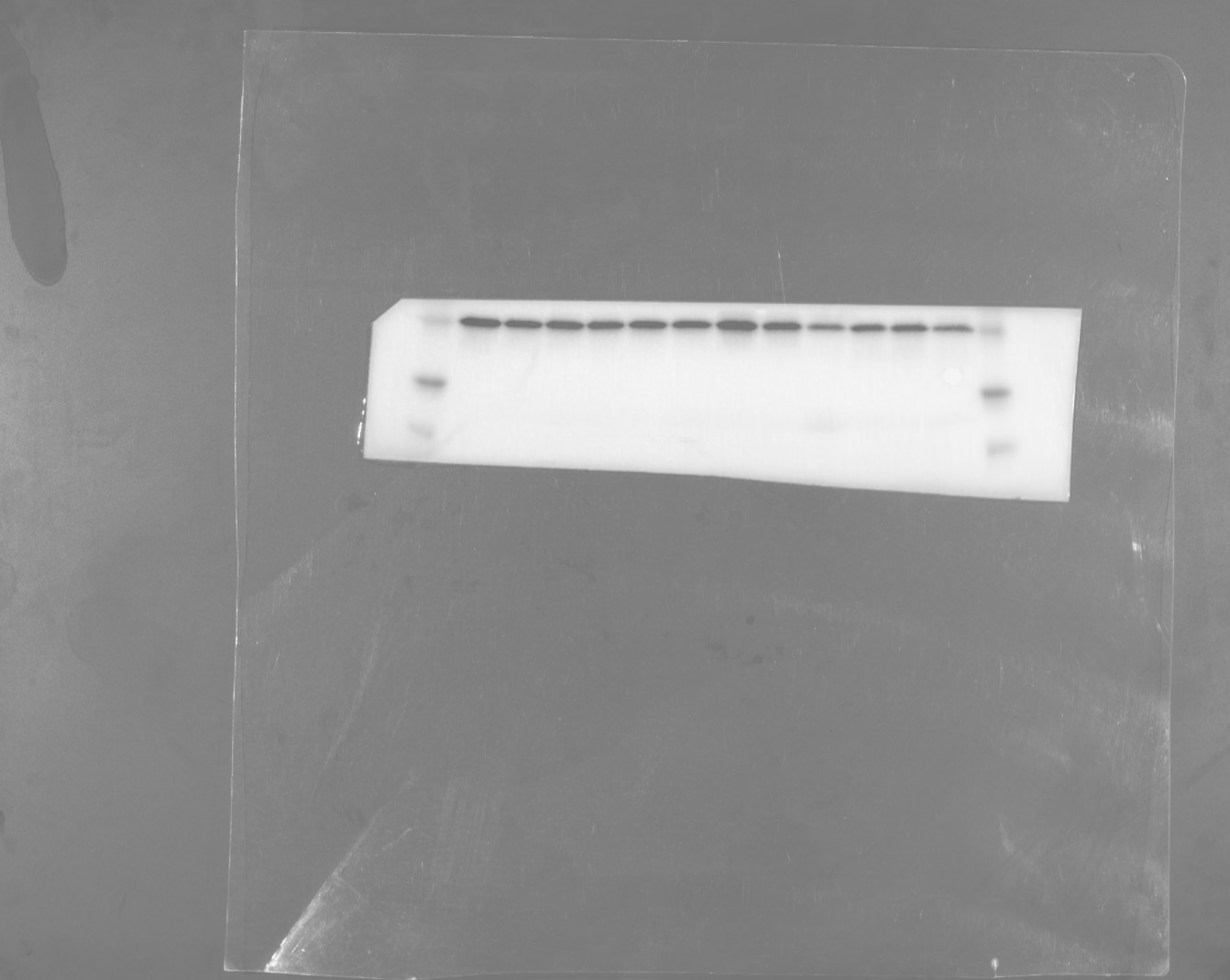


β-Tubulin


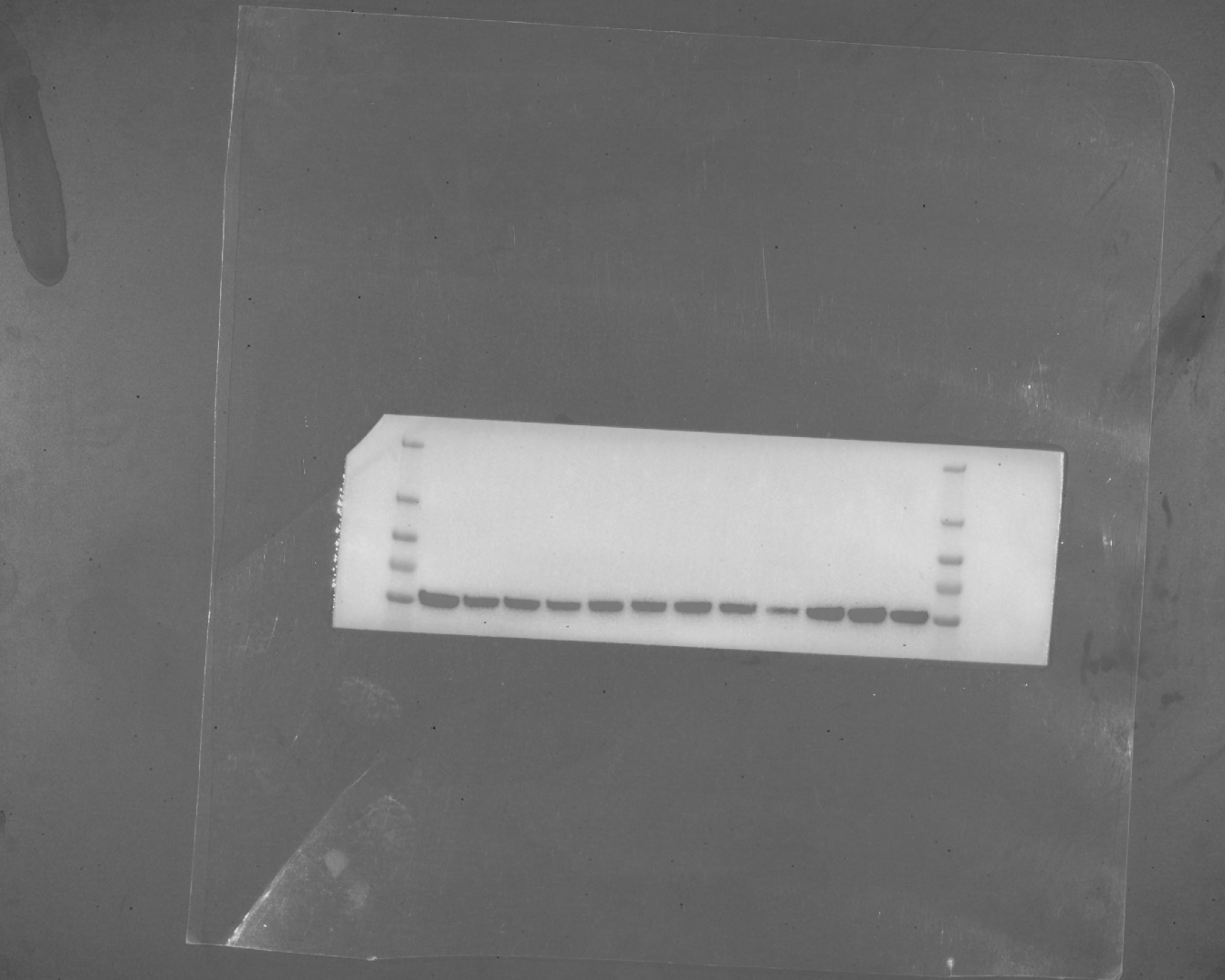

Supplement: Supplementary file 2 — Original Data File [file 41420_2022_1296_MOESM2_ESM.docx]
